# Anthrax revisited: how assessing the unpredictable can improve biosecurity

**DOI:** 10.3389/fbioe.2023.1215773

**Published:** 2023-09-19

**Authors:** Dunja Manal Sabra, Anna Krin, Ana Belén Romeral, Johannes Ludwig Frieß, Gunnar Jeremias

**Affiliations:** Carl Friedrich von Weizsäcker-Centre for Science and Peace Research (ZNF), University of Hamburg, Bogenallee, Hamburg, Germany

**Keywords:** *Bacillus anthracis*, anthrax, biosecurity, bioweapon, bioterrorism, threat evaluation, synthetic biology, converging sciences

## Abstract

*B. anthracis* is one of the most often weaponized pathogens. States had it in their bioweapons programs and criminals and terrorists have used or attempted to use it. This study is motivated by the narrative that emerging and developing technologies today contribute to the amplification of danger through greater easiness, accessibility and affordability of steps in the making of an anthrax weapon. As states would have way better preconditions if they would decide for an offensive bioweapons program, we focus on bioterrorism. This paper analyzes and assesses the possible bioterrorism threat arising from advances in synthetic biology, genome editing, information availability, and other emerging, and converging sciences and enabling technologies. Methodologically we apply foresight methods to encourage the analysis of contemporary technological advances. We have developed a conceptual six-step foresight science framework approach. It represents a synthesis of various foresight methodologies including literature review, elements of horizon scanning, trend impact analysis, red team exercise, and free flow open-ended discussions. Our results show a significant shift in the threat landscape. Increasing affordability, widespread distribution, efficiency, as well as ease of use of DNA synthesis, and rapid advances in genome-editing and synthetic genomic technologies lead to an ever-growing number and types of actors who could potentially weaponize *B. anthracis*. Understanding the current and future capabilities of these technologies and their potential for misuse critically shapes the current and future threat landscape and underlines the necessary adaptation of biosecurity measures in the spheres of multi-level political decision making and in the science community.

## 1 Introduction

Historically, mostly naturally occurring pathogens, such as *B. anthracis* were developed as biological weapons (BWs) due to their inherent infectious and often lethal characteristics (Frischknecht, 2003; Kaufer et al., 2020). The past decades have witnessed an immense increase in the rate of development and research related to life sciences for both industry and academia with applications in all relevant fields. Some of these technological advances and scientific techniques have an exceptional dual-use and hence misuse potential (Lentzos, 2016; Kaufer et al., 2020; Kosal, 2021; World Health Organization, 2022), and could be adapted to develop a new class of advanced BW agents. These can be engineered to elicit enhanced or new effects and alter them to become devastating agents for biological warfare or bioterrorism (Ainscough, 2002; Paris, 2023). However, it is the combination of different technological achievements and developments that together can lower the thresholds for the development of novel biological and chemical weapons.

Multiple national and international legislative regulations such as the Biological Weapons Convention (BWC) provide legally binding measures to prevent the work with biological agents for non-peaceful purposes. Their aim is summarized in the so-called “general purpose criterion”, Article I of BWC, additionally, Article IV obligates states-parties “to prohibit and prevent the development, production, stockpiling, acquisition or retention of the agents, toxins, weapons, equipment and means of delivery specified in Article I of the Convention, within the territory of such State, under its jurisdiction or under its control anywhere.” (United Nations, 1972). Furthermore, multiple export regime controls, such as the Australia Group (AG) (The Australian Department of Foreign Affairs and Trade, 1958) and the Wassenaar Arrangement (Wassenaar Arrangement Secretariat, 1995) have been implemented to prevent the proliferation of dual-use goods and technologies and to promote the transparency of national export control regimes. Moreover, the United Nations Security Council Resolution 1540 (2004) (United Nations Security Council, 2004) obligates states to implement measures against terrorism with nuclear, chemical and biological weapons. With a view to BWs, however, concerns are raised that emerging technologies might serve especially bioterrorists to circumvent existing biosecurity regulations and governance raising legitimate questions about the existing biosecurity landscape (Trump et al., 2021a; DiEuliis, 2022). While such concerns have been raised before, the current threat landscape is more complex than when discussed in 1971 (United States Arms Control and DIsarmament Agency, 1971) or 2001 (Zilinskas, 2020).

Synthetic biology (SynBio) is an emerging technology with many useful applications exemplifying the technological power inherent to biotechnology like the generation of synthetic viruses, bacteria, and eukaryotic cells (Venter et al., 2022), partly synthetic chloroplasts (Miller et al., 2020), the generation of photosynthetically more efficient C_3_-plants (South et al., 2019), or the by now well-known mRNA vaccines (May 2021). However, it is one of the major categories of dual-use research of concern (DURC) for pathogenic microorganisms (MacIntyre, 2015; Sun et al., 2022). With SynBio normally benign microorganisms can be engineered to secrete toxins or even hard-to-obtain regulated pathogens could be assembled in the laboratory (Singh and Kuhn, 2019; Sanz et al., 2022). Genetic modification by editing, deleting, and inserting desired sequences into targeted sites of a genome (Eisenstein, 2020; Hoose et al., 2023; Yeom et al., 2023) by harnessing the clustered regularly interspaced short palindromic repeats (CRISPR)/CRISPR-associated protein (Cas)9 system for genome editing (Jinek et al., 2012; Zhang et al., 2020) may increase the bio-threat potential. In addition, many important biotechnological techniques bear a dual-use and hence misuse potential such as whole-genome sequencing or oligonucleotide synthesis and DNA assembly (assembling multiple smaller fragments of oligonucleotides into the desired larger sequence). Using Golden Gate and Gibson assembly technologies, artificial DNA molecules can be synthesized with greatly reduced cost and time. In fact, the cost of oligonucleotide synthesis has dropped as low as $0.07–0.1 per base and continues to decrease (Sun et al., 2022; Hoose et al., 2023).

Furthermore, such advances do not occur in a vacuum, they are accompanied, supported, and further enhanced by converging technologies from other fields of science. Surely, one of the most influential fields is that of bioinformatics additionally boosted by the advent of artificial intelligence (AI) and machine learning, enabling all branches of omics, biomedical imaging, and signal processing (Min et al., 2016), as well as protein structure prediction (Jumper et al., 2021). Other converging technologies entail robotics relevant for manufacturing and drones, additive manufacturing leading up to 3D bioprinting (Ozbolat et al., 2016), and nanotechnology with application in physics, chemistry, biology, engineering, and medicine (Bracamonte, 2023; Malik et al., 2023; Singh and Kaur, 2023). Furthermore, meteorological data improved critically as an enabling development in biowarfare (Hemming and Macneill, 2020; Levinson, 2022). Taken together, these emerging and converging technologies pave the way for new applications for the weaponization, dissemination, and delivery of biological weapon agents (Brockmann et al., 2019; Kosal and Kosal, 2020; Favaro et al., 2022). Such new agents and BW delivery systems (e.g., drones and advanced aerosolizers) could provide an array of additional and novel use options, expanding the BW paradigm (Pethő-Kiss, 2022) innovative approaches to counterproliferation, detection, mitigation, medical countermeasures, and forensics for attribution. Consequently an adaptation or a change in the biosecurity architecture including biodefense, preparedness, and prevention is necessary (National Academies of Sciences, 2018; Trump et al., 2021b).

Indeed, thus far mostly state actors have been applying advanced technologies for weapons production, at least in past programs (Caudle et al., 1997; Riedel, 2004). Hence, traditionally, concerns over the misuse of, for example, genetic engineering have focused on state-sponsored biological warfare programs possessing the necessary high level of knowledge, skills, and resources to accomplish this challenging and multifaceted task. However, the increasing affordability, widespread distribution, as well as efficiency and ease of use of DNA synthesis and together with rapid advances in genome-editing and synthetic genomic technologies lead to an ever-growing number and types of actors who could potentially misuse existing knowledge and emerging technologies (Hoffmann et al., 2023; Paris, 2023). Therefore, as the field advances, BW are expected to become a larger concern as they could be misused by malicious non-state actors, because scientific advances will make use of biological agents more accessible (Sanz et al., 2022; Yassif, 2022). In the past, organized non-state groups and potential adversaries demonstrated they can acquire dangerous biological agents if sufficiently determined. Therefore the focus of the present manuscript is bioterrorism. In fact, there have been several confirmed cases of biological agent events between (Carus, 2001). Noteworthy, the influence of scientific progress in relevant fields on the likelihood of bioterrorists attaining and using BWs can not be quantitatively determined. Past terrorists’ failures to develop and use BWs indicate that developing a BW is a highly intricate process. Thus, the impact of a single scientific breakthrough or a novel technology on BW acquisition should not be overstated (Koblentz, 2020). Understanding the current and future technology capabilities and their misuse potential is critical for understanding the current and future threat landscape, i.e., biodefense and biosecurity. New bioagents could emerge or be developed much faster than defenses against these threats can be built.

Biological weapons do not only pose a threat through state-sponsored programs but also in bioterrorism and bio-crime incidents. (For distinction please refer to Jansen et al. (Jansen et al., 2014). This paper focuses on *B. anthracis* as a biological weapon agent. The zoonotic bacterial pathogen *B. anthracis* is the etiological agent of peracute, acute, subacute and chronic anthrax, an often fatal toxin-mediated disease primarily affecting herbivores, but also encountered in other mammals, including humans, and occasionally birds (Turnbull, 2014). Mostly in poor rural areas, up to 2,000 (WHO, 2023) cases (Hesse et al., 2022; WHO, 2023) of anthrax occur annually worldwide (Carlson et al., 2019). Although, *B. anthracis* and its persistent endospores in the soil generally do not pose a public health concern in post-industrial societies, it is one of the high-priority and most dangerous BW agents. It is thus classified by the Centers for Disease Control and Prevention (CDC) as a category A agent, posing the highest risk to the public and national security because of its widespread availability, environmental stability, easy dissemination, its morbidity and mortality, and consequently the high potential for social disruption (Rotz et al., 2002; Riedel, 2005; Cole and Bergman, 2010; Morse, 2014; Johns Hopkins Center for Health Security, 2023). Among the three major forms of human anthrax (cutaneous, gastrointestinal, and inhalational), cutaneous anthrax is the most common with a 80% survival rate even if untreated. The form most likely resulting from an aerosolized spores is inhalational anthrax. Prior to 2001, it was believed that inhalational anthrax would lead to 90% of fatal cases. However, in the anthrax attack of 2001, with prompt recognition and treatment with appropriate antibiotics, the fatality rate was reduced to 5 out of 11 anthrax victims.

Phylogenetically *B. anthracis* belongs to the *B. cereus sensu lato* group consisting of 18 closely related sporulating Gram-positive bacteria including *B. cereus* and *B*. *thuringiensis* (Acevedo et al., 2019)*.* Despite their highly divergent pathogenicity, the chromosomes of these three species show very high genetic similarity, while their rRNA sequences are nearly identical, showing only varaiations expected within different species (Bazinet, 2017). *Bacillus thuringiensis* infects insect larvae, while *B. anthracis* and *B. cereus* are mammalian and human pathogens. While anthrax is often fatal, *B. cereus* is a opportunistic pathogen causing periodontitis, foodborne illness, and acute ophthalmitis in humans (Kotiranta et al., 2000; Argôlo-Filho and Loguercio, 2013; Granum, 2017; Pilo and Frey, 2018). Some bacterial strains of *B. cereus,* e.g., *B. cereus biovar anthracis*, which are ubiquitous in West Africa, cause an anthrax-like disease in a broad host range of mammals (Pilo and Frey, 2018). So far, no cases of human infections with this strain have been reported. Nonetheless, the CDC has included this pathogen in the list of Biological Select Agents and Toxins (BSAT) posing a potential risk to public health and safety (American Society for Microbiology, 2017). To the best of our knowledge, such an amendment for *B. cereus biovar anthracis* is currently lacking in the European Union (EU) regulations on dual-use items.

Using the most thoroughly studied traditional BW agent *B. anthracis* (Savcı, 2019) as a prime example, this paper analyzes and assesses the possible bioterrorism threat arising from advances in synthetic biology and other converging sciences. In addition, the possible required biosecurity adaptations in the field of biodefense are identified. Creating effective biosecurity procedures will require understanding the present state of synthetic biology and other biosecurity-relevant emerging technologies. For a realistic harm potential and threat assessment of a future *B. anthracis* BW, it is necessary to weigh and reassess identified hazards and novel threats against established mitigation measures and possible countermeasures. This includes, on the one hand, knowing the platforms and technologies available for construction or engineering *B. anthracis* or related microbes, and planning for the future when the field overcomes bottlenecks or barriers. On the other hand, effective biosecurity requires continuous technology mapping to identify possible *B. anthracis* dissemination routes, its potential targets and the ability to apply forensics for attribution after an attack. This article addresses a problem on the intersection of life sciences and security studies and is hence written from a transdisciplinary perspective.

## 2 Materials and methods

Our applied methodology falls under the umbrella of foresight methods defined as “a systematic, participatory, future-intelligence- gathering and medium-to-long-term vision-building process aimed at enabling present-day decisions and mobilizing joint action” (Miles et al., 2016; Foresight, 2018). Foresight methodology is applied to encourage the analysis and consideration of a range of future biosecurity hazards arising from contemporary advances in synthetic biology and other technologies to inform decision-making and public policy (OECD, 2019).

**FIGURE 1:**
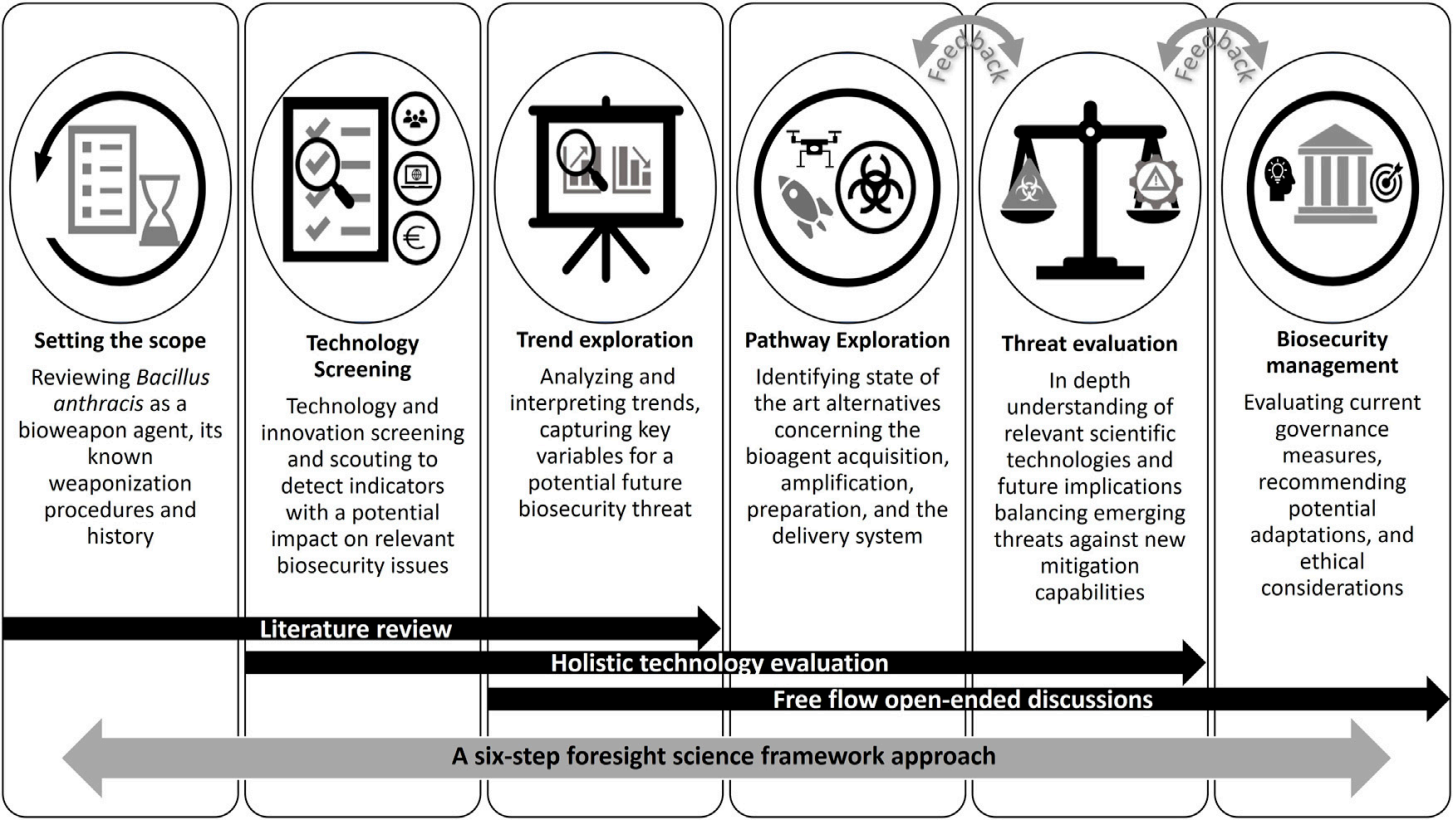
A conceptual six six-step foresight science framework approach.

For the current study, we have developed a conceptual six-step foresight science framework approach as depicted in Figure 1. This framework was adapted from biosecurity and anti-bioterrorism studies and represents a synthesis of various foresight methodologies implementing literature review, and elements of horizon scanning, trend impact analysis, red team exercise (Zhang and Gronvall, 2020; Moran, 2021) and free flow open-ended discussions. We have chosen this framework because it builds on existing knowledge of historical anthrax attacks and analyzes the possible future implications of a changing scientific and technological environment for *B. anthracis* BW development and employment. This is a prerequisite to proactively deterring or defeating future threats by exposing vulnerabilities and allowing for corrective actions. In addition, it allows evaluation of whether advances in science and technology may enhance the possibility of malicious actors gaining access to the required knowledge and scientific infrastructure to develop and use an anthrax BW. This information is required for threat analysis, that in turn could reveal possible deficiencies in the current biosecurity management system.

**FIGURE 2:**
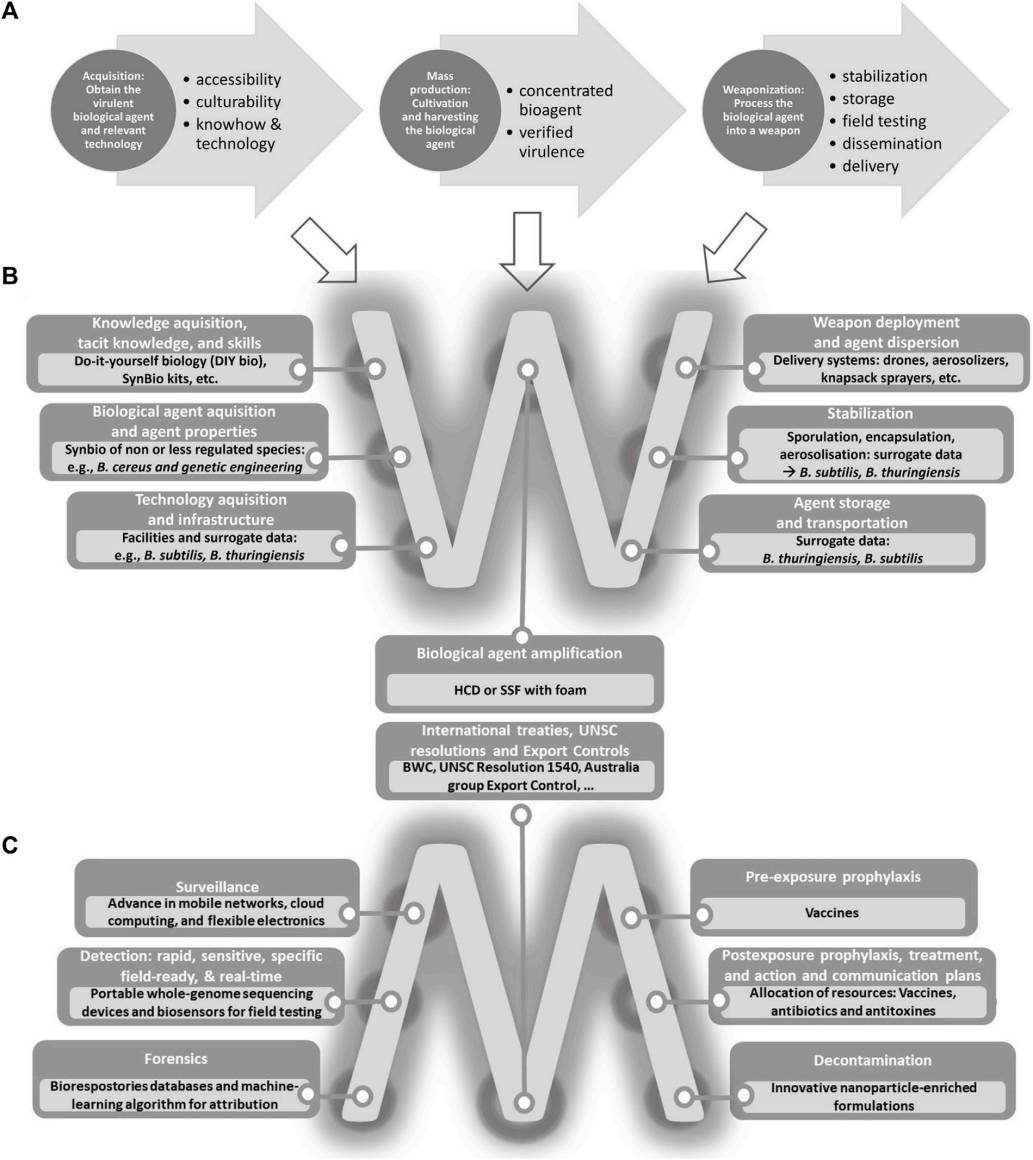
Process of the pathway exploration of a biological weapon. **(A)** Technical steps for the development of BWs. Implications of scientific developments, public accessibility of biology, knowledge, and emerging as well as enabling technologies for **(B)** clandestine development of a *Bacillus anthracis* BW; and **(C)** developing measures for monitoring, medication, and mitigation. UNSC: United Nations Security Council; HCD: High-Cell-Density; SSF: Solid-State Fermentation.

By reviewing over 600 publications, the historical development of *B. anthracis* in BW programs and its potential use as a modern biological weapon agent driven partly by advances in biosciences will be traced to set the scope. Of special interest is the literature on the anthrax bioagent including methodology to genetically engineer *B. anthracis* and related strains, different delivery systems, international BW governance and mitigation strategies, but also export control laws, and agent detection methodology. Furthermore, the literature research served the purpose of feeding into steps of technology screening and qualitative trend extrapolation in identifying indicators and structural trend shifts, respectively. While indicators identified by the technology screening are hinting at potential future tendencies either in the form of basic research, patents, investments, or among others social phenomena (Amanatidou et al., 2012), trend extrapolation, on the other hand, focuses on established ongoing dynamics such as trends and driving forces. Technology screening and trend extrapolation were performed to elucidate how contemporary dynamic science developments especially in the field of synthetic biology could facilitate new biosecurity challenges (Bakhtin et al., 2017; Kohler, 2021). In the pathway exploration special emphasis was given to the current technology advancements or knowledge availability and accessibility with relevance to weaponizing. In this study, we examine the current technical obstacles and possibilities a terrorist group may encounter in the development of an anthrax bioweapon. Therefore, in this thought experiment we researched and analyzed every necessary step concerning the bioagent acquisition, amplification, sporulation and aerosolization as well as the delivery system, choosing the most economic development options and those that pose the lowest possible danger for perpetrators to be exposed to the agent during the production process (Figure 2A). Subdividing the process into the necessary labor steps (Figure 2B) helped to investigate potential loopholes and regulatory gaps. Each step (e.g., different acquisition paths to attain a virulent anthrax strain) is described in its difficulties and possibilities, potential bottlenecks, and circumventive alternatives. The goal of pathway exploration is to identify weaknesses and vulnerabilities in systems or strategies, develop more effective plans and processes, and prepare organizations to respond to unexpected challenges and threats.

Thereafter, a threat evaluation of identified hazards and novel threats was performed. The aforementioned foresight science framework steps allowed a holistic technology evaluation and hence an out balancing against established monitoring, medication and mitigation measures and possible countermeasures (Figure 2C) for a potential future *B. anthracis* BW threat evaluation. Free flow open-ended discussions led to suggesting the necessary biosecurity architecture adaptation for appropriate biosecurity management including required measures to raise awareness and preparedness. In addition, recommendations for politicians and other stakeholders were elucidated.

## 3 Results and discussion

For the conceptual six-step foresight science framework approach the most important findings were considered (listed in Supplementary Table S1). For the sake of convenience, a chronological-analytical representation of these findings is given below.

### 3.1* Bacillus anthracis* in warfare

Not surprisingly, anthrax as a BW agent has been the focus of BW research for at least 11 decades (World Health Organization, 1970; Centers for Disease Control and Prevention, 2023). Being a traditional, non-genetically engineered BW agent, *B. anthracis* has reliable traits regarding pathogenicity and is capable of causing lethal inhalation anthrax. In addition, it is characterized by its agent availability; large-scale manufacturability; stability during production, storage, and transportation; ability to be efficiently disseminated including stability after dissemination limited vaccine availability; and previous research and development of the agent as a BW; with the potential of causing public panic and social disruption. Hence, it fulfills all the requirements of a BW agent except for lacking person-to-person transmission.

At least 8 nations are believed or known to have had developed offensive biological weapons programs that include *B. anthracis* until 1990s (Riedel, 2005; Carus, 2017). Indeed, already in World War I, Germany used anthrax to infect animals (Leitenberg, 2001). During World War II, *B. anthracis* was found in most military BW arsenals. The infamous, Japanese Unit 731 tested anthrax among other BW agents on Chinese prisoners during the occupation of Manchuria (Riedel, 2004; Riedel, 2005). Until the BWC entered into force (1975), the United Kingdom (UK), the United States of America (United States), Japan, and the Soviet Union (USSR) weaponized *B. anthracis* within their military programs, even if an anthrax BW battlefield employment never happened (Bernstein, 1987; Roffey et al., 2002; Cole and Bergman, 2010; Beedham and Davies, 2020; Centers for Disease Control and Prevention, 2023).

An accidental release of anthrax spores in 1979 from the military research and production facility in Sverdlovsk, USSR exposed the deadly impact of this bioagent. At least 68 people died in the ensuing anthrax outbreak (Meselson et al., 1994). In addition, this accident showed that the USSR continued clandestine research on *B. anthracis* during the Cold War, even after the signature of the BWC In the same year, an anthrax vaccine precipitated (AVP) was licensed in the UK (Splino et al., 2005).

During this period, the scientific fields of molecular biology and microbiology, and other technologies were advancing at a very high pace. Two decades after the initiation of the BWC, the Third Review Conference in 1991 decided to establish an Ad Hoc Group of Governmental Experts (Final, 1991) that held four sessions in 1992 and 1993 to identify and examine potential verification measures from a scientific and technical standpoint. Eventually, by the end of the millennium, secret military programs including *B. anthracis* developed, e.g., by Iraq (Zilinskas, 1997; Cole and Bergman, 2010) was terminated by UNSCOM and UNMOVIC and those of USSR (Meselson et al., 1994) were allegedly terminated (Mauroni, 2022) but never veryfied. In addition, the anthrax vaccination of troops and the veterinary vaccine for livestock seemed to contain the danger of. In addition, the anthrax vaccination of troops and the veterinary vaccine for livestock seemed to contain the danger of *B. anthracis* in biological warfare.

### 3.2 *Bacillus anthracis* in bioterrorism

At about the same time, besides state actors and their BW programs, a new danger in the form of bioterrorist-related inhalation-anthrax attacks emerged, signaling the beginning of a new era. Foremost, terrorists tried to use anthrax as a BW. Aum Shinrikyo cult released *B. anthracis* spores in an unsuccessful biological attack in Kameido, Japan wanting to initiate an inhalation anthrax epidemic. Cult members successfully designed and built a system for pumping a bacterial suspension up eight floors of their head office building to an aerosol dispersal device on the rooftop (Keim et al., 2001). However, this and several other attempts with anthrax spores failed, due to the use of an attenuated Sterne strain, also used as a vaccine for animals (Cole and Bergman, 2010). To date, only a small fragment of the cult’s program was uncovered by Japanese police and intelligence, and only parts of evidence have been made publicly available (Riedel, 2004).

In the mid-1990s al-Qaida allegedly underwent attempts to procure and weaponize anthrax bacteria, with the former USSR, Kazakhstan or East Asia as a source of these biological agents. According to United States officials in Afghanistan in late 2001, efforts to weaponize *B. anthracis* failed despite speculated assistance from Russian scientists (Cronin, 2003; Spyer, 2004; Leitenberg, 2005; Salama and Hansell, 2005).

Almost concomitantly in the fall of 2001, letters containing anthrax spores dispatched to high-profile journalists and politicians in the United States killed five non-targeted people, mostly postal workers (Quintiliani and Quintiliani, 2003). A nearly decade-long, $100 million investigation into the 2001 Amerithrax attacks, proved *B. anthracis* mass disrupting capabilities as well as the difficulty associated with investigating such incidents (Böhm and Beyer, 2003; Cole and Bergman, 2010).

### 3.3 Technology screening and trend extrapolation

Considering anthrax research, we identified several indicators in different pertinent fields with a dual-use potential relevant to anthrax BW development (Supplementary Table S1). The indicators most relevant to our pathway exploration were scientific achievements, advancements or discoveries that could be exploited for *B. anthracis*’ BW attribute enhancement or those potentially used to circumvent biosecurity measures implemented to prevent the proliferation and development of a *B. anthracis* BW. In our qualitative approach extrapolating indicators along their trajectories, five major trends were identified as relevant (from most to least significant).

1. Increasing access to standardized biotechnology potentially reduces tacit knowledge requirements (Jackson, 2001; Revill and Jefferson, 2014)
2. Accessibility of scientific data (open access publications, online repositories, literature databanks) is continuously increasing (euroCRIS, 2016)
3. Oligonucleotide synthesis and sequencing are facilitated, readily available and steadily decreasing in costs (Hughes and Ellington, 2017; Hoose et al., 2023)
4. Converging and enabling technologies (Internet, AI, Machine Learning, Additive Manufacturing, unmanned aerial vehicles (UAV), Robotics, and advances in aerosolizing technology) expand the BW paradigm (Brockmann et al., 2019; Lentzos, 2020)
5. The Do-It-Yourself (DIY) and Frugal Science community expands and facilitates communication and protocol design and exchange (Seyfried et al., 2014; Tocchetti and Aguiton, 2015; Tennenbaum et al., 2021)

These trends project impacts that increase future threat potentials by further lowering entry obstacles for BW development, reducing the risk of being detected and uncovered, facilitating BW design and mass production, as well as employment. Potential threats are more closely examined in the pathway exploration. At the same time, these trends might also contribute to strengthening preparedness, prevention, and mitigation.

### 3.4 Pathway exploration

To evaluate the inherent threat posed by contemporary science and the possible new dangers arising from scientific and technological advances, *B. anthracis* BW development stages were thoroughly analyzed. Relevant findings from technology screening and trend extrapolation feed into our pathway exploration. Generally, the development of a *B. anthracis* weapon starts with the acquisition, followed by mass production, and ends with the weaponization of *B. anthracis a*s depicted in Figure 2A.

The following part contains considerations on scientific developments, public accessibility of biology, knowledge, and emerging as well as enabling technologies for the clandestine development of a *B. anthracis* BW by terrorists (Figure 2B).

#### 3.4.1 Knowledge acquisition

The deliberate, malicious misuse of biosciences and technology, besides a motive, requires intention and material resources including technological infrastructure, access to information as well as necessary explicit and tacit knowledge (Vogel, 2006; Nixdorff, 2020). Since the turn of the millennium, there exists an apprehension that terrorists or other state- or non-state actors might circumvent existing biosecurity regulatory systems by acquiring new tacit knowledge, expertise, or vulnerabilities to develop biological weapons (Riedel, 2005; Cole and Bergman, 2010; Mondange et al., 2022). Explicit knowledge includes standard operation procedures for producing and processing biological agents and can be acquired through open-access scientific literature. In fact, a vast amount of knowledge and information from many decades of anthrax research is available and publicly accessible (Morris and Boyack, 2005; Savcı, 2019). Undeniably, globalization and the internet have significantly diminished the barrier to acquiring explicit knowledge. Tacit knowledge on the other hand is not only acquirable through hands-on encounters but it remains a hindrance to weapon efficiency and effectiveness (Tennenbaum et al., 2021). However, the required tacit knowledge to produce risky biological products is constantly decreasing due to the combination of SynBio with AI and automation.

Principally, forums such as DIY biology classes and Journal of Visualized Experiments (JoVE) videos can transfer the necessary skill and knowledge needed to use otherwise highly sophisticated techniques such as CRISPR during the development of an anthrax BW. Moreover, available kits can help to reduce knowledge and skill requirements during such CRISPR experiments given that the actor can select the appropriate kit and troubleshoot as needed (Paris, 2023). However, it is important to keep in mind that while some terrorist groups may want to genetically engineer *B. anthracis*; others may be satisfied with the most simple way to produce spores.

#### 3.4.2 Biological agent acquisition and agent properties

There are multiple ways to acquire the anthrax bioagent. Here, we more closely examine three, **t**he isolation from natural sources, the illegal procurement from authorized laboratories, and the modification of related organisms to an anthrax bioagent. Furthermore, we examine the possibilities to include antibiotic resistances.

##### 3.4.2.1 Isolation

Due to its well-known danger and for biosecurity reasons, *B. anthracis* is a regulated microorganism (CDC, 2023) by national and international conventions, that cannot be easily acquired from regular sources, such as culture repositories (Sharan et al., 2007). However, being widely distributed in sub-Saharan Africa, China, Kazakhstan, North-, South- and Central America, South- and East Europe, the Caribbean, the Middle East, and Australia (Carlson et al., 2019), one possibility to acquire various starins of *B. anthracis* would be to isolate this microorganism from natural reservoirs from the soil in the reported outbreak area or infested animal carcasses according to established and publicly available protocols (Böhm and Beyer, 2003). Undergraduate microbiology skills can be used to isolate *B. anthracis* from a natural contagious source. Most of the necessary production techniques are readily available in open-access journals and textbooks. With isolated starter culture, a terrorist could grow cultures with billions of spores in a 100-L vessel in less than a week under adequate biosafety precautions. The isolates should be positive in PCR assays for pXO1 and pXO2 probes. These probes are not subjected to security screening and are in general easily attainable, even for non-authorized institutions. Drying the slurry by freeze drying, for example, for weaponization is tricky, though not impossible (Green et al., 2007).

##### 3.4.2.2 Illegal procurement

Another possibility is the illicit acquisition from an authorized institution such as culture collections or research facilities working with dangerous pathogens. Although generally obligatory and stringent biosafety and biosecurity regulations are in place, there is always a possibility of sabotage or intentional misuse of available resources by staff members (as in the case of Amerithrax, 2001), or third parties. Therefore, the possibility of illegal procurement of *B. anthracis* from a research biosafety level (BSL-) 3 laboratory cannot be ruled out. Via relevant research publications and mapped containment laboratories (Bulletin of the Atomic Scientists, 2022) locating relevant BSL-3 laboratories has become an easy endeavor. In addition, there still exists the possibility of obtaining or thieving weaponized anthrax from a state’s offensive or defensive program, however less plausible, especially given that there is no knowledge about active offensive activities anywhere and that the number of states with appropriate/suitable defensive programs is limited (Sharan et al., 2007).

#### 3.4.3 Genetic engineering and synthetic biology

From the mid-1980s until 2003, the genome of *B. anthracis* was successfully sequenced (Read et al., 2003) and the two main megaplasmids carrying the main virulence factors pXO1 for the toxins factors edema factor (EF), protective antigen (PA), and lethal factor (LF) (110 MDa, 181 kb) (Green et al., 1985) as well as pXO2 for the capsule (60 MDa, 95 kb) encoding the three genes capB, capC, and capA (Makino et al., 1989; Okinaka et al., 1999) protecting from phagocytosis (Makino et al., 1989) were identified. Strains lacking either plasmid are either avirulent or significantly attenuated (Okinaka et al., 1999; Pilcher, 2003). In the upcoming years, knowledge about other pathogenicity factor genes increased, and “at the dawn of the 21st century, the scientific field of anthrax was perceived as a dead end” (Mondange et al., 2022).

However, as with the progress achieved with recombinant DNA in the 1970s and the rise of synthetic biology in the 2000s, the emergence of genome editing technologies, such as CRISPR in 2012, raised fears about that novel engineered strains of *B. anthracis* could become available for bioterrorism. Knowing the decoded *B. anthracis* genome with its more than 5,000 genes (Read et al., 2002; Pilcher, 2003), CRISPR made more precise editing of multiple genes simultaneously possible. In addition, genetic engineering for a fraction of the cost of predecessor technologies became feasible. Genetic modifications were now possible that used to be too demanding, laborious, or expensive in the past (Wang et al., 2019).

A more elaborate approach to obtain an anthrax-causing agent would be to modify a related microorganism, e.g., *B. cereus*, and convey all the characteristics of *B. anthracis*. One candidate for such a method could be *B. cereus* G9241, causing anthrax-like symptoms (Marston et al., 2016; Baldwin, 2020). As of 2022, this particular strain was available for purchase in limited amounts. Working with *B. cereus* requires only BSL-2 conditions, whereas with *B. anthracis* BSL-3 conditions have to be applied for intended aerosol production (Baldwin, 2020). Access restrictions become stricter with higher biosafety levels. Therefore, it is conceivable that such an organism may be misused by an actor aiming to “reproduce” anthrax by exploiting advances in biotechnology. Based on the information on the virulence factors given above, up to 14 genes may require editing depending on the original organism to be engineered. The chromosomal engineering could be conducted using the CRISPR/Cas kits which are readily available. Wang and coworkers (Wang et al., 2019) successfully edited the genomic DNA of *B. cereus* and *B. anthracis* using CRISPR/Cas9 and showed its efficacy for genome editing in the *B. cereus* group.

There are indeed certain genetic similarities between *B. anthracis* and *B. cereus* G9241, both possess two plasmids in the bacterial cytoplasm. One of the *B. cereus* plasmids, pBCX01, has a 99.63% homology with the pXO1 plasmid of *B. anthracis*, Ames strain. However, *B. cereus* G9241 lacks the pXO2 plasmid responsible for the formation of the polyglutamic acid capsule of *B. anthracis* (Hoffmaster et al., 2006). This physiological trait allows *B. anthracis* to evade immune response-mediated phagocytosis. The pXO2 plasmid encoding the polyglutamic acid capsule can in principle be synthesized *de novo* using oligonucleotides with overlapping sequences, an approach also used for the *de novo* synthesis of the polio virus (Cello et al., 2002). Oligos can be combined using assembly PCR. Afterward, the plasmid can be transferred into *B. cereus* G9421 by applying electroporation or other established methods (Ehling-Schulz et al., 2019).

In the context of the genetic engineering of *B. anthracis*, the incident around the Aum Shinrikyo cult might be of some interest. The characterization of a *B. anthracis* strain associated with the cult’s activities revealed no evidence of genetic modification (Keim et al., 2001). According to the results of the molecular genetic typing, the strain cultivated by Aum represented the Sterne vaccine strain, known to lack the pXO2 plasmid. On the other hand, Danzig and coworkers (Danzig et al., 2012) formulated a hypothesis that at some stage during their biological weapons program, one of the members of the Aum Shinrikyo cult attempted to transfer the genetic information for the capsule formation into the Sterne strain.

This would parallel the hypothetical scenario of *B. cereus* transformation discussed above. Both, *B. cereus* G9241 and *B. anthracis* Sterne do not possess the important virulence factor, the pXO2 plasmid for capsule formation. In principle, the plasmid could be transferred into the respective microorganism using the established tools of molecular biology. Without discussing the plausibility and likelihood of such an experiment performed by the Aum Shinrikyo cult, it can be insightful to compare the state of knowledge and technological advancement at that time and today.

Already 1988, at the time, when supposedly the biological weapons program of the Aum Shinrikyo cult was in progress, Makino and coworkers demonstrated the possibility of cloning the genetic region required for the encapsulation (Cap region) into *Escherichia coli* and *B. anthracis* (Cap-), which resulted in the encapsulation of both species in the presence of CO_2_ (Makino et al., 1989). A year later Stepanov and coworkers performed the transduction of pXO2 plasmid into different strains of *B. anthracis* (STI-1, Sterne, KM33, KM35) and reported that a “dramatic increase of virulence for white mice has been registered for *B. anthracis* strains having acquired the pXO2 plasmid replicon” (Stepanov et al., 1996). These experiments, among others, show that genetic manipulation of non-pathogenic *B. anthracis* or other microorganisms to convey the particular characteristics of lethal wild type anthrax was already feasible at the end of the 20th century.

The *de novo* synthesis of the respective genetic material for the encapsulation and the subsequent bacterial transformation would spare the necessity of acquiring such a regulated strain in the first place. In principle, a *de novo* synthesis of a Cap region could also be performed using the solid-state phosphoramidite method developed by Caruthers (Caruthers et al., 1987). The sequence of the 3.2 kbp long Cap region was published by Makino et al., in 1989 (Makino et al., 1989). By 1995, the longest DNA segment synthesized chemically and assembled from a large number of oligonucleotides was about 2.7 kbp (Stemmer et al., 1995).

No doubt, the possibilities for misuse of *B. anthracis* by terrorists have been expanded by the advances in science and technology and the huge amount of knowledge that has accumulated around *B. anthracis*. The (mis-)use of emerging technologies to genetically modify a harmless microorganism to produce anthrax toxins has been well documented in prokaryotes as well as eukaryotes. One of the candidates is *E. coli*, for which the expression of LF, EF, and PA of *B. anthracis* and their subsequent purification from this Gram-negative bacterium have been reported (Robertson and Leppla, 1986; Sharma et al., 1996; Kumar et al., 2001). Additionally, a Gram-positive spore-forming bacterium, *B. subtilis*, was used in one of the studies to produce recombinant LF (Gholami et al., 2021). Since *E. coli* and *B. subtilis* are broadly used in biochemistry and molecular, biology the barrier for an actor with malicious intent is rather low. In addition, there are other alternative systems for the expression of anthrax toxins. For instance, the yeast species *Pichia pastoris* was used for the expression of the *de novo* synthesized toxin of *Bacillus thuringensis* (Gurkan and Ellar, 2005). The advantage of using a eukaryotic organism is the post-translational modification of the toxins produced, which is lacking in prokaryotes.

It can be argued that the respective technical challenge of genome synthesis in the laboratory is lower nowadays due to the possibility of obtaining the corresponding oligos from commercial suppliers. The cost of ordering such sequences has steadily decreased over the years (Hoose et al., 2023), making the technology more accessible for use in biolabs, but also for misuse for malicious purposes. The beginning of the synthetic biology era marks the possibility of ordering *de novo* synthesized DNA from a commercial provider at desired concentrations and 100% purity. The orders of synthetic DNA are not subjected to mandatory screening. However, a vast majority of the companies working in this field have voluntarily introduced screening procedures based on the guidelines by the United States Department of Health and Human Services (HHS), e.g., Screening Framework Guidance for Providers of Synthetic Double-Stranded DNA (U.S. Department of Health and Human Services, 2010). The ordered sequences are matched with databases regarding pathogen and toxin sequences. Nevertheless, it is possible to evade such control mechanisms. One novel way around this is re-coding, which can be done either, less promisingly, by changing the codons, resulting in the same amino acid sequence. Or with encryption, whereby ultimately codons code for other amino acids (iGem Team, 2017). This would be a much more promising way to circumvent such controls, although much costlier, as all the gene sequences within the agent would have to be encrypted in the same way to allow functionality. So far, large-scale “rewiring” is not feasible, but research in this field advances rapidly. These aspects show that current screening algorithms need to be redesigned according to the developments in biotechnology.

But also the Engineering Biology research Consortium (EBRC Engineering Biology Research Consortium, 2022) itself lists some gaps, for instance, sequences of 200 bp or smaller are usually not cross-checked, since the results of oligo screenings might be ambiguous and expensive, compared to the cost of the DNA synthesis itself. Such shorter sequences could be assembled into longer sequences, thus creating a backdoor for abuse. Furthermore, the guidance refers only and explicitly to double-stranded DNA (dsDNA), not to single-stranded DNA or RNA. Both can be converted to dsDNA *in vitro*. Furthermore, the working group assumes that about 80% of the world’s DNA synthesis capacity is combined under these provisions, which leaves out a significant 20%. This is a major loophole in biosecurity, which can result in the synthesis and shipment of sequences of concern such as toxins*.*

Alternatively, the synthesis of oligos and short dsDNA can nowadays be also performed fast and at relatively low costs directly in the lab using state-of-the-art benchtop synthesizers. For more details see Carter et al. (Carter et al., 2023). Some of the devices synthesizing nucleic acids greater than 1,500 bp in length are subjected to export controls under the AG (2021) (The Australian Department of Foreign Affairs and Trade, 1958), but this does neither apply to all commercially available instruments nor national trade.

Even with all regulations in place, there remains the risk that an order may evade screening. For example, the sequence in question may be camouflaged by benign genes. Such a construct has a high probability to circumvent the screening procedure. The camouflaging genes can thereafter be removed through methods such as CRISPR/Cas9, and the deletions repaired via homology-directed repair (HDR), leaving a sequence encoding for a dangerous toxin or a virulence factor. This scenario may sound technically elaborate. However, proof of concept has been conducted by Puzis et al. (Puzis et al., 2020). No respective obfuscated DNA encoding a toxic peptide was detected by the screening algorithm, and the order was moved to production.

#### 3.4.4 Making bioagent antibiotic-resistant

It should be pointed out that *B. anthracis* is one of the most extensively studied microorganisms. Several mitigation measures and therapeutic strategies have been established over the years, which can be efficiently applied in the case of a potential outbreak (Supplementary Table S1). However, it is possible to introduce antibiotic resistance genes into the bacterial genome to circumvent these therapeutic strategies. Multidrug-resistant bacterial strains have been successfully engineered in the past (Dassanayake et al., 2021). In certain cases, multidrug resistance can lead to loss of virulence due to pleiotropic effects, as reported for the *Francisella tularensis* strain engineered to be resistant to multiple antibiotics as part of the USSR’s BW program (Leitenberg et al., 2012). There are also publications available, indicating that a *B. anthracis* strain (STI-1 vaccine strain) was engineered to resist several antibiotics (Stepanov et al., 1996). Therefore, the threat of a biological attack involving a multidrug-resistant lethal strain of *B. anthracis* cannot be understated.

#### 3.4.5 Technology acquisition and infrastructure

No doubt, the emerging, converging and enabling technologies led to a decrease in the requirement for sophisticated equipment thereby expanding the realm of feasibility and hence the BW paradigm. In addition, tools traditionally siloed in academic and government labs are increasingly becoming accessible to a wider audience (Dunlap and Pauwels, 2017; Sanz et al., 2022). Moreover, during the process of *B. anthracis* weapon development, less dangerous and easily available surrogate microorganisms can be used. Historically, the Japanise, the United States, the UK, and Iraq used *B. anthracis* surrogates in biological warfare test studies (Balmer, 2001; United Nations, 2007; Greenberg et al., 2010). More recently, research data were generated that could be exploited for *B. anthracis* BW development with a surrogate microorganism. For example, *B. thuringiensis* was effectively used as an appropriate model for *B. anthracis* in aerosol and re-aerosolization testing allowing environmental release without pathogenicity concerns (Tufts et al., 2014). In addition, using *B. thuringiensis* as a surrogate test organism opens new possibilities even for alternative non-regulated cultivation technologies such as solid-state fermentation (SSF) application as a new production system (Lima-Pérez et al., 2019).

Furthermore, significant progress in frugal science, collectively describing the attempt to create cheap, easy-to-use low cost and low electricity-requiring scientific equipment alongside emerging technologies made for anyone, anywhere could potentially be exploited to develop biological weapons (Tennenbaum et al., 2021).

#### 3.4.6 Biological agent amplification

Novel developments and contemporary lab practices make the cultivation and scale-up of *B. anthracis* more feasible. For large-scale cultivation, *B. anthracis* could be grown in submerged high-cell-density fermenters, as shown for the comparable *B. subtilis* (Grossman and Losick, 1988; Riesenberg and Guthke, 1999). Further upscaling would usually require large liquid-state fermenters which are subject to export controls under the AG (The Australia Group, 2021). To circumvent this restriction, a novel, unrestricted alternative SSF with polyurethane foams could be performed. This method was developed for *B. thuringensis* but is in principle applicable to *B. anthracis* (Lima-Pérez et al., 2019). SSF would therefore represent an unrestricted alternative method. All these aspects must be considered when discussing the imminent threat of an anthrax attack.

#### 3.4.7 Agent storage and transportation

There is also a large body of literature available on anthrax sporulation. It can be induced by a lack of nutrients in a freely available sporulation medium (Chen et al., 2020). Common histological stains (Moeller or Schaeffer-Fulton) are suitable to verify sporulation. Once obtained, the spores can be stored for extended timespans and disseminated by aerosolization. Not only can *B. anthracis* spores be stored for a long time, since they remain viable for decades, but they can also be easily transported in sealed containers and survive exposure to the Sun, air, rain, and violent dissemination methods. The spores are even so heat-resistant, they could be disseminated using explosives (Fetter, 1991).

#### 3.4.8 Stabilization and weapon-grade spore preparations

Historical indices show that several state and non-state actors transformed cultivated *B. anthracis* spores into a powder form as a part of anthrax weaponization. The purpose of this step is to improve the dissemination and aerosolization of this bioagent. It was reported that the United States (Matsumoto, 2003) and USSR (Zilinskas, 2014) produced dried anthrax spores in the scope of their BW programs. Also, the Aum Shinrikyo cult attempted to obtain anthrax in powder form (Danzig et al., 2012), while Iraq experimented with lypholization (Mondange et al., 2022). The best-known case of deploying anthrax as a powder is the 2001 anthrax attack (v. s. Amerithrax), where the spores were dried to the concentration of 2.0 × 10^12^ colony-forming units per Gram (USDOJ, 2010). Tufts and coworkers (Tufts et al., 2014) showed that *B. thuringensis* can be used as a surrogate to optimize the aerosolization of anthrax spores. Such a procedure requires sufficient technical expertise and special freeze- or spray-drying equipment. This category of dual-use equipment with a water evaporation capacity between 0.4 and 400 kg/h, and the ability to reach particle sizes below 10 µm or to sterilize or disinfect *in situ* is subjected to export controls under the EU regulations (European Union, 2021), implementing the AG-control lists (The Australia Group, 2021). Additional safety precautions such as a glovebox with negative pressure and protective clothing with an external oxygen supply are also highly recommended when working with aerosols containing pathogens. These items are also included in the AG control list of dual-use equipment and technologies. However, it can still be purchased within the country, second-hand, or crudely manufactured, albeit with technical challenges. Thus, the Aum Shinrikyo cult, although unsuccessfully employed a rudimentary self-made drier. Thus, aerosolization may be considered a bottleneck in the production of an anthrax BW. Probably the safest way to circumvent the regulations and controls in this regard would be to build the necessary equipment oneself, which would mean a significant increase in the necessary know-how in the field of engineering and infrastructure in the form of corresponding clandestine production facilities.

Another aspect in the context of anthrax weaponization is the encapsulation of the purified and dried spores. The encapsulation would impart additional stability and prevent aggregation (Matsumoto, 2003). It was suspected that the spores disseminated in the Amerithrax case were coated, based on the high silica level determined during the investigation. However, this hypothesis could not be verified experimentally. According to the results of the transmission electron microscopy, the silica was localized to the spore coat within the exosporium, and not on the surface. Therefore, it was concluded that silica was incorporated into the cells as a natural part of cell formation, rather than by a deliberate attempt to coat the spores. Despite this experimental evidence, the controversial debate on the spore coating in the Amerithrax case is still ongoing (Rosenberg, 2002; Bernstein, 2010; Epstein, 2010; National Research Council, 2011a).

From the perspective of this manuscript, it is of interest to evaluate the state of technology, which could in principle be applied to encapsulate the anthrax spores. As previously mentioned, *B. subtilis* can be used as a model organism for *B. anthracis*. Therefore, the encapsulation procedure described by Balkundi and coworkers (Balkundi et al., 2009) for *B. subtilis* has to be considered in the discussion on the advances in knowledge and technology, which might be misused for the weaponization of anthrax.

#### 3.4.9 Weapon deployment and agent dispersion

The method of BW deployment depends on the agent, its preparation, its stability, and the route of infection. In 1970, a World Health Organization (WHO) expert committee estimated that “an aircraft release of 50 kg of anthrax over an urban, developed population of 5 million would result in 250,000 casualties”. Medical resource limitation and capacity strain in such a scenario is enormous, ultimately requiring 13,000 hospital beds, 60-day of antibiotics for 125,000 patients leaving 95,000 dead. This would undoubtedly result in a quick and complete collapse of medical resources and civilian infrastructure. More recent estimations have confirmed the original WHO data (Congress, 1993). The CDC has developed an economic model that puts forward costs of $26.2 billion per 100,000 people exposed to an anthrax attack (Kaufmann et al., 1997). Fetter (Fetter, 1991) estimated that a missile armed with 30 kg of anthrax spores would affect an area of 6–80 square kilometers, delivering doses greater than 0.1 mg/min/m^3^ (the estimated ECt_50_ for anthrax) depending on the weather conditions and kill an estimated 20,000–80,000 people if a large, sparsely populated city was attacked. Alternatively, bioterrorists may disperse *B. anthracis* spores through aerosols using knapsack sprayers or a crop-spraying light aircraft to disseminate the biological agent (Durrant, 2002; Haas, 2002; Aduojo et al., 2022). The intimidating scenario of an attack with a UAV, commonly known as a drone, on a vulnerable target delivering weaponized anthrax can be considered increasingly realistic. In the Sverdlovsk incident, 1 g of wind driven anthrax spores killed sheep at a distance of up to 50 km (Durrant, 2002). Despite international regulations such as well-established import-export control regimes, up-to-date drones offer terrorists the convenience of anonymity and bypass traditional security measures (Pethő-Kiss, 2022).

### 3.5 Monitoring, medication, and mitigation

Fortunately, technological advances not only serve the development of bioweapons but also the development of mitigation strategies. Before an attack, surveillance through efficient bio-detection systems for environmental monitoring informing early warning systems, and preventative measures through vaccination as part of pre-exposure prophylaxis (PrEP) can be implemented.

To respond to an anthrax BW attack and mitigate its effects, multiple countermeasures including rapid detection, a comprehensive investigation and an effective response including post-exposure prophylaxis (PEP) must be considered. Designing rapid and reliable diagnostic systems by classical microbiology, immunoassays, and nucleic acid-based methods, including molecular forensics to identify *B. anthracis* or a related bacterial strain as the biological anthrax threat agent is a prerequisite to improve the response efforts (Blatny and Green, 2007) and to start with the therapeutic countermeasures. The treatment of infected people and animals with disease-specific interventions with antibiotics and antitoxins for PEP is necessary to decrease morbidity and mortality as much as possible (Honein and Hoffmaster, 2022; Rathish et al., 2022). In addition, panic and fear among the public must be managed by an interagency, intersectoral and international cooperation (Beeching et al., 2002) and proper public communication to minimize the disruptive impact of an anthrax attack (Cameron et al., 2019). Finally, containment and decontamination efforts after an anthrax event are necessary. In the following section, we discuss the most important of these aspects to draw conclusions considering biosecurity management.

#### 3.5.1 Detection

Shortly after the most recent bioterrorist Amerithrax attack and the complete genome sequencing of *B. anthracis* (Read et al., 2002), remarkable innovations and advances in the realm of anthrax detection and the newly initiated field of microbial forensics (Keim et al., 2001; Rasko et al., 2011) were made (Schmedes et al., 2019; Revill et al., 2022).

Conventionally, samples are assessed via microbiological growth analysis, Gram-, spore-, and capsule staining, microscopic analysis, hemolysis tests and phage susceptibility (Zasada, 2020). These methods require highly trained laboratory personnel, BSL-3 facilities, and practices. Novel detection methods are based on diverse targets, from detection based on DNA (Pal and Alocilja, 2010; Hao et al., 2011; Kaittanis et al., 2011; Chen et al., 2020), chemical reactions (Boyer et al., 2007; Duriez et al., 2009; Čapek et al., 2010; Kuklenyik et al., 2011), antibodies (De et al., 2002; Biagini et al., 2006; Campbell and Mutharasan, 2006; McGovern et al., 2007; Hao et al., 2009; Mwilu et al., 2009; Tang et al., 2009; Zahavy et al., 2010; Wang, 2013; Atabakhshi-Kashi et al., 2020), phages (Schuch et al., 2002; Fujinami et al., 2007), peptides (Acharya et al., 2007; Park et al., 2009), aptamers (Alibek and Handelman, 1999; Huan et al., 2009; Cella et al., 2010; Oh et al., 2011; Kim et al., 2013) or even DNA-peptide chimeras (Zhang and Appella, 2007; Kim et al., 2015; Wang D-B. et al., 2021). To date, a variety of detection methods for environmental or clinical anthrax samples emerged, each with distinct advantages over conventional culture and PCR-based detection. Xu and coworkers (Xu et al., 2023) developed a rapid (<40 min), easy-to-implement and accurate DNA endonuclease targeted CRISPR trans reporter (DETECTR)-based detection and identification method as a novel screening and diagnostic user-friendly portable devices for pathogenic *B. anthracis* (Xu et al., 2023). Overall, the speed, sensitivity and accuracy of modern detection methods have increased, potentially saving uncounted lives in case of an anthrax BW attack. Early detection is the prerequisite for adequate treatment and mitigation. In order to make a difference, these detection methods, must be widely available.

#### 3.5.2 Preparedness

In addition, the Amerithrax incident led to important investments in medical funding for biodefense. In the following decade, the United States, for example, spent 5.6 billion dollars on biodefense known as the Project BioShield Act 2004 (US Congress, 2004). While many wealthy countries followed the United States in an attempt to globally improve the capacity to face an emerging outbreak, although, with budgets that were and are orders of magnitudes lower (Mondange et al., 2022).

Currently, preparedness against the intentional use of *B. anthracis* relies on increased disease as well as environmental surveillance (US BioWatch program (National Research Council, 2011b)), laboratory capacity, information and system technology, education, and workforce training as well as clinical practice that integrates all accessible countermeasures such as new antimicrobials and advances in critical care (Blatny and Green, 2007; Scales and Horney, 2023). The armamentarium for PEP and treatment of anthrax involves numerous effective antimicrobials, including alternatives for resistant strains, antitoxins, and vaccines (Kaufer et al., 2020; Honein and Hoffmaster, 2022) that must be stockpiled in adequate quantities. Protocols to deal with anticipated *B. anthracis* scenarios are developed and tested in exercises.

Considering the preparedness towards anthrax attacks, there have been efforts to adopt strategies by various countries. Some examples are listed in the following: The US CDC (2015) published a clinical framework and medical countermeasure use during an anthrax mass-casualty incident. The focus was set on the allocation of scarce resources with different treatment plans depending on whether anthrax developed meningitis. The CDC recommends additional treatment with antitoxin in meningeal anthrax cases. The European Centre for Disease Prevention and Control (ECDC), however, only monitors cases in EU/European Economic Area countries and discusses them in their weekly Communicable Disease Threat Reports (ECDC. COMMUNICABLE, 2022). In their Annual Epidemiological Report for Anthrax, the ECDC also discusses the complementary administration of antitoxins, albeit additional benefits have been contested. The Department of Public Health of the Australian Government published a Public Health response plan for Anthrax (Australian Government Department of Health, 2012). Next to the description of clinical etiology and different treatment plans, this response plan entails measures for five different response codes (threat levels), for deliberate anthrax releases, defining the main actions and communication plans to be taken by the government and jurisdiction for each threat level and the key stakeholders.

#### 3.5.3 Pre- and postexposure treatments

Not surprisingly, the development of effective anthrax vaccines was spurred on by the potentially nefarious use of *B. anthracis* as a biological warfare agent. Already in 1953 and 1959 the USSR licensed their live spore vaccine for scarification and subcutaneous administration, respectively (Biselli et al., 2022). As new biochemistry methods in the 1950s and 1960s, paved the way for discovering and deciphering the capsule of *B. anthracis* (Smith et al., 1953; Thorne, 1960; Stanley et al., 1961), responsible for the toxin-mediated disease anthrax. In the 1970s, these breakthroughs and biodefense endeavors lead to the successful development and approval of a novel and enhanced cell-free human preparation of aluminum hydroxide gel adsorbed protective antigen, now known as anthrax vaccine adsorbed (AVA) formulation in the 1970s (Tournier et al., 2009; Tournier and Mohamadzadeh, 2010). At the same time, the WHO declares anthrax one of high-impact bioagents (World Health Organization, 1970).

Currently, the two primarily used culture filtrate vaccines, the Europe- and US-licensed Anthrax Vaccine Adsorbed (AVA; trade name BioThrax) and the United Kingdom-licensed vaccine, AVP, contain PA and variable quantities of LF and EF. Guidelines “recommend vaccination for people at risk, such as veterinarians, abattoir workers, those working with animal hides or furs, laboratory workers and the armed forces in areas with a high risk of exposure. In addition to PrEP the anthrax vaccine is also recommended for PEP, along with antibiotics” (ECDC, 2013). For individuals 18–65 years of age, various Anthrax vaccines are licensed or in development for PEP (Wolfe et al., 2020).

In addition, the antibiotics ciprofloxacin, penicillin, and doxycycline were approved by the Food and Drug Administration (FDA) for the treatment of anthrax and may be also useful in combination with other antibiotics for the treatment of inhalation anthrax (Inglesby et al., 2002). In *B. anthracis* antibiotic resistance to, e.g., amoxicillin, penicillin G, and/or cotrimoxazole has been documented. Although drug resistance mechanisms of *B. anthracis* have not yet been fully exploited, beta-lactamases against β-lactam antibiotics and efflux-pump mediating cross-resistance to fluoroquinolone antibiotics like ciprofloxacin in *B. anthracis* have been reported. Genetic modification of *B. anthracis* (to induce resistance to vaccines or antimicrobial drugs) has not yet been achieved by terrorists. Yet, the illicit Soviet program was successful. Hence, the introduction of safer and more efficient chemotherapeutic options are required (Dassanayake et al., 2021).

Moreover, antibiotics are effective against bacteremia caused by antibiotic-susceptible strains of anthrax but not against the toxemia that drives pathogenesis. In fact, the quantities of secreted anthrax toxins in some cases lead to death despite efficient antibiotics administration. The discovery of the biochemical structure of LF and EF (Pannifer et al., 2001), of the cellular receptors of PA (Bradley et al., 2001), and description of the precise effects of LF and EF on the cell biology (Moayeri et al., 2015), therefore, were important scientific achievements in the toxin field. In 2009, the first monoclonal antibody targeting PA was finally authorized by the FDA (Migone et al., 2015). Nowadays, three anthrax antitoxins have been approved by the FDA and stockpiled by the United States: two monoclonal antibodies (raxibacumab and obiltoxaximab “Anthim”), and the human polyclonal purified IgG from vaccinated humans (intravenous anthrax immune globulin AIG-IV, also referred as Anthrasil) (Huang et al., 2015; Avril et al., 2022), regardless of uncertainties associated with the clinical effectiveness of antibodies. Hence, Anthim and Anthrasil can be administered solo or in combination with antibiotics for a more effective anthrax therapy. According to the CDC, the administration of both antibiotics and antibodies is recommended, regardless of recent studies doubting the efficiency of antibodies (Tournier et al., 2019; Avril et al., 2022).

#### 3.5.4 Decontamination

Generally, remediation following a *B. anthracis* BW attack requires decontamination, confirmatory sampling, and testing. The decontamination strategy should include the decontamination of surfaces and affected areas (space), as well as the proper disposal of any decontamination wastewater (Urban-Sorensen, 2018). In the aftermath of the Amerithrax attack, both private and government facilities were affected, and their cleaning up was an unexpected challenge. The decontamination work was not only high-profile but also very time-consuming and expensive. A complete renovation of all facilities required over 3 years and cost about $320 million (Urban-Sorensen, 2018). Meanwhile, specific advances in nanotechnology and material sciences led to the improvement of decontamination and decontamination capabilities even against spore-forming bacilli. For decontamination applications against *B. anthracis* with up to 100% efficacy after 10–15 min, Ginghina (Ginghina et al., 2022) demonstrated the antimicrobial activity of organic solutions enriched with ZnO, TiO_2_, and zeolite nanoparticles. Another effective strategy is to incorporate different semiconductors to enhance their bactericidal synergistic effects for water disinfection. A maximum antimicrobial activity against *B. subtilis* was shown by CuWO_4_/CuS CuS nanopowder (Dong et al., 2022). Moreover, Nakonieczna (Nakonieczna et al., 2022) recently identified three new siphophages that can specifically infect and lyse siphophages that can specifically infect and lyse *B. anthracis* and have applications as decontaminants or disinfectants (of skin, surface, or clothes).

### 3.6 Threat evaluation

To assess vulnerability, a threat evaluation is necessary. The prevention of unwanted events from occurring and/or protection, the ability to react during an event, and the ability to mitigate its subsequent impact are the goals of any good security measure (Tennenbaum et al., 2021). Given i) the potentially very high death toll due to an anthrax attack and the societal and economic disruption in the aftermath of an attack, ii) the demonstrated relative feasibility of acquisition, mass production and weaponization of anthrax, partly by circumventing existing regulations and governance measures, iii) the existence of disseminating technology, iv) and the difficulty of effective emergency response including the sufficient stockpiled antibiotics, antitoxins and vaccines, it is crucial to strengthen preparedness, prevention, and mitigation measures.

For an evaluation of the posed threat considering anthrax and BW research, we identified many indicators in different fields with a dual-use potential relevant to anthrax BW development (Supplementary Table S1). As can be seen from the prominent examples of Amerithrax and Aum Shinrikyo, foremost terrorists try to use anthrax as a bioweapon. Based on identified indicators in different relevant fields with a dual-use potential relevant to an anthrax BW development, our analysis clearly showed the rapid speed at which scientific achievements in the field of SynBio and other emerging and converging technologies are taking place (Supplementary Table S1), thereby paving the way for potential novel and high consequence BW threats.

On the one hand, key technologies that could support efforts to engineer a novel anthrax BW were identified. The indicators most relevant to our pathway exploration were scientific achievements, advancements or discoveries that could be exploited to increase the BW threat potential or to circumvent biosecurity measures aiming at preventing BW proliferation and development. The essential anthrax virulence factors are located on just two plasmids, allowing their transfer from one bacterium to another (Makino et al., 1988; Stepanov et al., 1996), as was already proven in *E. coli* (Robertson and Leppla, 1986; Sharma et al., 1996; Kumar et al., 2001). Together with the existence of phylogenetic closely related and less dangerous surrogate species such as *B. subtilis* (Zhang et al., 2019; Gholami et al., 2021), *B. thuringiensis* (Lima-Pérez et al., 2019) and *B. cereus* (Manoharan et al., 2023), this presents a major possibility for exploitation and potential for safer, low-cost and undercover BW research. Furthermore, the advent of CRISPR made genetic modification easier, quicker and cheaper, while toxin sequences (GenBank, 1995) and protocols for the isolation of *B. anthracis* from contaminated soil (Chikerema et al., 2012), high-cell density cultivation (Zhang et al., 2019), sporulation (Chen et al., 2020) and other techniques necessary for BW development are readily available on the internet. To top this all off, there are commercial suppliers for mail-order nucleic acid sequences, which are not bound to perform mandatory screenings. And even if they were mandated to perform screenings, there would be ways to circumvent them (Atkins and Baranov, 2010; Engineering Biology Research Consortium Security Working Group, 2022). In addition, potential hazardous modifications include antibiotic resistance, or heightened pathogenesis, an easier disseminatable and enhanced aerosolization of the BW agent. Using recombinant DNA technology even a non-regulated *B. cereus* strain could be turned into an anthrax BW that could escape the established bio-detection and biomedical defense strategies. In addition, many of the identified technological advances are explicitly designed to decrease the technical expertise required to produce sufficient quantities of biological agents for a bioterrorist group with nefarious intentions. Importantly, this can fundamentally change signatures used to identify suspicious and illegal activity by intelligence analysts and law enforcement professionals.

On the other hand, technological advances also led to an improvement in the realm of counterproliferation, detection, and development of medical countermeasures, thereby raising the PrEP and PEP targeted to counter and reduce threats. The chemotherapeutic management of anthrax has become challenging due to the global emergence of antibiotic-resistant strains. However, a plethora of bioactive phytochemicals with an antibiotic-potentiating ability and reversing antibiotic resistance in *B. anthracis* have been identified (Dassanayake et al., 2021). In addition, the discovery of potent new antibiotics such as anthracimycin with a novel mechanism of action (inhibiting DNA/RNA synthesis) and low toxicity to human cells represents a major advance in the field of antibiotics against *B. anthracis* helping to counter existing or future antibiotic resistance problems (Tian et al., 2022). Moreover, three new siphophages that can specifically infect and lyse *B. anthracis* were recently isolated. Beside finding potential use in *B. anthracis* identification and detection assays, the siphophages, after removing the genomic modules essential for lysogeny, can be applied to treat human or animal anthrax (likewise their endolysins), or as surface or skin decontaminants or disinfectants (Nakonieczna et al., 2022).

However, in a large-scale bioterrorist anthrax incident, it is especially critical to meet the need for anthrax vaccines and antitoxins (Dassanayake et al., 2021; Hesse et al., 2022). Representing a bottleneck for mitigation in case of an anthrax attack, vaccines and antimicrobics have to be stockpiled for rapid mobilization and distributed to large numbers of people (Beeching et al., 2002).

The combination of these findings draws a sobering picture implying a low-entry and potentially high-threat situation. However, these advancements simultaneously also offer new opportunities to address them.

### 3.7 Biosecurity measures

All of the BWC Review Conferences since the 1990s have failed to take decisions that would help shaping biosecurity measures on the international as well as national level or at public or private biotechnology facilities. The here discussed measures were hence developed through other mechanisms. The current technological possibilities to weaponize *B. anthracis* discussed here highlight several aspects which are of importance in the context of risks posed by dual-use research (see Introduction).

• Misuse of results published openly in literature (e.g., creating multidrug-resistant strains of *B. anthracis*, expressing anthrax toxins in other microorganisms).
• Conducting gain-of-function experiments (GOF) for malicious intent (e.g., genetic engineering of *B. cereus* to convey the characteristics of wild type *B. anthracis*).
• Exploiting recent and emerging advances in technology for malicious purposes (e.g., UAVs, modern aerosolizers or ordering DNA sequences encoding for *B. anthracis* toxins or virulence factors from a commercial provider).

These aspects present just a fraction of the dual-use research problems in science and industry that need to be addressed by designing and applying comprehensive ethical and legal frameworks. However, in the scientific community, there is still little awareness of the fact that research results and technological achievements can be misused by certain actors for hostile purposes.

While such efforts have no bearing on terrorists, many initiatives for the scientific codes of conduct have been recently developed to minimize biosafety and biosecurity risks. They include the Recommendations for Handling Security-Relevant Research drafted by the German Research Foundation and the National Academy of Sciences Leopoldina (Deutsche Forschungsgemeinschaft and Deutsche Akademie der Naturforscher Leopoldina, 2014) and the Global guidance framework for the responsible use of the life sciences (World Health Organization, 2022). Another prominent example is the Tianjin Biosecurity Guidelines for Codes of Conduct for Scientists (Wang L. et al., 2021), which were, however, not endorsed by the Ninth Review Conference of the BWC in December 2022. All these promising ethical tools urge that *“[m]easures should be taken to prevent the misuse and negative impacts of biological products, data, expertise, or equipment”* (WHO, 2022)*.* This also implies a responsible publication of results in scientific literature. Transparency and knowledge sharing are undoubtedly important driving forces in high-quality research. Nevertheless, as we illustrate in this manuscript, some published data might pose a great risk of misuse. Examples include investigating the sporulation process of *B. anthracis* while using other related organisms such as *B. thuringensis* or *B. subtilis*. A more striking example is the publication on the genetically engineered multidrug-resistant *B. anthracis* strain. The data on this experiment was published in the 90s, prior to the “Fink Report” (National Research Council, 2004). However, the open-access body of literature on some research areas of concern outlined in this report continues to grow, as demonstrated by the recent pre-print publication on the chimeric recombinant Sars-CoV-2 (Chen et al., 2023). This clearly indicates the need for a more sophisticated review mechanism for scientific journals and addresses the issue of making publications openly available in preprint repositories before they undergo a review process.

In addition to the research results published in scientific journals and preprint repositories, other sources of scientific data can be potentially subjected to misuse. This for instance applies to open-access genomic and proteomic databases. The National Institute of Health GenBank contains complete genome sequences of various microorganisms and viruses with varying data quality, including *B. anthracis* Ames, Hepatitis B virus, Influenza A (segments 1–8), *Yersinia pestis*, *etc.* The fact that this information can be misused for the *de novo* synthesis of some of the genes, or even for the recreation of an entire organism (see cases of poliovirus and horsepox virus) cannot be denied. One of the possible mitigation strategies could be more restricted access to the data banks through a licensing policy. A preregistration of research for biosecurity risk assessment earlier in the research process and eventually access-controlled repositories or application programming interfaces after completion of research has already been demanded (Smith and Sandbrink, 2022). These steps, however, require scrutiny and a solid proof-of-principle in order not to create a serious bureaucratic obstacle to peaceful science, while making a minimal contribution to biosecurity (due to the existence of possible backdoors for misuse, *etc.*).

Another important aspect is the highly controversial GOF research area. It has sparked numerous debates in the past (Kaiser, 2022). A more in-depth analysis of the matter is beyond the scope of this manuscript. Nevertheless, it is of relevance to our discussion on weaponizing anthrax. Modifying *B. cereus* in such a way that it would express anthrax toxins and important virulence factors would meet the definition of “enhancing” an agent. Recently, the United States National Science Advisory Board for Biosecurity approved a report on amending the review process of GOF experiments in the United States and abroad (in cooperation with United States research institutions) (Reardon, 2023). According to it, all studies should be subjected to a meticulous review, if they could be “reasonably anticipated” to make a pathogen more dangerous. The guidelines are still vague and will undergo several modifications before being finalized. Even if applied in a consequent manner, there remains the question, of which impact, if any, these regulations would have on other GOF experiments conducted worldwide. The current frameworks do not appear to be effective in limiting dissemination of research that could enhance the dangers posed by a future use of *B. anthracis*. This matter requires an open dialogue on a multinational level.

In the broad discussion about the ethical obligations of the scientific community, little attention is paid to the responsibility of other stakeholders, such as the private sector. As the bioeconomy grows, privately funded life science research with dual-use potential is on the rise (Epstein, 2023). A major drawback there is, for example, the lack of standardized guidelines and customer screening mechanisms to reduce the risk of misuse of advanced medical and biotechnological applications and devices supplied. Thus, companies providing dual-use equipment should implement reliable mechanisms to check their customers’ and cooperation partners’ backgrounds. Full-scale training in biosecurity, international norms, and ethical issues should be provided in both non-commercial research institutions and industrial biotechnology facilities.

In the pathway analysis, we focus on the case of commercial providers of synthetic DNA and indicate that the guidelines proposed by HHS are, for now, still voluntary and bear some limitations concerning biosecurity. A unified easy-applicable and low-cost mechanism for screening both the customer and the ordered DNA is of utmost importance. Several proposals for such mechanisms have been developed over the years, including the implementation of a harmonized database for the “sequences of concern” (sequences encoding for toxins and virulence factors, excluding other housekeeping genes of an organism, to make the screening less ambiguous and time-consuming). In particular, the Nuclear Threat Initiative (NTI) is making laudable progress in establishing an international common mechanism for DNA synthesis screening based on the above criteria, which should be operational soon (The Nuclear Threat Initiative, 2023). However, all guidelines will have limited effectiveness unless they are declared mandatory worldwide. To the best of our knowledge, only the California government has taken an initial step in the direction of strengthening SynBio-security, requiring scientists to develop systemwide guidance for purchasing gene synthesis equipment and products from “providers who prevent the misuse of synthetic genes” (LegiScan. California Assembly Bill, 1963, 2022). However, more stringent (international) legislation, including also legally binding regulations for the industry is still needed.

In summary, we support the overarching proposals made by the above-mentioned ethical frameworks and would like to emphasize the following.

• Comprehensive training for raising awareness in the scientific community should become a mandatory part of any curriculum at academic institutions; it should also be included in annual training of the scientific staff at non-profit research and industrial facilities;
• Biosecurity relevant research should be registered for biosecurity risk assessment;
• A background check should be considered for scientific staff members working on biosecurity-relevant research;
• Ethical and policy recommendation committees should be convened at institutions to monitor and evaluate the proposed research projects and to guide them during their progress;
• Access-controlled repositories or application programming interfaces for open science should be implied while access to genome/proteome databanks should be better supervised (through e.g., licensing);
• A broader and stricter mechanism for reviewing the submitted manuscripts and research proposals should be put in place by the funding agencies and the scientific journals (since Some journals are published by for-profit publishers and may not be as rigorous);
• Screening procedures of the synthetic DNA ordered from commercial suppliers should be unified and mandatory;
• Strict and continuous documentation, monitoring, and accountability of laboratory storage, and utilization of pathogens, toxins, biosecurity-relevant substances, and sequences
• BWC states parties using the intersessional process towards the 10th review conference in 2027 to develop sufficient multilateral activities, such as the installation of a Science and Technology advisory board, a verification system shaped to the progress in the field, adopting a significant code of conduct for the life sciences, adopting and strengthening the system of Confidence Building Measures, *etc.* Such a verification system should consider the fundament of the draft BWC verification protocol of 2001, might be conceived similarly to that of the Chemical Weapons Convention (CWC) but should but must be extended due to the different technology and stakeholder environment (i.e., mail order, DIY labs, cloud labs, *etc.*). Furthermore, transparency toward DURC and GOF research should be included in a monitoring and verification system.

These steps should be openly discussed with and accepted by the scientific community and other stakeholders. Otherwise, they might result in a patchwork-like loose implementation that hampers scientific progress, while making little contribution to biosecurity.

## 4 Conclusion

Biological weapons do not only pose a threat through state-sponsored programs but also in bioterrorism and bio-crime incidents. Globally, huge efforts are being made to strengthen the norm against biological weapons and to implement effective biological arms control strategies. These include binding laws prohibiting the development, production, stockpiling and use of biological weapons. In fact, many states have been historically financially and technically capable of engaging in clandestine biological warfare programs including *B. anthracis* BWs. Two decades have past since the Amerithrax attacks without any further comparable incidents. Throughout the entire time period, however, the narrative developed that the threat is constantly growing. This article, investigates the anthrax BW threat by terrorists in the here and now.

In our pathway analysis, we analyzed three different acquisition pathways, isolation, illegal procurement and various routes of genetic engineering. These pathways vary greatly in labor intensity, necessary secrecy levels and biosafety requirements, as well as costs. While it may be possible to steal already weaponized spores from legitimate facilities, isolation and genetic engineering requires much more work. Similarly, biosafety requirements may widely differ depending mostly on the level of readiness of the illegally procured bioagent. Hence, costs may scale where the highest costs would be expected for the genetic engineering pathway. We would refrain from estimating explicit cost ranges, since they mostly depend on the number of people involved and their monetary compensation, as well as the necessary infrastructure which may vary situationally and geographically. Also, it is hardly possible to determine a number of person labor hours since this kind of work, especially genetic engineering, is more breakthrough-dependent. On the other hand, secrecy would likely be least sensitive in the isolation pathway and most sensitive in the illegal procurement pathway while depending on the number of people involved and the timespan of production.

Since terrorists do not comply with the existing strong global norm that rejects development of such weapons, raising preparedness and implementing preventive measures are the only effective strategies. Despite improvements in treatment, inhalation anthrax remains a deadly infection. Prevention, therefore, foremost implies promt detection, timely diagnosis, and immediate treatment of disease, as well as providing sufficient intensive-care facilities and effective antimicrobials, to significantly reduce the morbidity and mortality of inhalational anthrax. In fact, achievements have been made in all these areas including the discovery of new and effective antibiotics and bacteriophages as well as improvements in vaccination strategies and the invention of rapid portable detection devices and sensors. However, the question remains, whether they are capable of compensating the existing elevated threat level of a potential *B. anthracis* BW development and deployment by terrorists identified through our aforementioned pathway analysis. In addition, the past failures of terrorists in pursuing anthrax BWs should not be a source of consolation, but rather a warning of an activity that, if persistently pursued with the aid of advances in emerging and converging sciences, could eventually lead to success.

## Figures and Tables

**Table:** 

**3D**	Three dimensional
**AG**	Australia Group
**AI**	Artificial Intelligence
**AIG-IV**	Intrevenous Anthrax immune globulin
**AVA**	Anthrax vaccine adsorbed
**AVP**	Anthrax vacinne precipitated bp Base pairs
**BSAT**	Biological Select Agents and Toxin
**BSL**	Biosafety level
**BW**	Biological weapon(s)
**BWC**	Biological Weapons Convention
**CDC**	Centers for Disease Control and Prevention
**CRISPR**	Clustered regularly interspaced short palindromic repeats
**CRISPR/cas9**	Clustered regularly interspaced short palindromic repeats/CRISPR-associated protein 9
**DETECTR**	DNA endonuclease targeted CRISPR trans reporter
**DIY**	Do-It-Yourself
**DIY-Bio**	Do-It-Yourself Biology
**DNA**	Desoxyribonucleic acid
**dsDNA**	Double-stranded desoxyribonucleic acid
**DURC**	Dual-Use research of concern
**ECDC**	European Centre for Disease Prevention and Control
**EF**	Edema factor
**EU**	European Union
**FDA**	Food and Drug Administration
**GOF**	Gain-of-function
**HCD**	High cell density
**HDR**	Homology-directed repair
**HHS**	United States Department of Health and Human Services
**JoVE**	Journal of Visualized Experiments
**LF**	Lethal factor
**MERCs**	Multi export regime controls
**mRNA**	Messenger ribonucleic acid
**NTI**	Nuclear Threat Initiative
**PA**	Protective antigen
**PCR**	Polymerase Chain Reaction
**PEP**	Post-exposure prophylaxis
**PrEP**	Pre-exposure prophylaxis
**RNA**	Ribonucleic acid
**rRNA**	Ribosomal ribonucleic acid
**SSF**	Solid-state fermentation
**SynBio**	Synthetic Biology
**UAV**	Unmanned aerial vehicles
**UK**	United Kingdom
**UNSC**	United Nations Security Council
**US**	United States of America
**USSR**	Union of Soviet Socialist Republics
**WHO**	Worls Health Organization

## Data Availability

The original contributions presented in the study are included in the article/Supplementary Material, further inquiries can be directed to the corresponding author.

## References

[B1] Acevedo M. M. Carroll L. M. Mukherjee M. Mills E. Xiaoli L. Dudley E. G. (2019). *Bacillus clarus* sp. nov. is a new *Bacillus cereus* group species isolated from soil. BioRxiv. 10.1101/508077 PMC7643830 33148822

[B2] Acharya G. Doorneweerd D. D. Chang C-L. Henne W. A. Low P. S. Savran C. A. (2007). Label-free optical detection of anthrax-causing spores. J. Am. Chem. Soc. 129, 732–733. 10.1021/ja0656649 17243788

[B3] Aduojo E. E. Amina S. B. Kemi O. Jabir A. (2022). Bioterrorism and biodefence: Biotechnology and security implications for Nigeria. Am. J Bioterror Biosecur Biodefens 5, 1–5. Available at: https://www.researchgate.net/profile/Amina-Bature/publication/360121455_Bioterrorism_and_Biodefence_Biotechnology_and_Security_Implications_for_Nigeria/links/62632d6ebca601538b60bbd1/Bioterrorism-and-Biodefence-Biotechnology-and-Security-Implications-for-Nigeria.pdf.

[B4] Ainscough M. J. (2002). Next generation bioweapons: The technology of genetic engineering applied to biowarfare and bioterrorism.

[B5] Alibek K. Handelman S. (1999). Biohazard: The chilling true story of the largest covert biological weapons program in the world -- told from the inside by the man who ran it; biological weapons: Limiting the threat. New York, New York: Random House.10036

[B6] Amanatidou E. Butter M. Carabias V. Konnola T. Leis M. Saritas O. (2012). On concepts and methods in horizon scanning: Lessons from initiating policy dialogues on emerging issues. Sci. Public Policy 39, 208–221. 10.1093/scipol/scs017

[B7] American Society for Microbiology (2017). Identification of Bacillus cereus biovar anthracis. Available at: https://asm.org/Guideline/Identification-of-Bacillus-cereus-biovar-anthracis.

[B8] Argôlo-Filho R. C. Loguercio L. L. (2013). *Bacillus thuringiensis* is an environmental pathogen and host-specificity has developed as an adaptation to human-generated ecological niches. Insects 5, 62–91. 10.3390/insects5010062 26462580 PMC4592628

[B9] Atabakhshi-Kashi M. Geranpayehvaghei M. Wang Y. Akhbariyoon H. Taleb M. Zhang Y. (2020). Recent advances of nanocarriers for effective delivery of therapeutic peptides. Precis. Nanomedicine 3. 10.33218/001c.13444

[B10] Atkins J. F. Baranov P. V. (2010). The distinction between recoding and codon reassignment. Genetics 185, 1535–1536. 10.1534/genetics.110.119016 20713743 PMC2921827

[B11] Australian Government Department of Health (2012). Anthrax: Public health response plan for Australia. Guidelines for preparedness, response and management following the deliberate release of Bacillus anthracis.

[B12] Avril A. Tournier J-N. Paucod J-C. Fournes B. Thullier P. Pelat T. (2022). Antibodies against anthrax toxins: A long way from benchlab to the bedside. Toxins (Basel) 14, 172. 10.3390/toxins14030172 35324669 PMC8955606

[B13] Bakhtin P. Saritas O. Chulok A. Kuzminov I. Timofeev A. (2017). Trend monitoring for linking science and strategy. Scientometrics 111, 2059–2075. 10.1007/s11192-017-2347-5

[B14] Baldwin V. M. (2020). You can't *B. cereus* - a review of *Bacillus cereus* strains that cause anthrax-like disease. Front. Microbiol. 11, 1731. 10.3389/fmicb.2020.01731 32973690 PMC7468541

[B15] Balkundi S. S. Veerabadran N. G. Eby D. M. Johnson G. R. Lvov Y. M. (2009). Encapsulation of bacterial spores in nanoorganized polyelectrolyte shells. Langmuir 25, 14011–14016. 10.1021/la900971h 19469562

[B16] Balmer B. (2001). Britain and biological warfare. London: Palgrave Macmillan UK.

[B17] Bazinet A. L. (2017). Pan-genome and phylogeny of *Bacillus cereus sensu lato* . BMC Evol. Biol. 17, 176. 10.1186/s12862-017-1020-1 28768476 PMC5541404

[B210] Trump B. D. Florin M-V. Perkins E. Linkov I. (Editors) (2021a). Emerging threats of synthetic biology and biotechnology. Addressing security and resilience issues. 1st ed. (Dordrecht: Springer Netherlands; Imprint Springer). 36121971

[B18] Beeching N. J. DavidABDance Miller A. R. Spencer R. C. (2002). Biological warfare and bioterrorism. BMJ Clin. Rev. 324, 336–339. 10.1136/bmj.324.7333.336 PMC1122269 11834562

[B19] Beedham R. J. Davies C. H. (2020). The UK biological-warfare program: Dual-use contributions to the field of aerobiology. Nonproliferation Rev. 27, 309–322. 10.1080/10736700.2020.1823621

[B20] Bernstein B. J. (1987). The birth of the U.S. Biological-warfare program. Sci. Am. 265, 116–121. 10.1038/scientificamerican0687-116 3296173

[B21] Bernstein R. (2010). Haste leaves anthrax case unconcluded. The New York times. Available at: https://www.nytimes.com/2010/02/25/us/25iht-letter.html (Accessed March 31, 2023).

[B22] Biagini R. E. Sammons D. L. Smith J. P. MacKenzie B. A. Striley C. A. F. Snawder J. E. (2006). Rapid, sensitive, and specific lateral-flow immunochromatographic device to measure anti-anthrax protective antigen immunoglobulin g in serum and whole blood. Clin. Vaccine Immunol. 13, 541–546. 10.1128/CVI.13.5.541-546.2006 16682473 PMC1459649

[B23] Biselli R. Nisini R. Lista F. Autore A. Lastilla M. Lorenzo G. de (2022). A historical review of military medical strategies for fighting infectious diseases: From battlefields to global health. Biomedicines 10, 2050. 10.3390/biomedicines10082050 36009598 PMC9405556

[B24] Blatny J. M. (2007). “Detecting and responding to bioterrorism,” in Risk assessment and risk communication strategies in bioterrorism preparedness. Editor Green M. (Dordrecht: Springer).

[B25] Böhm R. Beyer W. (2003). Bioterroristische Anschläge mit *Bacillus anthracis* . Bundesgesundheitsblatt - Gesundheitsforsch. - Gesundheitsschutz. 46, 956–964. 10.1007/s00103-003-0715-1

[B26] Boyer A. E. Quinn C. P. Woolfitt A. R. Pirkle J. L. McWilliams L. G. Stamey K. L. (2007). Detection and quantification of anthrax lethal factor in serum by mass spectrometry. Anal. Chem. 79, 8463–8470. 10.1021/ac701741s 17929949

[B27] Bracamonte A. G. (2023). Current advances in nanotechnology for the next generation of sequencing (NGS). Biosens. (Basel). 13, 260. 10.3390/bios13020260 PMC9954403 36832027

[B28] Bradley K. A. Mogridge J. Mourez M. Collier R. J. Young J. A. (2001). Identification of the cellular receptor for anthrax toxin. Nature 414, 225–229. 10.1038/n35101999 11700562

[B29] Brockmann K. Bauer S. Boulanin V. (2019). BIO plus X: Arms control and the convergence of biology and emerging technologies.

[B30] Bulletin of the Atomic Scientists (2022). Global Biolabs. Tracking maximum containment labs around the world. Available at: https://thebulletin.org/global-biolabs/ (Accessed March 31, 2023).

[B31] Cameron E. Katz R. Konyndyk J. Nalabandian M. (2019). A spreading plague: Lessons and recommendations for responding to a deliberate biological event.

[B32] Campbell G. A. Mutharasan R. (2006). Piezoelectric-excited millimeter-sized cantilever (PEMC) sensors detect *Bacillus anthracis* at 300 spores/mL. Biosens. Bioelectron. 21, 1684–1692. 10.1016/j.bios.2005.08.001 16169715

[B33] Čapek P. Kirkconnell K. S. Dickerson T. J. (2010). A bacteriophage-based platform for rapid trace detection of proteases. J. Am. Chem. Soc. 132, 13126–13128. 10.1021/ja104572f 20812737 PMC2949351

[B34] Carlson C. J. Kracalik I. T. Ross N. Alexander K. A. Hugh-Jones M. E. Fegan M. (2019). The global distribution of *Bacillus anthracis* and associated anthrax risk to humans, livestock, and wildlife. Nat. Microbiol. 4, 1337–1343. 10.1038/s41564-019-0435-4 31086311

[B35] Carter S. R. Yassif J. M. Isaac C. R. I. (2023). Benchtop DNA: Synthesis devices: Capabilities, biosecurity implications, and governance.

[B36] Carus W. S. (2017). A century of biological-weapons programs (1915–2015): Reviewing the evidence. Nonproliferation Rev. 24, 129–153. 10.1080/10736700.2017.1385765

[B37] Carus W. S. (2001). Bioterrorism and biocrimes: The illicit use of biological agents since 1900. Washington, DC: Center for Counterproliferation Research National Defense University, 1–209.

[B38] Caruthers M. H. Barone A. D. Beaucage S. L. Dodds D. R. Fisher E. F. McBride L. J. (1987). Chemical synthesis of deoxyoligonucleotides by the phosphoramidite method. Methods Enzymol. 154, 287–313. 10.1016/0076-6879(87)54081-2 3431460

[B39] Caudle L. C. III Sidell F. R. Takafuji E. T. Franz D. R. The biological warfare threat (1997). Medical aspects of chemical, 451–466.

[B40] CDC (2023). Select agents and toxins list. HHS and USDA select agents and toxins 7CFR Part 331. 9 CFR Part 121, and 42 CFR Part 73. 2023. Available at: https://www.selectagents.gov/sat/list.htm.

[B41] Cella L. N. Sanchez P. Zhong W. Myung N. V. Chen W. Mulchandani A. (2010). Nano aptasensor for protective antigen toxin of anthrax. Anal. Chem. 82, 2042–2047. 10.1021/ac902791q 20136122 PMC2930939

[B42] Cello J. Paul A. V. Wimmer E. (2002). Chemical synthesis of poliovirus cDNA: Generation of infectious virus in the absence of natural template. Science 297, 1016–1018. 10.1126/science.1072266 12114528

[B43] Centers for Disease Control and Prevention (2023). History of anthrax. Available at: https://www.cdc.gov/anthrax/basics/anthrax-history.html (Accessed January 17, 2023).

[B44] Chen D-Y. Kenney D. Chin C-V. Tavares A. H. Khan N. Conway H. L. (2023). Role of spike in the pathogenic and antigenic behavior of SARS-CoV-2 BA.1 Omicron. BioRxiv, 2022.10.13.512134. 10.1101/2022.10.13.512134

[B45] Chen M. Lyu Y. Feng E. Zhu L. Pan C. Wang D. (2020). SpoVG is necessary for sporulation in *Bacillus anthracis* . Microorganisms 8, 548. 10.3390/microorganisms8040548 32290166 PMC7232415

[B46] Chikerema S. M. Pfukenyi D. M. Hang'ombe B. M. L'Abee-Lund T. M. Matope G. (2012). Isolation of *Bacillus anthracis* from soil in selected high-risk areas of Zimbabwe. J. Appl. Microbiol. 113, 1389–1395. 10.1111/jam.12006 22984812

[B47] Cole L. A. (2010). “Anthrax as a weapon of war and terrorism,” in Bacillus anthracis and Anthrax. Editor Bergman N. H. (Wiley), 295–308.

[B48] Congress U. S. (1993). Office of technology assessment. Technologies underlying weapons of mass destruction.

[B49] Cronin A. K. (2003). Terrorist motivations for chemical and biological weapons use: Placing the threat in context. Rep. Congr. 23.03. 10.1080/1475179042000305778

[B50] Danzig R. Sageman M. Leighton T. Hough L. Yuki H. Kotani R. (2012). Aum Shinrikyo: Insights into how terrorists develop biological and chemical weapons. Second Edition. Center for a New American Security. Available at: https://www.files.ethz.ch/isn/156879/CNAS_AumShinrikyo_SecondEdition_English.pdf.

[B51] Dassanayake M. K. Khoo T-J. An J. (2021). Antibiotic resistance modifying ability of phytoextracts in anthrax biological agent *Bacillus anthracis* and emerging superbugs: A review of synergistic mechanisms. Ann. Clin. Microbiol. Antimicrob. 20, 79. 10.1186/s12941-021-00485-0 34856999 PMC8641154

[B52] De B. K. Bragg S. L. Sanden G. N. Wilson K. E. Diem L. A. Marston C. K. (2002). A two-component direct fluorescent-antibody assay for rapid identification of *Bacillus anthracis* . Emerg. Infect. Dis. 8, 1060–1065. 10.3201/eid0810.020392 12396916 PMC2730293

[B53] Deutsche Forschungsgemeinschaft and Deutsche Akademie der Naturforscher Leopoldina (2014). Scientific freedom and scientific responsibility recommendations for handling security-relevant research.

[B54] DiEuliis D. (2022). Perspective: The rapidly expanding need for biosecurity by design. BioDesign Res. 2022, 1–3. 10.34133/2022/9809058 PMC10521668 37850149

[B55] Dong X. Katzbaer R. R. Chitara B. Han L. Yang L. Schaak R. E. (2022). Optimizing the synergistic effect of CuWO 4/CuS hybrid composites for photocatalytic inactivation of pathogenic bacteria. Environ. Sci. Nano. 9, 4283–4294. 10.1039/D2EN00361A

[B56] Dunlap G. Pauwels E. (2017). The intelligent and connected bio-labs of the future: Promise and peril in the fourth industrial revolution. Available at: https://www.wilsoncenter.org/sites/default/files/media/documents/publication/dunlap_pauwels_intelligent_connected_biolabs_of_future.pdf (Accessed November 3, 2022).

[B57] Duriez E. Goossens P. L. Becher F. Ezan E. (2009). Femtomolar detection of the anthrax edema factor in human and animal plasma. Anal. Chem. 81, 5935–5941. 10.1021/ac900827s 19522516

[B58] Durrant G. R. (2002). Bioterrorism: The current threat. J. R. Soc. Med. 95, 609–611. 10.1177/014107680209501209 12461149 PMC1279289

[B59] EBRC Engineering Biology Research Consortium (2022). Public comment: Draft revised guidance points of consideration from an EBRC guidance workshop.

[B60] ECDC (2013). Scientific Opinion on the public health hazards to be covered by inspection of meat (bovine animals). EFSA J. 11, 3266. 10.2903/j.efsa.2013.3266 PMC7163758 32313569

[B61] ECDC. COMMUNICABLE (2022). Disease threats report.

[B62] Ehling-Schulz M. Lereclus D. Koehler T. M. (2019). The Bacillus cereus group: Bacillus species with pathogenic potential. Microbiol. Spectr. 7, 875–902. 10.1128/microbiolspec.GPP3-0032-2018 PMC6530592 31111815

[B63] Eisenstein M. (2020). How to build a genome. Nature 578, 633–635. 10.1038/d41586-020-00511-9 32094921

[B64] Engineering Biology Research Consortium Security Working Group (2022). Security screening in synthetic DNA synthesis recommendations for updated federal guidance. A policy paper by the engineering biology research Consortium security working group. Available at: https://ebrc.org/wp-content/uploads/2022/04/EBRC-2022-Security-Screening-in-Synthetic-DNA-Synthesis.pdf.

[B65] Epstein E. J. (2010). Edward jay Epstein: The anthrax attacks remain unsolved. New York, NY: The Wall Street Journal. Available at: https://www.wsj.com/articles/SB10001424052748704541004575011421223515284 (Accessed March 31, 2023).

[B66] Epstein G. L. (2023). Private-sector research could pose a pandemic risk. Here's what to do about it. Bull. Atomic Sci. Available at: https://thebulletin.org/2023/02/private-sector-research-could-pose-a-pandemic-risk-heres-what-to-do-about-it/#post-heading.

[B67] euroCRIS (2016). UNESCO/COAR joint statement on Open Access | euroCRIS. [updated 31 Mar 2023; cited 31 Mar 2023]. Available at: https://eurocris.org/news/unescocoar-joint-statement-open-access.

[B68] European Union (2021). Regulation (EU) 2021/821 of the European Parliament and of the Council of 20 May 2021 setting up a Union regime for the control of exports, brokering, technical assistance, transit and transfer of dual-use items (recast) PE/54/2020/REV/2. Available at: https://eur-lex.europa.eu/legal-content/EN/TXT/?uri=celex%3A32021R0821.

[B69] Favaro M. Renic N. Kühn U. (2022). Negative multiplicity: Forecasting the future impact of emerging technologies on international stability and human security. Research Report No. 10.

[B70] Fetter S. (1991). Ballistic missiles and weapons of mass destruction: What is the threat? What should be done. Int. Secur. 16, 5–42. 10.2307/2539050

[B71] Final B. W. C. (1991). Declaration of the third review conference. BWC/CONF. III/23.

[B72] Foresight U. N. D. P. (2018). Manual. Empowered futures for the 2030 agenda. For the 2030 agenda. Singapore.

[B73] Frischknecht F. (2003). The history of biological warfare. EMBO Rep. 4, 1–10. 10.1002/9783527621620.ch1 PMC1326439 12789407

[B74] Fujinami Y. Hirai Y. Sakai I. Yoshino M. Yasuda J. (2007). Sensitive detection of *Bacillus anthracis* using a binding protein originating from gamma-phage. Microbiol. Immunol. 51, 163–169. 10.1111/j.1348-0421.2007.tb03894.x 17310083

[B75] GenBank (1995). Bacillus anthracis edema factor gene (cya) gene, complete cds. Available at: https://www.ncbi.nlm.nih.gov/nuccore/M24074.1.

[B76] Gholami M. Moghbeli M. Kafilzadeh F. Kargar M. Torbati M. B. Tavizi A. (2021). Production of recombinant lethal factor of *Bacillus anthracis* in *Bacillus subtilis* . Prep. Biochem. Biotechnol. 51, 9–15. 10.1080/10826068.2020.1762215 32393098

[B77] Ginghina R-E. Toader G. Purica M. Bratu A-E. Lazaroaie C. Tiganescu T-V. (2022). Antimicrobial activity and degradation ability study on nanoparticle-enriched formulations specially designed for the neutralization of real and simulated biological and chemical warfare agents. Pharm. (Basel) 15, 97. 10.3390/ph15010097 PMC8778455 35056158

[B78] Granum P. E. (2017). Spotlight on *Bacillus cereus* and its food poisoning toxins. FEMS Microbiol. Lett. 364. 10.1093/femsle/fnx071 28521335

[B79] Green B. D. Battisti L. Koehler T. M. Thorne C. B. Ivins B. E. (1985). Demonstration of a capsule plasmid in *Bacillus anthracis* . Infect. Immun. 49, 291–297. 10.1128/iai.49.2.291-297.1985 3926644 PMC262013

[B81] Greenberg D. L. Busch J. D. Keim P. Wagner D. M. (2010). Identifying experimental surrogates for *Bacillus anthracis* spores: A review. Investig. Genet. 1, 4. 10.1186/2041-2223-1-4 PMC2988482 21092338

[B82] Grossman A. D. Losick R. (1988). Extracellular control of spore formation in *Bacillus subtilis* . Proc. Natl. Acad. Sci. 85, 4369–4373. 10.1073/pnas.85.12.4369 3132711 PMC280430

[B83] Gurkan C. Ellar D. J. (2005). Recombinant production of bacterial toxins and their derivatives in the methylotrophic yeast *Pichia pastoris* . Microb. Cell. Fact. 4, 33. 10.1186/1475-2859-4-33 16336647 PMC1325036

[B84] Haas C. N. (2002). The role of risk analysis in understanding bioterrorism. Risk Anal. 22, 671–677. 10.1111/0272-4332.00239 12224741

[B85] Hao R. Wang D. Zhang X. Zuo G. Wei H. Yang R. (2009). Rapid detection of *Bacillus anthracis* using monoclonal antibody functionalized QCM sensor. Biosens. Bioelectron. 24, 1330–1335. 10.1016/j.bios.2008.07.071 18804365

[B86] Hao R-Z. Song H-B. Zuo G-M. Yang R-F. Wei H-P. Wang D-B. (2011). DNA probe functionalized QCM biosensor based on gold nanoparticle amplification for *Bacillus anthracis* detection. Biosens. Bioelectron. 26, 3398–3404. 10.1016/j.bios.2011.01.010 21315574

[B87] Hemming D. Macneill K. (2020). Use of meteorological data in biosecurity. Emerg. Top. Life Sci. 4, 497–511. 10.1042/ETLS20200078 32935835 PMC7803344

[B88] Hesse E. M. Godfred-Cato S. Bower W. A. (2022). Antitoxin use in the prevention and treatment of anthrax disease: A systematic review. Clin. Infect. Dis. 75, 432–S440. 10.1093/cid/ciac532 PMC9649430 36251559

[B89] Hoffmann S. A. Diggans J. Densmore D. Dai J. Knight T. Leproust E. (2023). Safety by design: Biosafety and biosecurity in the age of synthetic genomics. iScience 26, 106165. 10.1016/j.isci.2023.106165 36895643 PMC9988571

[B90] Hoffmaster A. R. Hill K. K. Gee J. E. Marston C. K. De B. K. Popovic T. (2006). Characterization of *Bacillus cereus* isolates associated with fatal pneumonias: Strains are closely related to *Bacillus anthracis* and harbor B. Anthracis virulence genes. J. Clin. Microbiol. 44, 3352–3360. 10.1128/JCM.00561-06 16954272 PMC1594744

[B91] Honein M. A. Hoffmaster A. R. (2022). Responding to the threat posed by anthrax: Updated evidence to improve preparedness. Clin. Infect. Dis. 75, S339–S340. 10.1093/cid/ciac567 36251547 PMC9649413

[B92] Hoose A. Vellacott R. Storch M. Freemont P. S. Ryadnov M. G. (2023). DNA synthesis technologies to close the gene writing gap. Nat. Rev. Chem. 7, 144–161. 10.1038/s41570-022-00456-9 36714378 PMC9869848

[B93] Huan T. N. Ha V. T. T. Le Hung Q. Yoon M-Y. Han S-H. Chung H. (2009). Square wave voltammetric detection of Anthrax utilizing a peptide for selective recognition of a protein biomarker. Biosens. Bioelectron. 25, 469–474. 10.1016/j.bios.2009.08.002 19729294

[B94] Huang E. Pillai S. K. Bower W. A. Hendricks K. A. Guarnizo J. T. Hoyle J. D. (2015). Antitoxin treatment of inhalation anthrax: A systematic review. Health secur. 13, 365–377. 10.1089/hs.2015.0032 26690378 PMC4710135

[B95] Hughes R. A. Ellington A. D. (2017). Synthetic DNA synthesis and assembly: Putting the synthetic in synthetic biology. Cold Spring Harb. Perspect. Biol. 9, a023812. 10.1101/cshperspect.a023812 28049645 PMC5204324

[B96] iGem Team (2017). Lethbridge. Biosecurity software. [updated 3 May 2022; cited 31 Mar 2023]. Available at: https://2017.igem.org/Team:Lethbridge/Software.

[B137] Miles I. Saritas O. Sokolov A. (Editors) (2016). Foresight for science, technology and innovation (Cham: Springer International Publishing).

[B97] Inglesby T. V. O'Toole T. Henderson D. A. Bartlett J. G. Ascher M. S. Eitzen E. (2002). Anthrax as a biological weapon, 2002: Updated recommendations for management. JAMA 287, 2236–2252. Available at:. 10.1001/jama.287.17.2236 https://jamanetwork.com/journals/jama/article-abstract/194886?casa_token=k4nZmKlEUccAAAAA:YYTX_xAapbWdfegBWn4yhkhT1nTHfcROMdHFlH52OSzdq06ra3CbgtWff-BfUeETPH6EX21B-A 11980524

[B98] Jackson B. A. (2001). Technology acquisition by terrorist groups: Threat assessment informed by lessons from private sector technology adoption. Stud. Confl. Terror. 24, 183–213. 10.1080/10576100151130270

[B99] Jansen H. J. Breeveld F. J. Stijnis C. Grobusch M. P. (2014). Biological warfare, bioterrorism, and biocrime. Clin. Microbiol. Infect. 20, 488–496. 10.1111/1469-0691.12699 24890710 PMC7129974

[B100] Jinek M. Chylinski K. Fonfara I. Hauer M. Doudna J. A. Charpentier E. (2012). A programmable dual-RNA-guided DNA endonuclease in adaptive bacterial immunity. Science 337, 816–821. 10.1126/science.1225829 22745249 PMC6286148

[B101] Johns Hopkins Center for Health Security (2023). Bacillus anthracis (Anthrax).

[B102] Jumper J. Evans R. Pritzel A. Green T. Figurnov M. Ronneberger O. (2021). Highly accurate protein structure prediction with AlphaFold. Nature 596, 583–589. 10.1038/s41586-021-03819-2 34265844 PMC8371605

[B103] Kaiser J. (2022). Making trouble. Science 378, 242–245. 10.1126/science.adf3764 36264794

[B104] Kaittanis C. Santra S. Santiesteban O. J. Henderson T. J. Perez J. M. (2011). The assembly state between magnetic nanosensors and their targets orchestrates their magnetic relaxation response. J. Am. Chem. Soc. 133, 3668–3676. 10.1021/ja1109584 21341659 PMC3053437

[B105] Kaufer A. M. Theis T. Lau K. A. Gray J. L. Rawlinson W. D. (2020). Biological warfare: The history of microbial pathogens, biotoxins and emerging threats. Microbiol. Aust. 41, 116. 10.1071/MA20031

[B106] Kaufmann A. F. Meltzer M. I. Schmid G. P. (1997). The economic impact of a bioterrorist attack: Are prevention and postattack intervention programs justifiable. Emerg. Infect. Dis. 3, 83–94. 10.3201/eid0302.970201 9204289 PMC2627615

[B107] Keim P. Smith K. L. Keys C. Takahashi H. Kurata T. Kaufmann A. (2001). Molecular investigation of the Aum Shinrikyo anthrax release in Kameido, Japan. J. Clin. Microbiol. 39, 4566–4567. 10.1128/JCM.39.12.4566-4567.2001 11724885 PMC88589

[B108] Kim D-J. Park H-C. Sohn I. Y. Jung J-H. Yoon O. J. Park J-S. (2013). Electrical graphene aptasensor for ultra-sensitive detection of anthrax toxin with amplified signal transduction. Small 9, 3352–3360. 10.1002/smll.201203245 23589198

[B109] Kim J. Gedi V. Lee S-C. Cho J-H. Moon J-Y. Yoon M-Y. (2015). Advances in anthrax detection: Overview of bioprobes and biosensors. Appl. Biochem. Biotechnol. 176, 957–977. 10.1007/s12010-015-1625-z 25987133

[B110] Koblentz G. D. (2020). Emerging technologies and the future of CBRN terrorism. Wash. Q. 43, 177–196. 10.1080/0163660X.2020.1770969

[B111] Kohler K. (2021). Strategic foresight: Knowledge, tools, and methods for the future. Zurich: ETH Zurich.

[B112] Kosal M. E. (2020). “Emerging life sciences: New challenges to strategic stability,” in Disruptive and game changing technologies in modern warfare. Development, use, and proliferation. Editor Kosal M. E. (Springer International Publishing), 31–48.

[B114] Kotiranta A. Lounatmaa K. Haapasalo M. (2000). Epidemiology and pathogenesis of *Bacillus cereus* infections. Microbes Infect. 2, 189–198. 10.1016/S1286-4579(00)00269-0 10742691

[B115] Kuklenyik Z. Boyer A. E. Lins R. Quinn C. P. Gallegos-Candela M. Woolfitt A. (2011). Comparison of MALDI-TOF-MS and HPLC-ESI-MS/MS for endopeptidase activity-based quantification of Anthrax lethal factor in serum. Anal. Chem. 83, 1760–1765. 10.1021/ac1030144 21302970

[B116] Kumar P. Ahuja N. Bhatnagar R. (2001). Purification of anthrax edema factor from *Escherichia coli* and identification of residues required for binding to anthrax protective antigen. Infect. Immun. 69, 6532–6536. 10.1128/IAI.69.10.6532-6536.2001 11553601 PMC98792

[B117] LegiScan. California Assembly Bill 1963 (2022). CA state legislature page for AB1963. [updated 28 Nov 2022; cited 31 Mar 2023]. Available at: https://legiscan.com/CA/text/AB1963/2021.

[B118] Leitenberg M. (2005). Assessing the biological weapons and bioterrorism threat. Carlisle Barracks PA. Carlisle, PA: Strategic Studies Institute U.S. Army War College.

[B119] Leitenberg M. (2001). Biological weapons in the twentieth century: A review and analysis. Crit. Rev. Microbiol. 27, 267–320. 10.1080/20014091096774 11791799

[B120] Leitenberg M. Zilinskas R. A. (2012). “The Soviet biological weapons program: A history,” in The Soviet biological weapons program: A history. Editors Leitenberg M. Zilinskas R. A. Kuhn J. H.

[B121] Lentzos F. (2016). Biology’s misuse potential. Connect. QJ 15, 48–64. 10.11610/Connections.15.2.04

[B122] Lentzos F. (2020). How to protect the world from ultra-targeted biological weapons. Bull. Atomic Sci. 76, 302–308. 10.1080/00963402.2020.1846412

[B123] Levinson R. (2022). West curbs Russia’s access to weather data that some fear could aid attack planning in Ukraine. Reuters.

[B124] Lima-Pérez J. López-Pérez M. Viniegra-González G. Loera O. (2019). Solid-state fermentation of *Bacillus thuringiensis* var kurstaki HD-73 maintains higher biomass and spore yields as compared to submerged fermentation using the same media. Bioprocess Biosyst. Eng. 42, 1527–1535. 10.1007/s00449-019-02150-5 31115662

[B125] MacIntyre C. R. (2015). Biopreparedness in the age of genetically engineered pathogens and open access science: An urgent need for a paradigm shift. Mil. Med. 180, 943–949. 10.7205/MILMED-D-14-00482 26327545 PMC7107569

[B126] Makino S. Sasakawa C. Uchida I. Terakado N. Yoshikawa M. (1988). Cloning and CO2-dependent expression of the genetic region for encapsulation from *Bacillus anthracis* . Mol. Microbiol. 2, 371–376. 10.1111/j.1365-2958.1988.tb00041.x 2456447

[B127] Makino S. Uchida I. Terakado N. Sasakawa C. Yoshikawa M. (1989). Molecular characterization and protein analysis of the cap region, which is essential for encapsulation in *Bacillus anthracis* . J. Bacteriol. 171, 722–730. 10.1128/jb.171.2.722-730.1989 2536679 PMC209657

[B128] Malik S. Muhammad K. Waheed Y. (2023). Nanotechnology: A revolution in modern industry. Molecules 28, 661. 10.3390/molecules28020661 36677717 PMC9865684

[B129] Manoharan S. Taylor-Joyce G. Brooker T. A. Hernández Rodríguez C. S. Hapeshi A. Baldwin V. (2023). From cereus to anthrax and back again: Assessment of the temperature-dependent phenotypic switching in the “cross-over” strain *Bacillus cereus* G9241. Front. Microbiol. 14, 1113562. 10.3389/fmicb.2023.1113562 36937299 PMC10017872

[B130] Marston C. K. Ibrahim H. Lee P. Churchwell G. Gumke M. Stanek D. (2016). Anthrax toxin-expressing *Bacillus cereus* isolated from an anthrax-like eschar. PLoS One 11, e0156987. 10.1371/journal.pone.0156987 27257909 PMC4892579

[B131] Matsumoto G. (2003). Bioterrorism. Anthrax powder: State of the art. Science 302, 1492–1497. 10.1126/science.302.5650.1492 14645823

[B132] Mauroni A. (2022). On biological war. Fort Leavenworth, KS: MILITARY REVIEW. Available at: https://www.armyupress.army.mil/Portals/7/military-review/Archives/English/MJ-22/Mauroni/Mauroni.pdf.

[B133] May M. (2021). After COVID-19 successes, researchers push to develop mRNA vaccines for other diseases. Nat. Med. 27, 930–932. 10.1038/s41591-021-01393-8 34059823

[B134] McGovern J-P. Shih W. Y. Shih W-H. (2007). *In situ* detection of *Bacillus anthracis* spores using fully submersible, self-exciting, self-sensing PMN-PT/Sn piezoelectric microcantilevers. Analyst 132, 777–783. 10.1039/B704579D 17646877

[B113] Kosal M. E. (Editor) (2021). “Proliferation of weapons-and dual-use technologies,” Diplomatic, information, military, and economic approaches (Springer International Publishing.).

[B135] Meselson M. Guillemin J. Hugh-Jones M. Langmuir A. Popova l. Shelokov A. (1994). The Sverdlovsk anthrax outbreak of 1979. Science 266, 1202–1208. 10.1126/science.7973702 7973702

[B136] Migone T-S. Bolmer S. Zhong J. Corey A. Vasconcelos D. Buccellato M. (2015). Added benefit of raxibacumab to antibiotic treatment of inhalational anthrax. Antimicrob. Agents Chemother. 59, 1145–1151. 10.1128/AAC.04606-14 25487792 PMC4335881

[B138] Miller T. E. Beneyton T. Schwander T. Diehl C. Girault M. McLean R. (2020). Light-powered CO2 fixation in a chloroplast mimic with natural and synthetic parts. Science 368, 649–654. 10.1126/science.aaz6802 32381722 PMC7610767

[B139] Min S. Lee B. Yoon S. (2016). Deep learning in bioinformatics. Brief. Bioinform bbw068 18, 851–869. 10.1093/bib/bbw068 27473064

[B140] Moayeri M. Leppla S. H. Vrentas C. Pomerantsev A. P. Liu S. (2015). Anthrax pathogenesis. Annu. Rev. Microbiol. 69, 185–208. 10.1146/annurev-micro-091014-104523 26195305

[B141] Mondange L. Tessier É. Tournier J-N. (2022). Pathogenic bacilli as an emerging biothreat. Pathogens 11, 1186. 10.3390/pathogens11101186 36297243 PMC9609551

[B142] Moran J. P. (2021). Red team or red herring? Lessons learned from the policy counter terrorism evaluation group. Int. J. Intell. Secur. Public Aff. 23, 400–424. 10.1080/23800992.2021.2014504

[B143] Morris S. A. Boyack K. W. (2005). Visualizing 60 Years of anthrax research. Proc. ISSI, 45–55.

[B144] Morse S. A. (2014). Pathogen security-help or hindrance. Front. Bioeng. Biotechnol. 2, 83. 10.3389/fbioe.2014.00083 25610829 PMC4285169

[B80] Green M. S. Zenilman J. Dani C. Wiser I. Balicer R. D. (Editors) (2007). Risk assessment and risk communication strategies in bioterrorism preparedness (Dordrecht: Springer).

[B145] Mwilu S. K. Aluoch A. O. Miller S. Wong P. Sadik O. A. Fatah A. A. (2009). Identification and quantitation of *Bacillus globigii* using metal enhanced electrochemical detection and capillary biosensor. Anal. Chem. 81, 7561–7570. 10.1021/ac900834e 19689112

[B146] Nakonieczna A. Rutyna P. Fedorowicz M. Kwiatek M. Mizak L. Łobocka M. (2022). Three novel bacteriophages, J5a, F16Ba, and z1a, specific for *Bacillus anthracis*, define a new clade of historical wbeta phage relatives. Viruses 14, 213. 10.3390/v14020213 35215807 PMC8878798

[B147] National Academies of Sciences (2018). “Engineering, and medicine,” in Biodefense in the age of synthetic biology (Washington (DC). 30629396

[B148] National Research Council (2004). Biotechnology research in an age of terrorism. Washington, DC: The National Academies Press. 25057686

[B149] National Research Council (2011b). BioWatch and public health surveillance. Evaluating systems for the early detection of biological threats. Washington D.C.: National Academies Press. 25032347

[B150] National Research Council (2011a). Review of the scientific approaches used during the FBI’s investigation of the 2001 anthrax letters. Washington (DC). 24983068

[B151] Nixdorff K. (2020). Developments in systems biology: Implications for health and biochemical security. Nonproliferation Rev. 27, 459–473. 10.1080/10736700.2020.1865632

[B152] OECD (2019). Strategic foresight for better policies.

[B153] Oh B. N. Lee S. Park H-Y. Baeg J-O. Yoon M-Y. Kim J. (2011). Sensitive fluorescence assay of anthrax protective antigen with two new DNA aptamers and their binding properties. Analyst 136, 3384–3388. 10.1039/c0an00978d 21743920

[B154] Okinaka R. T. Cloud K. Hampton O. Hoffmaster A. R. Hill K. K. Keim P. (1999). Sequence and organization of pXO1, the large *Bacillus anthracis* plasmid harboring the anthrax toxin genes. J. Bacteriol. 181, 6509–6515. 10.1128/JB.181.20.6509-6515.1999 10515943 PMC103788

[B155] Ozbolat I. T. Peng W. Ozbolat V. (2016). Application areas of 3D bioprinting. Drug Discov. Today 21, 1257–1271. 10.1016/j.drudis.2016.04.006 27086009

[B156] Pal S. Alocilja E. C. (2010). Electrically active magnetic nanoparticles as novel concentrator and electrochemical redox transducer in *Bacillus anthracis* DNA detection. Biosens. Bioelectron. 26, 1624–1630. 10.1016/j.bios.2010.08.035 20864333

[B157] Pannifer A. D. Wong T. Y. Schwarzenbacher R. Renatus M. Petosa C. Bienkowska J. (2001). Crystal structure of the anthrax lethal factor. Nature 414, 229–233. 10.1038/n35101998 11700563

[B158] Paris K. (2023). Genome editing and biological weapons. Assessing the risk of misuse. 1st ed. Cham: Springer International Publishing; Imprint Springer.

[B159] Park H-Y. Go H-Y. Kalme S. Mane R. S. Han S-H. Yoon M-Y. (2009). Protective antigen detection using horizontally stacked hexagonal ZnO platelets. Anal. Chem. 81, 4280–4284. 10.1021/ac900632n 19400578

[B160] Pethő-Kiss K. (2022). Addressing the threat of a bioterrorist attack by means of an unmanned drone. J. Appl. Secur. Res. 18, 495–518. 10.1080/19361610.2021.2018923

[B161] Pilcher H. R. (2003). Drug research: The ups and downs of lithium. Nature 425, 118–120. 10.1038/425118a 12968146

[B162] Pilo P. Frey J. (2018). Pathogenicity, population genetics and dissemination of *Bacillus anthracis* . Infect. Genet. Evol. J. Mol. Epidemiol. Evol. Genet. Infect. Dis. 64, 115–125. 10.1016/j.meegid.2018.06.024 29935338

[B163] Puzis R. Farbiash D. Brodt O. Elovici Y. Greenbaum D. (2020). Increased cyber-biosecurity for DNA synthesis. Nat. Biotechnol. 38, 1379–1381. 10.1038/s41587-020-00761-y 33247280

[B164] Quintiliani R. Jr Quintiliani R. (2003). Inhalational anthrax and bioterrorism. Curr. Opin. Pulm. Med. 9, 221–226. 10.1097/00063198-200305000-00011 12682568

[B165] Rasko D. A. Worsham P. L. Abshire T. G. Stanley S. T. Bannan J. D. Wilson M. R. (2011). *Bacillus anthracis* comparative genome analysis in support of the Amerithrax investigation. Proc. Natl. Acad. Sci. U. S. A. 108, 5027–5032. 10.1073/pnas.1016657108 21383169 PMC3064363

[B166] Rathish B. Pillay R. Wilson A. Pillay V. V. (2022). Comprehensive review of bioterrorism. Available at: https://www.ncbi.nlm.nih.gov/books/NBK570614/. 34033376

[B167] Read T. D. Peterson S. N. Tourasse N. Baillie L. W. Paulsen I. T. Nelson K. E. (2003). The genome sequence of *Bacillus anthracis* Ames and comparison to closely related bacteria. Nature 423, 81–86. 10.1038/nature01586 12721629

[B168] Read T. D. Salzberg S. L. Pop M. Shumway M. Umayam L. Jiang L. (2002). Comparative genome sequencing for discovery of novel polymorphisms in *Bacillus anthracis* . Science 296, 2028–2033. 10.1126/science.1071837 12004073

[B169] Reardon S. (2023). Stricter US guidelines for 'gain-of-function' research are on the way - maybe. Nature 614, 206–207. 10.1038/d41586-023-00257-0 36707714

[B170] Revill J. Borrie J. Lennane R. (2022). Back to the future for verification in the biological disarmament regime. Geneva: UNIDIR.

[B171] Revill J. Jefferson C. (2014). Tacit knowledge and the biological weapons regime. Sci. Public Policy 41, 597–610. 10.1093/scipol/sct090

[B172] Riedel S. (2005). Anthrax: A continuing concern in the era of bioterrorism. Proc. (Bayl Univ. Med. Cent. 18, 234–243. 10.1080/08998280.2005.11928074 16200179 PMC1200731

[B173] Riedel S. (2004). Biological warfare and bioterrorism: A historical review. Proc. (Bayl Univ. Med. Cent. 17, 400–406. 10.1080/08998280.2004.11928002 16200127 PMC1200679

[B174] Riesenberg D. Guthke R. (1999). High-cell-density cultivation of microorganisms. Appl. Microbiol. Biotechnol. 51, 422–430. 10.1007/s002530051412 10341426

[B175] Robertson D. L. Leppla S. H. (1986). Molecular cloning and expression in *Escherichia coli* of the lethal factor gene of *Bacillus anthracis* . Gene 44, 71–78. 10.1016/0378-1119(86)90044-2 3021591

[B176] Roffey R. Tegnell A. Elgh F. (2002). Biological warfare in a historical perspective: The official publication of the European Society of Clinical Microbiology and Infectious Diseases, Amsterdam. Clin. Microbiol. Infect. 8, 450–454. 10.1046/j.1469-0691.2002.00501.x 12197867

[B177] Rosenberg B. H. (2002). Analysis of the anthrax attacks: Federation of American Scientists, 9.

[B178] Rotz L. D. Khan A. S. Lillibridge S. R. Ostroff S. M. Hughes J. M. (2002). Public health assessment of potential biological terrorism agents. Emerg. Infect. Dis. 8, 225–230. 10.3201/eid0802.010164 11897082 PMC2732458

[B179] Salama S. Hansell L. (2005). Does intent equal capability? Al-qaeda and weapons of mass destruction. Nonproliferation Rev. 12, 615–653. 10.1080/10736700600601236

[B180] Sanz J. A. Dunlap G. Nolan N. O’Leary C. (2022). Biosecurity risks and governance in the age of synthetic biology. MIT Sci. Policy Rev. 3, 136–142. 10.38105/spr.x3nlyr0fkc

[B181] Savcı Ü. (2019). A bibliometric analysis of *Bacillus anthracis* research published between 1975 and 2018. J. Surg. Med. 10.28982/josam.618738

[B182] Scales S. E. Horney J. A. Federal, state, local, and other public health agency roles in preparedness. 2023:11–34. 10.1016/B978-0-323-98810-0.00002-8

[B183] Schmedes S. Budowle B. Microbial F. Schmedes S. Budowle B. (2019). Microbial forensics. Encyclopedia of microbiology. Amsterdam: Encyclopedia of Microbiology, 134–145. 10.1016/B978-0-12-801238-3.02483-1

[B184] Schuch R. Nelson D. Fischetti V. A. (2002). A bacteriolytic agent that detects and kills *Bacillus anthracis* . Nature 418, 884–889. 10.1038/nature01026 12192412

[B185] Seyfried G. Pei L. Schmidt M. (2014). European do-it-yourself (DIY) biology: Beyond the hope, hype and horror. Bioessays 36, 548–551. 10.1002/bies.201300149 24782329 PMC4158858

[B186] Sharan Y. (2007). “The bioterrorism threat,” in Risk assessment and risk communication strategies in bioterrorism preparedness. Editors Green M. S. Zenilman J. Dani Cohenn Wiser I. Balicer R. D. (Dordrecht: Springer), 45–54.

[B187] Sharma M. Swain P. K. Chopra A. P. Chaudhary V. K. Singh Y. (1996). Expression and purification of anthrax toxin protective antigen from *Escherichia coli* . Protein Expr. Purif. 7, 33–38. 10.1006/prep.1996.0005 9172780

[B188] Singh H. Kaur K. (2023). Role of nanotechnology in research fields: Medical sciences, military and tribology- A review on recent advancements, grand challenges and perspectives. Mater. Today Proc. 10.1016/j.matpr.2023.02.061

[B189] Singh S. K. Kuhn J. H. (Editors) (2019). Defense against biological attacks (Cham: Springer International Publishing).

[B190] Smith H. Keppie J. Stanley J. (1953). The chemical basis of the virulence of *Bacillus anthracis*. I. Properties of bacteria grown *in vivo* and preparation of extracts. Br. J. Exp. Pathol. 34, 477–485. 13106214 PMC2073529

[B191] Smith J. A. Sandbrink J. B. (2022). Biosecurity in an age of open science. PLoS Biol. 20, e3001600. 10.1371/journal.pbio.3001600 35421093 PMC9009689

[B192] South P. F. Cavanagh A. P. Liu H. W. Ort D. R. (2019). Synthetic glycolate metabolism pathways stimulate crop growth and productivity in the field. Science 363, eaat9077. 10.1126/science.aat9077 30606819 PMC7745124

[B193] Splino M. Patocka J. Prymula R. Chlibek R. (2005). Anthrax vaccines. Ann. Saudi Med. 25, 143–149. 10.5144/0256-4947.2005.143 15977694 PMC6147967

[B194] Spyer J. (2004). The Al-qa'ida network and weapons of mass destruction. Middle East Rev. Int. Aff. 8, 29–45.

[B195] Stanley J. L. Smith H. Sargeant K. (1961). Purification of factor I and recognition of a third factor of the anthrax toxin. J. Gen. Microbiol. 26, 49–66. 10.1099/00221287-26-1-49 13916257

[B196] Stemmer W. P. Crameri A. Ha K. D. Brennan T. M. Heyneker H. L. (1995). Single-step assembly of a gene and entire plasmid from large numbers of oligodeoxyribonucleotides. Gene 164, 49–53. 10.1016/0378-1119(95)00511-4 7590320

[B197] Stepanov A. V. Marinin L. I. Pomerantsev A. P. Staritsin N. A. (1996). Development of novel vaccines against anthrax in man. J. Biotechnol. 44, 155–160. 10.1016/0168-1656(95)00092-5 8717399

[B198] Sun T. Song J. Wang M. Zhao C. Zhang W. (2022). Challenges and recent progress in the governance of biosecurity risks in the era of synthetic biology. J. Biosaf. Biosecurity 4, 59–67. 10.1016/j.jobb.2022.02.002

[B199] Tang S. Moayeri M. Chen Z. Harma H. Zhao J. Hu H. (2009). Detection of anthrax toxin by an ultrasensitive immunoassay using europium nanoparticles. Clin. Vaccine Immunol. 16, 408–413. 10.1128/CVI.00412-08 19129473 PMC2650879

[B200] Tennenbaum M. Kosal M. E. (2021). “The interplay between frugal science and chemical and biological weapons: Investigating the proliferation risks of technology intended for humanitarian, disaster response, and international development efforts,” in Proliferation of weapons-and dual-use technologies. Diplomatic, information, military, and economic approaches. Editor Kosal M. E. (Springer International Publishing.), 134–152.

[B201] The Australia Group (2021). Control list of dual-use biological equipment and related technology and software. Available at: https://www.dfat.gov.au/publications/minisite/theaustraliagroupnet/site/en/dual_biological.html (Accessed November 3, 2022).

[B202] The Australian Department of Foreign Affairs and Trade (1958). Australia group. Available at: https://www.dfat.gov.au/publications/minisite/theaustraliagroupnet/site/en/origins.html.

[B203] The Nuclear Threat Initiative (2023). Preventing the misuse of DNA synthesis technology. [updated 17 Feb 2023; cited 31 Mar 2023]. Available at: https://www.nti.org/about/programs-projects/project/preventing-the-misuse-of-dna-synthesis-technology/.

[B204] Thorne C. B. (1960). Biochemical properties of virulent and avirulent strains of *Bacillus anthracis* . Ann. N. Y. Acad. Sci. 88, 1024–1033. 10.1111/j.1749-6632.1960.tb20094.x 13776703

[B205] Tian P. Ye W. Zhang X. Tong Y. Qian P-Y. Tong R. (2022). Ten-step asymmetric total syntheses of potent antibiotics anthracimycin and anthracimycin B. Chem. Sci. 13, 12776–12781. 10.1039/d2sc05049h 36519065 PMC9645392

[B206] Tocchetti S. Aguiton S. A. (2015). Is an FBI agent a DIY biologist like any other? A cultural analysis of a biosecurity risk. Sci. Technol. Hum. Values 40, 825–853. 10.1177/0162243915589634

[B207] Tournier J-N. Mohamadzadeh M. (2010). Key roles of dendritic cells in lung infection and improving anthrax vaccines. Trends Mol. Med. 16, 303–312. 10.1016/j.molmed.2010.04.006 20554248

[B208] Tournier J-N. Rougeaux C. Biot F. V. Goossens P. L. (2019). Questionable efficacy of therapeutic antibodies in the treatment of anthrax. mSphere 4, e00282-19. 10.1128/mSphere.00282-19 31217301 PMC6584371

[B209] Tournier J-N. Ulrich R. G. Quesnel-Hellmann A. Mohamadzadeh M. Stiles B. G. (2009). Anthrax, toxins and vaccines: A 125-year journey targeting *Bacillus anthracis* . Expert Rev. Anti Infect. Ther. 7, 219–236. 10.1586/14787210.7.2.219 19254170

[B211] Trump B. D. Florin M-V. Perkins E. Linkov I. (2021b). “Biosecurity for synthetic biology and emerging biotechnologies: Critical challenges for governance,” in Emerging threats of synthetic biology and biotechnology. Addressing security and resilience issues. Editors Trump B. D. Florin M-V. Perkins E. Linkov I. 1st ed. (Dordrecht: Springer Netherlands; Imprint Springer), 1–12.

[B212] Tufts J. A. M. Calfee M. W. Lee S. D. Ryan S. P. (2014). *Bacillus thuringiensis* as a surrogate for *Bacillus anthracis* in aerosol research. World J. Microbiol. Biotechnol. 30, 1453–1461. 10.1007/s11274-013-1576-x 24338558

[B213] Turnbull P. C. (2014). World Health Organization Guidelines for the surveillance and control of anthrax in humans and animals. 3rd edition. Geneva: WHO.

[B214] United Nations (1972). Convention on the prohibition of the development, production and stockpiling of bacteriological (biological) and toxin weapons and on their destruction. Available at: https://ihl-databases.icrc.org/assets/treaties/450-IHL-68-EN.pdf .

[B215] United Nations (2007). Monitoring, verification and inspection commission, compendium of Iraq’s proscribed weapons programmes in the chemical, biological and missile areas. New York, NY.

[B216] United Nations Security Council (2004). Res 1540 UN doc S/RES/1540. Available at: https://documents-dds-ny.un.org/doc/UNDOC/GEN/N04/328/43/PDF/N0432843.pdf?OpenElement.

[B217] United States Arms Control and DIsarmament Agency (1971). “Remarks on biological warfare by dr. Joshua lederberg to the conference of the committee on disarmament,” in Documents on disarmament 1970 (Washington, D. C: U.S. Government Printing Office), 355–362.

[B218] Urban-Sorensen K. (2018). Biological warfare and environmental decontamination post-exposure: Who is responsible. J. Biosecurity, Biosaf. Biodefense Law 9. 10.1515/jbbbl-2018-0007

[B219] US Congress (2004). Public law 108–276 118 stat 835. Available at: https://www.govinfo.gov/content/pkg/PLAW-108publ276/pdf/PLAW-108publ276.pdf .

[B220] U.S. Department of Health and Human Services (2010). Screening framework guidance for providers of synthetic double-stranded DNA. Fed. Regist. 75 (197), 62820–62832.

[B221] USDOJ (2010). Amerithrax investigative summary. Available at: https://www.justice.gov/archive/amerithrax/docs/amx-investigative-summary.pdf.

[B222] Venter J. C. Glass J. I. Hutchison C. A. Vashee S. (2022). Synthetic chromosomes, genomes, viruses, and cells. Cell. 185, 2708–2724. 10.1016/j.cell.2022.06.046 35868275 PMC9347161

[B223] Vogel K. (2006). Bioweapons proliferation: Where science studies and public policy collide. Soc. Stud. Sci. 36, 659–690. 10.1177/0306312706059460

[B224] Wang D-B. Cui M-M. Li M. Zhang X-E. (2021a). Biosensors for the detection of *Bacillus anthracis* . Acc. Chem. Res. 54, 4451–4461. 10.1021/acs.accounts.1c00407 34846836

[B225] Wang L. Song J. Zhang W. (2021b). Tianjin Biosecurity Guidelines for codes of conduct for scientists: Promoting responsible sciences and strengthening biosecurity governance. J. Biosaf. Biosecurity 3, 82–83. 10.1016/j.jobb.2021.08.001

[B226] Wang M. (2013). From home range dynamics to population cycles: Validation and realism of a common vole population model for pesticide risk assessment. Integr. Environ. Assess. Manag. 9, 294–307. 10.1002/ieam.1377 23086922

[B227] Wang Y. Wang D. Wang X. Tao H. Feng E. Zhu L. (2019). Highly efficient genome engineering in *Bacillus anthracis* and *Bacillus cereus* using the CRISPR/Cas9 system. Front. Microbiol. 10, 1932. 10.3389/fmicb.2019.01932 31551942 PMC6736576

[B228] Wassenaar Arrangement Secretariat (1995). The wassenaar arrangement on export controls for conventional arms and dual-use goods and technologies. Available at: https://www.wassenaar.org/app/uploads/2021/12/Public-Docs-Vol-I-Founding-Documents.pdf.

[B229] WHO (2023). Anthrax. Available at: https://www.emro.who.int/health-topics/antrhax/index.html.

[B230] WHO (2022). Global guidance framework for the responsible use of the life sciences: Mitigating biorisks and governing dual-use research.

[B231] Wolfe D. N. Espeland E. M. Gao Y. Lu Di Blatner G. Amass K. (2020). Evaluation of BioThrax® and AV7909 anthrax vaccines in adults 66 years of age or older. Vaccine 38, 7970–7976. 10.1016/j.vaccine.2020.10.053 33129609

[B232] World Health Organization (1970). Health aspects of chemical and biological weapons: Report of a. Geneva: WHO group of consultants.

[B233] World Health Organization (2022). Towards a global guidance framework for the responsible use of life sciences: Summary report of consultations on the principles, gaps and challenges of biorisk management. Geneva: No. WHO/SCI/RFH/2022.01.

[B234] Xu J. Bai X. Zhang X. Yuan B. Lin L. Guo Y. (2023). Development and application of DETECTR-based rapid detection for pathogenic *Bacillus anthracis* . Anal. Chim. Acta 1247, 340891. 10.1016/j.aca.2023.340891 36781250

[B235] Yassif J. Jaime Yassif on need for better safeguarding of bioscience. An expert says bad-faith actors can too easily get hold of dangerous biotechnology. The Economist. 2022. Available at: https://www.nti.org/about/people/jaime-yassif-phd/(Accessed 2022 December 20).

[B236] Yeom J. Park J. S. Jung S-W. Lee S. Kwon H. Yoo S. M. (2023). High-throughput genetic engineering tools for regulating gene expression in a microbial cell factory. Crit. Rev. Biotechnol. 43, 82–99. 10.1080/07388551.2021.2007351 34957867

[B237] Zahavy E. Heleg-Shabtai V. Zafrani Y. Marciano D. Yitzhaki S. (2010). Application of fluorescent nanocrystals (q-dots) for the detection of pathogenic bacteria by flow-cytometry. J. Fluoresc. 20, 389–399. 10.1007/s10895-009-0546-z 19826932

[B238] Zasada A. A. (2020). Detection and identification of Bacillus anthracis: From conventional to molecular microbiology methods. Microorganisms 8, 125. 10.3390/microorganisms8010125 31963339 PMC7023132

[B239] Zhang B. Li Y. Zhang Y. Qiao H. He J. Yuan Q. (2019). High-cell-density culture enhances the antimicrobial and freshness effects of *Bacillus subtilis* S1702 on table grapes (Vitis vinifera cv. Kyoho). Food Chem. 286, 541–549. 10.1016/j.foodchem.2019.02.050 30827645

[B240] Zhang D. Hussain A. Manghwar H. Xie K. Xie S. Zhao S. (2020). Genome editing with the CRISPR-cas system: An art, ethics and global regulatory perspective. Plant Biotechnol. J. 18, 1651–1669. 10.1111/pbi.13383 32271968 PMC7336378

[B241] Zhang L. Gronvall G. K. (2020). Red teaming the biological sciences for deliberate threats. Terror. Political Violence 32, 1225–1244. 10.1080/09546553.2018.1457527

[B242] Zhang N. Appella D. H. (2007). Colorimetric detection of anthrax DNA with a Peptide nucleic acid sandwich-hybridization assay. J. Am. Chem. Soc. 129, 8424–8425. 10.1021/ja072744j 17569540

[B243] Zilinskas R. A. (1997). Iraq's biological weapons: The past as future. Jama 278, 418–424. 10.1001/jama.278.5.418 9244334

[B244] Zilinskas R. A. (2020). Possible terrorist use of modern biotechnology techniques. Rome, Italy: Conference on Biosecurity and Bioterrorism Istituto Diplomatico “Mario Toscano” Villa Madama.

[B245] Zilinskas R. A. (2014). The Soviet biological weapons program and its legacy in today’s Russia. Washington, DC: National Defense University Press.

