# Supplementary material for: Anthrax revisited: how assessing the unpredictable can improve biosecurity
Source: Front Bioeng Biotechnol. 2023 Sep 19;11:1215773. doi: 10.3389/fbioe.2023.1215773 (PMC10546327; doi:10.3389/fbioe.2023.1215773)
Supplement: Supplementary file 1 [file Table1.DOCX]

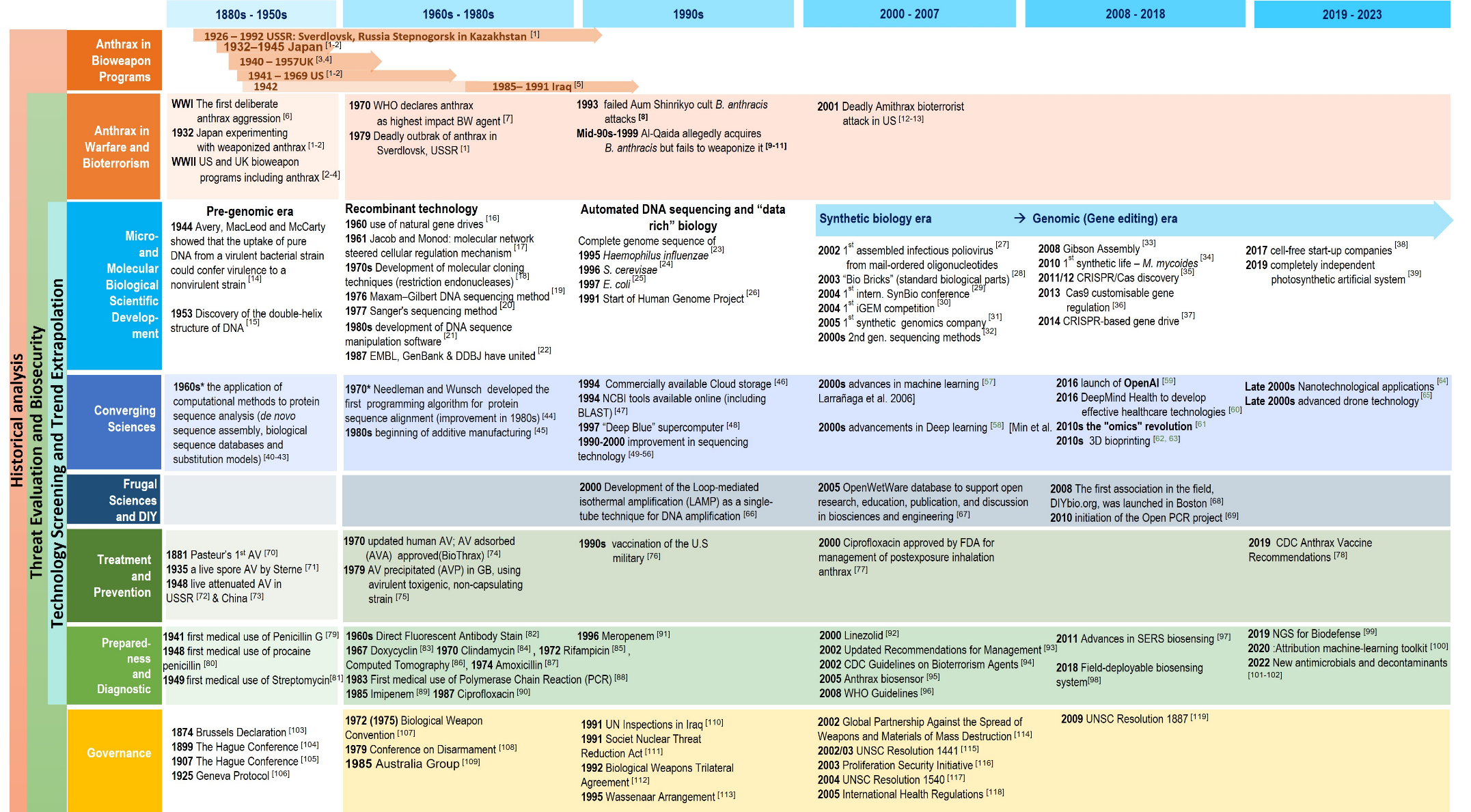


[1] [2] [3] [4] [5,6] [7] [8] [9] [10] [11,12] [13] [14] [15] [16] [17] [18] [19] [20] [21] [22] [23] [24] [25] [26] [27] [28] [29] [30] [31] [32] [33] [34] [35] [36] [37] [38] [39] [40] [41] [42] [43] [44] [45] [46] [47] [48] [49] [50] [51] [52] [53] [54] [55] [56] [57] [58] [59] [60] [61] [62] [63] [64] [65] [66] [67] [68] [69] [70] [71] [72] [73] [74] [75] [76] [77] [78] [79] [80] [81] [82] [83] [84] [85] [86] [87] [88] [89] [90] [91] [92] [93] [94] [95] [96] [97] [98] [99] [100] [101,102] [103] [104] [105] [106] [107] [108] [109] [110] [111] [112] [113] [114] [115] [116] [117] [118] [119]

**List of abbreviations**

3D Three dimensional

AV (46) Anthrax vaccine

AVA AV adsorbed

AVP AV precipitated

BLAST Basic Local Alignment Search Tool

CDC Centerd for Disease Control and Prevention

CRISPR Clustered regularly interspaced short palindromic repeats

CRISPR/Cas Clustered regularly interspaced short palindromic repeats/CRISPR-associated protein 9

dCas9 deactivated CRISPR-associated protein 9

DDBJ DNA Data Bank of Japan

DNA Deoxyribonucleic acid

EMBL European Molecular Biology Laboratory

iGEM International Genetically Engineered Machine

LAMP loop-mediated isothermal amplification

NCBI National Center for Biotechnology Information

NGS Next-Generation Sequencing

PCR Polymerase Chain Reaction

SERS Surface-enhanced Raman spectroscopy

SynBio Synthetic Biology

UK United Kingdom

UN United Nations

UNSC United Nations Security Council

US Unites States of America

USSR Union of Soviet Socialist Republics

WHO World Health Organization

**References**

References

**1**. Frischknecht F. The History of Biological Warfare. EMBO reports. 2003; 4:547–52. doi: 10.1002/9783527621620.ch1.

**2**. Kaufer AM, Theis T, Lau KA, Gray JL, Rawlinson WD. Biological warfare: the history of microbial pathogens, biotoxins and emerging threats. Microbiol Aust. 2020; 41:116. doi: 10.1071/MA20031.

**3**. Kosal ME, editor. Proliferation of Weapons-and Dual-Use Technologies. Diplomatic, Information, Military, and Economic Approaches. Springer International Publishing.; 2021.

**4**. Lentzos F. Biology’s Misuse Potential. Connections QJ. 2016; 15:48–64. doi: 10.11610/Connections.15.2.04.

**5**. World Health Organization. Towards a global guidance framework for the responsible use of life sciences: summary report of consultations on the principles, gaps and challenges of biorisk management. (No. WHO/SCI/RFH/2022.01). ; May 2022.

**6**. Ainscough MJ. Next Generation Bioweapons: The Technology of Genetic Engineering Applied to Biowarfare and Bioterrorism. ; April 2002.

**7**. Paris K. Genome Editing and Biological Weapons. Assessing the Risk of Misuse. 1st ed. Cham: Springer International Publishing; Imprint Springer; 2023.

**8**. United Nations. Convention on the Prohibition of the Development, Production and Stockpiling of Bacteriological (Biological) and Toxin Weapons and on their Destruction. April 10, 1972. 1972. Available from: https://ihl-databases.icrc.org/assets/treaties/450-IHL-68-EN.pdf.

**9**. The Australian Department of Foreign Affairs and Trade (DFAT). Australia Group. June, 1958. 1985. Available from: https://www.dfat.gov.au/publications/minisite/theaustraliagroupnet/site/en/origins.html.

**10**. Wassenaar Arrangement Secretariat. The Wassenaar Arrangement on Export Controls for Conventional Arms and Dual-Use Goods and Technologies. December 19, 1995. 1995. Available from: https://www.wassenaar.org/app/uploads/2021/12/Public-Docs-Vol-I-Founding-Documents.pdf.

**11**. United Nations Security Council (UNSC). Res 1540 UN Doc S/RES/1540. 2004. Available from: https://documents-dds-ny.un.org/doc/UNDOC/GEN/N04/328/43/PDF/N0432843.pdf?OpenElement.

**12**. DiEuliis D. Perspective: The Rapidly Expanding Need for Biosecurity by Design. BioDesign Research. 2022; 2022:1–3. doi: 10.34133/2022/9809058.

**13**. Trump BD, Florin M-V, Perkins E, Linkov I, editors. Emerging Threats of Synthetic Biology and Biotechnology. Addressing Security and Resilience Issues. 1st ed. Dordrecht: Springer Netherlands; Imprint Springer; 2021.

**14**. Venter JC, Glass JI, Hutchison CA, Vashee S. Synthetic chromosomes, genomes, viruses, and cells. Cell. 2022; 185:2708–24. doi: 10.1016/j.cell.2022.06.046 PMID: 35868275.

**15**. Miller TE, Beneyton T, Schwander T, Diehl C, Girault M, McLean R, et al. Light-powered CO2 fixation in a chloroplast mimic with natural and synthetic parts. Science. 2020; 368:649–54. doi: 10.1126/science.aaz6802 PMID: 32381722.

**16**. South PF, Cavanagh AP, Liu HW, Ort DR. Synthetic glycolate metabolism pathways stimulate crop growth and productivity in the field. Science. 2019; 363:eaat9077. Epub 2019/01/03. doi: 10.1126/science.aat9077 PMID: 30606819.

**17**. May M. After COVID-19 successes, researchers push to develop mRNA vaccines for other diseases. Nat Med. 2021; 27:930–2. doi: 10.1038/s41591-021-01393-8 PMID: 34059823.

**18**. MacIntyre CR. Biopreparedness in the Age of Genetically Engineered Pathogens and Open Access Science: An Urgent Need for a Paradigm Shift. Mil Med. 2015; 180:943–9. doi: 10.7205/MILMED-D-14-00482 PMID: 26327545.

**19**. Sun T, Song J, Wang M, Zhao C, Zhang W. Challenges and recent progress in the governance of biosecurity risks in the era of synthetic biology. Journal of Biosafety and Biosecurity. 2022; 4:59–67. doi: 10.1016/j.jobb.2022.02.002.

**20**. Sanz JA, Dunlap G, Nolan N, & O’Leary C. Biosecurity risks and governance in the age of synthetic biology. MIT Science Policy Review. 2022; 3:136–42. doi: 10.38105/spr.x3nlyr0fkc.

**21**. Singh SK, Kuhn JH, editors. Defense Against Biological Attacks. Volume I. Cham: Springer International Publishing; 2019.

**22**. Eisenstein M. How to build a genome. A powerful set of molecular tools helps synthetic biologists to assemble DNA of different sizes, from the gene to the chromosome scale. Nature. 2020; 578:633–5. doi: 10.1038/d41586-020-00511-9 PMID: 32094921.

**23**. Hoose A, Vellacott R, Storch M, Freemont PS, Ryadnov MG. DNA synthesis technologies to close the gene writing gap. Nat Rev Chem. 2023:1–18. Epub 2023/01/23. doi: 10.1038/s41570-022-00456-9 PMID: 36714378.

**24**. Yeom J, Park JS, Jung S-W, Lee S, Kwon H, Yoo SM. High-throughput genetic engineering tools for regulating gene expression in a microbial cell factory. Crit Rev Biotechnol. 2023; 43:82–99. Epub 2021/12/26. doi: 10.1080/07388551.2021.2007351 PMID: 34957867.

**25**. Jinek M, Chylinski K, Fonfara I, Hauer M, Doudna JA, Charpentier E. A programmable dual-RNA-guided DNA endonuclease in adaptive bacterial immunity. Science. 2012; 337:816–21. Epub 2012/06/28. doi: 10.1126/science.1225829 PMID: 22745249.

**26**. Zhang D, Hussain A, Manghwar H, Xie K, Xie S, Zhao S, et al. Genome editing with the CRISPR-Cas system: an art, ethics and global regulatory perspective. Plant Biotechnol J. 2020; 18:1651–69. Epub 2020/04/30. doi: 10.1111/pbi.13383 PMID: 32271968.

**27**. Min S, Lee B, Yoon S. Deep Learning in Bioinformatics. Brief Bioinform bbw068. 2016. doi: 10.48550/arXiv.1603.06430.

**28**. Jumper J, Evans R, Pritzel A, Green T, Figurnov M, Ronneberger O, et al. Highly accurate protein structure prediction with AlphaFold. Nature. 2021; 596:583–9. Epub 2021/07/15. doi: 10.1038/s41586-021-03819-2 PMID: 34265844.

**29**. Ozbolat IT, Peng W, Ozbolat V. Application areas of 3D bioprinting. Drug Discov Today. 2016; 21:1257–71. Epub 2016/04/13. doi: 10.1016/j.drudis.2016.04.006 PMID: 27086009.

**30**. Bracamonte AG. Current Advances in Nanotechnology for the Next Generation of Sequencing (NGS). Biosensors (Basel). 2023; 13. Epub 2023/02/12. doi: 10.3390/bios13020260 PMID: 36832027.

**31**. Malik S, Muhammad K, Waheed Y. Nanotechnology: A Revolution in Modern Industry. Molecules. 2023; 28. Epub 2023/01/09. doi: 10.3390/molecules28020661 PMID: 36677717.

**32**. Singh H, Kaur K. Role of nanotechnology in research fields: Medical sciences, military & tribology- A review on recent advancements, grand challenges and perspectives. Materials Today: Proceedings. 2023. doi: 10.1016/j.matpr.2023.02.061.

**33**. Brockmann K, Bauer S, Boulanin V. BIO PLUS X: Arms Control and the Convergence of Biology and Emerging Technologies. ; 2019.

**34**. Favaro M, Renic N, Kühn U. Negative Multiplicity: Forecasting the Future Impact of Emerging Technologies on International Stability and Human Security. Research Report No. 10. ; September 2022.

**35**. Kosal ME. Emerging Life Sciences: New Challenges to Strategic Stability. In: Kosal ME, editor. Disruptive and Game Changing Technologies in Modern Warfare. Development, Use, and Proliferation. Springer International Publishing.; 2020. pp. 31–48.

**36**. Cieslak TJ, Kortepeter MG, Wojtyk RJ, Jansen H-J, Reyes RA, Smith JO. Beyond the Dirty Dozen: A Proposed Methodology for Assessing Future Bioweapon Threats. Mil Med. 2018; 183:e59-e65. doi: 10.1093/milmed/usx004 PMID: 29401327.

**37**. National Academies of Sciences, Engineering, and Medicine. Biodefense in the Age of Synthetic Biology. Washington (DC); 2018.

**38**. Trump BD, Florin M-V, Perkins E, Linkov I. Biosecurity for Synthetic Biology and Emerging Biotechnologies: Critical Challenges for Governance. In: Trump BD, Florin M-V, Perkins E, Linkov I, editors. Emerging Threats of Synthetic Biology and Biotechnology. Addressing Security and Resilience Issues. 1st ed. Dordrecht: Springer Netherlands; Imprint Springer; 2021. pp. 1–12.

**39**. Caudle III LC. The biological warfare threat. Sidell, F. R., Takafuji, E. T., & Franz, D. R. (Hg.) 1997 Medical Aspects of Chemical. pp. 451–66.

**40**. Riedel S. Biological warfare and bioterrorism: a historical review. Proc (Bayl Univ Med Cent). 2004; 17:400–6. doi: 10.1080/08998280.2004.11928002 PMID: 16200127.

**41**. Hoffmann SA, Diggans J, Densmore D, Dai J, Knight T, Leproust E, et al. Safety by Design: Biosafety and Biosecurity in the Age of Synthetic Genomics. iScience. 2023:106165. doi: 10.1016/j.isci.2023.106165.

**42**. Yassif J. Jaime Yassif on need for better safeguarding of bioscience. An expert says bad-faith actors can too easily get hold of dangerous biotechnology. The Economist. 2022 Dec 20. Available from: https://www.nti.org/about/people/jaime-yassif-phd/ [updated 2022 Dec 20].

**43**. Tin D, Sabeti P, Ciottone GR. Bioterrorism: An analysis of biological agents used in terrorist events. Am J Emerg Med. 2022; 54:117–21. Epub 2022/02/06. doi: 10.1016/j.ajem.2022.01.056 PMID: 35152120.

**44**. Koblentz GD. Emerging Technologies and the Future of CBRN Terrorism. The Washington Quarterly. 2020; 43:177–96. doi: 10.1080/0163660X.2020.1770969.

**45**. Carlson CJ, Kracalik IT, Ross N, Alexander KA, Hugh-Jones ME, Fegan M, et al. The global distribution of *Bacillus anthracis* and associated anthrax risk to humans, livestock, and wildlife. Nat Microbiol. 2019; 4:1337–43. Epub 2019/05/13. doi: 10.1038/s41564-019-0435-4 PMID: 31086311.

**46**. Cole LA. Anthrax as a Weapon of War and Terrorism. In: Bergman NH, editor. Bacillus anthracis and Anthrax. ; 2011. pp. 295–308.

**47**. Johns Hopkins Center for Health Security. Bacillus anthracis (Anthrax). ; 11.01.2023.

**48**. Riedel S. Anthrax: a continuing concern in the era of bioterrorism. Proc (Bayl Univ Med Cent). 2005; 18:234–43. doi: 10.1080/08998280.2005.11928074 PMID: 16200179.

**49**. Acevedo MM, Carroll LM, Mukherjee M, Mills E, Xiaoli L, Dudley EG, et al. *Bacillus clarus* sp. nov. is a new *Bacillus cereus* group species isolated from soil. BioRxiv. 2019.

**50**. Bazinet AL. Pan-genome and phylogeny of *Bacillus cereus sensu lato*. BMC Evol Biol. 2017; 17:176. Epub 2017/08/02. doi: 10.1186/s12862-017-1020-1 PMID: 28768476.

**51**. Kotiranta A, Lounatmaa K, Haapasalo M. Epidemiology and pathogenesis of *Bacillus cereus* infections. Microbes Infect. 2000; 2:189–98. doi: 10.1016/S1286-4579(00)00269-0 PMID: 10742691.

**52**. Argôlo-Filho RC, Loguercio LL. *Bacillus thuringiensis* Is an Environmental Pathogen and Host-Specificity Has Developed as an Adaptation to Human-Generated Ecological Niches. Insects. 2013; 5:62–91. Epub 2013/12/24. doi: 10.3390/insects5010062 PMID: 26462580.

**53**. Granum PE. Spotlight on *Bacillus cereus* and its food poisoning toxins. FEMS Microbiol Lett. 2017; 364. doi: 10.1093/femsle/fnx071 PMID: 28521335.

**54**. Pilo P, Frey J. Pathogenicity, population genetics and dissemination of *Bacillus anthracis*. Infection, genetics and evolution: journal of molecular epidemiology and evolutionary genetics in infectious diseases. 2018; 64:115–25. Epub 2018/06/20. doi: 10.1016/j.meegid.2018.06.024 PMID: 29935338.

**55**. American Society for Microbiology. Identification of Bacillus cereus biovar anthracis. 2017. Available from: https://asm.org/Guideline/Identification-of-Bacillus-cereus-biovar-anthracis.

**56**. Savcı Ü. A bibliometric analysis of *Bacillus anthracis* research published between 1975 and 2018. Journal of Surgery and Medicine. 2019. doi: 10.28982/josam.618738.

**57**. Miles I, Saritas O, Sokolov A, editors. Foresight for Science, Technology and Innovation. Cham: Springer International Publishing; 2016.

**58**. UNDP. Foresight Manual. Empowered Futures for the 2030 Agenda. for the 2030 Agenda. Singapore; 2018.

**59**. OECD. Strategic Foresight for Better Policies. ; October 2019.

**60**. Moran JP. Red Team or Red Herring? Lessons Learned from the Policy Counter Terrorism Evaluation Group. The International Journal of Intelligence, Security, and Public Affairs. 2021; 23:400–24. doi: 10.1080/23800992.2021.2014504.

**61**. Zhang L, Gronvall GK. Red Teaming the Biological Sciences for Deliberate Threats. Terrorism and Political Violence. 2020; 32:1225–44. doi: 10.1080/09546553.2018.1457527.

**62**. Amanatidou E, Butter M, Carabias V, Konnola T, Leis M, Saritas O, et al. On concepts and methods in horizon scanning: Lessons from initiating policy dialogues on emerging issues. Science and Public Policy. 2012; 39:208–21. doi: 10.1093/scipol/scs017.

**63**. Bakhtin P, Saritas O, Chulok A, Kuzminov I, Timofeev A. Trend monitoring for linking science and strategy. Scientometrics. 2017; 111:2059–75. doi: 10.1007/s11192-017-2347-5.

**64**. Kohler K. Strategic Foresight: Knowledge, Tools, and Methods for the Future. ETH Zurich; 2021.

**65**. Centers for Disease Control and Prevention (CDC). History of Anthrax [updated 17 Jan 2023; cited 17 Jan 2023]. Available from: https://www.cdc.gov/anthrax/basics/anthrax-history.html.

**66**. World Health Organization. Health aspects of chemical and biological weapons: report of a WHO group of consultants. ; 1970.

**67**. Leitenberg M. Biological Weapons in the Twentieth Century: A Review and Analysis. Crit Rev Microbiol. 2001; 27:267–320.

**68**. Beedham RJ, Davies CH. The UK biological-warfare program: dual-use contributions to the field of aerobiology. The Nonproliferation Review. 2020; 27:309–22. doi: 10.1080/10736700.2020.1823621.

**69**. Bernstein BJ. The Birth of the U.S. Biological-Warfare Program. Scientific American. 1987; 265:116–21.

**70**. Roffey R, Tegnell A, Elgh F. Biological warfare in a historical perspective. Clinical microbiology and infection: The official publication of the European Society of Clinical Microbiology and Infectious Diseases. 2002; 8:450–4. doi: 10.1046/j.1469-0691.2002.00501.x PMID: 12197867.

**71**. Meselson M, Guillemin J, Hugh-Jones M, Langmuir A, Popova l, Shelokov A, et al. The Sverdlovsk Anthrax Outbreak of 1979. Science. 1994; 266:1202-1208.

**72**. Splino M, Patocka J, Prymula R, Chlibek R. Anthrax vaccines. Ann Saudi Med. 2005; 25:143–9. doi: 10.5144/0256-4947.2005.143 PMID: 15977694.

**73**. BWC. Final Declaration of the Third Review Conference. BWC/CONF.III/23. ; 1991.

**74**. Zilinskas RA. Iraq's Biological Weapons: The Past as Future. Jama. 1997; 278:418–24.

**75**. Mauroni A. On Biological War. MILITARY REVIEW. 2022. Available from: https://www.armyupress.army.mil/Portals/7/military-review/Archives/English/MJ-22/Mauroni/Mauroni.pdf.

**76**. Keim P, Smith KL, Keys C, Takahashi H, Kurata T, Kaufmann A. Molecular investigation of the Aum Shinrikyo anthrax release in Kameido, Japan. J Clin Microbiol. 2001; 39:4566–7. doi: 10.1128/JCM.39.12.4566-4567.2001 PMID: 11724885.

**77**. Cronin AK. Terrorist Motivations for Chemical and Biological Weapons Use: Placing the Threat in Context. Report for Congress. ; 23.03.2003.

**78**. Leitenberg M. Assessing the biological weapons and bioterrorism threat. Carlisle Barracks PA: Strategic Studies Institute U.S. Army War College; 2005.

**79**. Salama S, Hansell L. Does Intent equal Capability? Al-Qaeda and Weapons of Mass Destruction. The Nonproliferation Review. 2005; 12:615–53. doi: 10.1080/10736700600601236.

**80**. Spyer J. The Al-Qa'ida Network and Weapons of Mass Destruction. Middle East Review of International Affairs. 2004; 8:29–45.

**81**. Quintiliani Jr R, & Quintiliani R. Inhalational anthrax and bioterrorism. Current opinion in pulmonary medicine. 2003; 9.

**82**. Böhm R, Beyer W. Bioterroristische Anschläge mit *Bacillus anthracis*. Bundesgesundheitsblatt - Gesundheitsforschung - Gesundheitsschutz. 2003; 46:956–64. doi: 10.1007/s00103-003-0715-1.

**83**. Jackson BA. Technology Acquisition by Terrorist Groups: Threat Assessment Informed by Lessons from Private Sector Technology Adoption. Studies in Conflict & Terrorism. 2001; 24:183–213. doi: 10.1080/10576100151130270.

**84**. Revill J, Jefferson C. Tacit knowledge and the biological weapons regime. Science and Public Policy. 2014; 41:597–610. doi: 10.1093/scipol/sct090.

**85**. euroCRIS. UNESCO/COAR joint statement on Open Access | euroCRIS. 11.05.2016 [updated 31 Mar 2023; cited 31 Mar 2023]. Available from: https://eurocris.org/news/unescocoar-joint-statement-open-access.

**86**. Hughes RA, Ellington AD. Synthetic DNA Synthesis and Assembly: Putting the Synthetic in Synthetic Biology. Cold Spring Harb Perspect Biol. 2017; 9. Epub 2017/01/03. doi: 10.1101/cshperspect.a023812 PMID: 28049645.

**87**. Lentzos F. How to protect the world from ultra-targeted biological weapons. Bulletin of the Atomic Scientists. 2020; 76:302–8. doi: 10.1080/00963402.2020.1846412.

**88**. Tennenbaum M, Kosal ME. The Interplay Between Frugal Science and Chemical and Biological Weapons: Investigating the Proliferation Risks of Technology Intended for Humanitarian, Disaster Response, and International Development Efforts. In: Kosal ME, editor. Proliferation of Weapons-and Dual-Use Technologies. Diplomatic, Information, Military, and Economic Approaches. Springer International Publishing.; 2021. pp. 134–52.

**89**. Seyfried G, Pei L, Schmidt M. European do-it-yourself (DIY) biology: beyond the hope, hype and horror. Bioessays. 2014; 36:548–51. Epub 2014/04/29. doi: 10.1002/bies.201300149 PMID: 24782329.

**90**. Tocchetti S, Aguiton SA. Is an FBI Agent a DIY Biologist Like Any Other? A Cultural Analysis of a Biosecurity Risk. Science, Technology, & Human Values. 2015; 40:825–53. doi: 10.1177/0162243915589634.

**91**. Nixdorff K. Developments in systems biology: implications for health and biochemical security. The Nonproliferation Review. 2020; 27:459–73. doi: 10.1080/10736700.2020.1865632.

**92**. Vogel K. Bioweapons Proliferation: Where Science Studies and Public Policy Collide. Soc Stud Sci. 2006; 36:659–90. doi: 10.1177/0306312706059460.

**93**. Mondange L, Tessier É, Tournier J-N. Pathogenic Bacilli as an Emerging Biothreat. Pathogens. 2022; 11. Epub 2022/10/14. doi: 10.3390/pathogens11101186 PMID: 36297243.

**94**. Morris SA, Boyack KW. Visualizing 60 Years of Anthrax Research. Proceedings of ISSI. 2005:45–55.

**95**. CDC. Select Agents and Toxins List. HHS and USDA Select Agents and Toxins 7CFR Part 331, 9 CFR Part 121, and 42 CFR Part 73. 2023. Available from: https://www.selectagents.gov/sat/list.htm.

**96**. Sharan Y. The Bioterrorism Threat. In: Green MS, Zenilman J, Cohenn Dani, Wiser I, Balicer RD, editors. Risk assessment and risk communication strategies in bioterrorism preparedness. Dordrecht: Springer; 2007. pp. 45–54.

**97**. Green MS, Zenilman J, Cohenn Dani, Wiser I, Balicer RD, editors. Risk assessment and risk communication strategies in bioterrorism preparedness. Dordrecht: Springer; 2007.

**98**. Bulletin of the Atomic Scientists. Global Biolabs. Tracking maximum containment labs around the world. 2022 [cited 31 Mar 2023]. Available from: https://thebulletin.org/global-biolabs/.

**99**. Chacron MJ, Doiron B, Maler L, Longtin A, Bastian J. Non-classical receptive field mediates switch in a sensory neuron's frequency tuning. Nature. 2003; 423:77–81. doi: 10.1038/nature01590 PMID: 12721628.

**100**. Green BD, Battisti L, Koehler TM, Thorne CB, Ivins BE. Demonstration of a capsule plasmid in *Bacillus anthracis*. INFECTION AND IMMUNITY. 1985; 49:291–7. doi: 10.1128/iai.49.2.291-297.1985 PMID: 3926644.

**101**. Makino S, Uchida I, Terakado N, Sasakawa C, Yoshikawa M. Molecular characterization and protein analysis of the cap region, which is essential for encapsulation in *Bacillus anthracis*. J Bacteriol. 1989; 171:722–30. doi: 10.1128/jb.171.2.722-730.1989 PMID: 2536679.

**102**. Okinaka RT, Cloud K, Hampton O, Hoffmaster AR, Hill KK, Keim P, et al. Sequence and organization of pXO1, the large *Bacillus anthracis* plasmid harboring the anthrax toxin genes. J Bacteriol. 1999; 181:6509–15. doi: 10.1128/JB.181.20.6509-6515.1999 PMID: 10515943.

**103**. Pilcher HR. Anthrax genome unveiled. Nature. 2003. doi: 10.1038/news030428-14.

**104**. Read TD, Salzberg SL, Pop M, Shumway M, Umayam L, Jiang L, et al. Comparative genome sequencing for discovery of novel polymorphisms in *Bacillus anthracis*. Science. 2002; 296:2028–33. doi: 10.1038/news020506-9.

**105**. Wang Y, Wang D, Wang X, Tao H, Feng E, Zhu L, et al. Highly Efficient Genome Engineering in *Bacillus anthracis* and *Bacillus cereus* Using the CRISPR/Cas9 System. Front Microbiol. 2019; 10:1932. Epub 2019/08/27. doi: 10.3389/fmicb.2019.01932 PMID: 31551942.

**106**. Baldwin VM. You Can't *B. cereus* - A Review of *Bacillus cereus* Strains That Cause Anthrax-Like Disease. Front Microbiol. 2020; 11:1731. Epub 2020/08/19. doi: 10.3389/fmicb.2020.01731 PMID: 32973690.

**107**. Marston CK, Ibrahim H, Lee P, Churchwell G, Gumke M, Stanek D, et al. Anthrax Toxin-Expressing *Bacillus cereus* Isolated from an Anthrax-Like Eschar. PLoS One. 2016; 11:e0156987. Epub 2016/06/03. doi: 10.1371/journal.pone.0156987 PMID: 27257909.

**108**. Hoffmaster AR, Hill KK, Gee JE, Marston CK, De BK, Popovic T, et al. Characterization of *Bacillus cereus* isolates associated with fatal pneumonias: strains are closely related to *Bacillus anthracis* and harbor B. anthracis virulence genes. J Clin Microbiol. 2006; 44:3352–60. doi: 10.1128/JCM.00561-06 PMID: 16954272.

**109**. Cello J, Paul AV, Wimmer E. Chemical synthesis of poliovirus cDNA: generation of infectious virus in the absence of natural template. Science. 2002; 297:1016–8. Epub 2002/07/11. doi: 10.1126/science.1072266 PMID: 12114528.

**110**. Ehling-Schulz M, Lereclus D, Koehler TM. The *Bacillus cereus* Group: *Bacillus* Species with Pathogenic Potential. In: Fischetti VA, Novick RP, Ferretti JJ, Portnoy DA, Braunstein M, et al., editors. Gram-positive pathogens. Washington, DC: American Society for Microbiology; 2019. pp. 875–902.

**111**. Danzig R, Sageman M, Leighton T, Hough L, Yuki H, Kotani R, et al. Aum Shinrikyo Insights Into How Terrorists Develop Biological and Chemical Weapons. ; December 2012.

**112**. Stepanov AV, Marinin LI, Pomerantsev AP, Staritsin NA. Development of novel vaccines against anthrax in man. J Biotechnol. 1996; 44:155–60. doi: 10.1016/0168-1656(95)00092-5 PMID: 8717399.

**113**. Caruthers MH, Barone AD, Beaucage SL, Dodds DR, Fisher EF, McBride LJ, et al. Chemical synthesis of deoxyoligonucleotides by the phosphoramidite method. Methods Enzymol. 1987; 154:287–313. doi: 10.1016/0076-6879(87)54081-2 PMID: 3431460.

**114**. Stemmer WP, Crameri A, Ha KD, Brennan TM, Heyneker HL. Single-step assembly of a gene and entire plasmid from large numbers of oligodeoxyribonucleotides. Gene. 1995; 164:49–53. doi: 10.1016/0378-1119(95)00511-4 PMID: 7590320.

**115**. Kumar P, Ahuja N, Bhatnagar R. Purification of anthrax edema factor from Escherichia coli and identification of residues required for binding to anthrax protective antigen. INFECTION AND IMMUNITY. 2001; 69:6532–6. doi: 10.1128/IAI.69.10.6532-6536.2001 PMID: 11553601.

**116**. Robertson DL, Leppla SH. Molecular cloning and expression in *Escherichia coli* of the lethal factor gene of *Bacillus anthracis*. Gene. 1986; 44:71–8. doi: 10.1016/0378-1119(86)90044-2 PMID: 3021591.

**117**. Sharma M, Swain PK, Chopra AP, Chaudhary VK, Singh Y. Expression and purification of anthrax toxin protective antigen from Escherichia coli. Protein Expr Purif. 1996; 7:33–8. doi: 10.1006/prep.1996.0005 PMID: 9172780.

**118**. Gholami M, Moghbeli M, Kafilzadeh F, Kargar M, Torbati MB, Tavizi A, et al. Production of recombinant lethal factor of *Bacillus anthracis* in *Bacillus subtilis*. Prep Biochem Biotechnol. 2021; 51:9–15. Epub 2020/05/12. doi: 10.1080/10826068.2020.1762215 PMID: 32393098.

**119**. Gurkan C, Ellar DJ. Recombinant production of bacterial toxins and their derivatives in the methylotrophic yeast *Pichia pastoris*. Microb Cell Fact. 2005; 4:33. Epub 2005/12/07. doi: 10.1186/1475-2859-4-33 PMID: 16336647.

**120**. U.S. Department of Health & Human Services. Screening Framework Guidance for Providers of Synthetic Double-Stranded DNA. Fed Regist, 75(197), 62820-62832. ; 2010.

**121**. iGem Team:Lethbridge. Biosecurity software. 2017 [updated 3 May 2022; cited 31 Mar 2023]. Available from: https://2017.igem.org/Team:Lethbridge/Software.

**122**. EBRC Engineering Biology Research Consortium. Public Comment: Draft Revised Guidance Points of consideration from an EBRC Guidance Workshop. ; 2022.

**123**. Puzis R, Farbiash D, Brodt O, Elovici Y, Greenbaum D. Increased cyber-biosecurity for DNA synthesis. Nat Biotechnol. 2020; 38:1379–81. doi: 10.1038/s41587-020-00761-y PMID: 33247280.

**124**. Dassanayake MK, Khoo T-J, An J. Antibiotic resistance modifying ability of phytoextracts in anthrax biological agent *Bacillus anthracis* and emerging superbugs: a review of synergistic mechanisms. Ann Clin Microbiol Antimicrob. 2021; 20:79. Epub 2021/12/02. doi: 10.1186/s12941-021-00485-0 PMID: 34856999.

**125**. Leitenberg, M., & Zilinskas, R. A. The Soviet Biological Weapons Program: A History. In: Leitenberg M, Zilinskas RA, Kuhn JH, editors. The Soviet Biological Weapons Program: A History. ; 2012.

**126**. Dunlap G, Pauwels E. The Intelligent and Connected Bio-Labs of the Future: Promise and Peril in the Fourth Industrial Revolution. 2017 [cited 3 Nov 2022]. Available from: https://www.wilsoncenter.org/sites/default/files/media/documents/publication/dunlap_pauwels_intelligent_connected_biolabs_of_future.pdf.

**127**. Greenberg DL, Busch JD, Keim P, Wagner DM. Identifying experimental surrogates for *Bacillus anthracis* spores: a review. Investig Genet. 2010; 1:4. Epub 2010/09/01. doi: 10.1186/2041-2223-1-4 PMID: 21092338.

**128**. Tufts JAM, Calfee MW, Lee SD, Ryan SP. *Bacillus thuringiensis* as a surrogate for *Bacillus anthracis* in aerosol research. World J Microbiol Biotechnol. 2014; 30:1453–61. Epub 2013/12/12. doi: 10.1007/s11274-013-1576-x PMID: 24338558.

**129**. Lima-Pérez J, López-Pérez M, Viniegra-González G, Loera O. Solid-state fermentation of *Bacillus thuringiensis* var kurstaki HD-73 maintains higher biomass and spore yields as compared to submerged fermentation using the same media. Bioprocess Biosyst Eng. 2019; 42:1527–35. Epub 2019/05/21. doi: 10.1007/s00449-019-02150-5 PMID: 31115662.

**130**. Riesenberg D, Guthke R. High-cell-density cultivation of microorganisms. Appl Microbiol Biotechnol. 1999; 51:422–30. doi: 10.1007/s002530051412 PMID: 10341426.

**131**. Grossman, A. D., & Losick, R. Extracellular control of spore formation in *Bacillus subtilis*. Proceedings of the National Academy of Sciences. 1988; 85:4369–73. doi: 10.1073/pnas.85.12.436.

**132**. The Australia Group. Control List of Dual-use Biological Equipment and Related Technology and Software. 2021 [cited 3 Nov 2022]. Available from: https://www.dfat.gov.au/publications/minisite/theaustraliagroupnet/site/en/dual_biological.html.

**133**. Chen M, Lyu Y, Feng E, Zhu L, Pan C, Wang D, et al. SpoVG is Necessary for Sporulation in *Bacillus anthracis*. Microorganisms. 2020; 8. Epub 2020/04/10. doi: 10.3390/microorganisms8040548 PMID: 32290166.

**134**. Fetter S. Ballistic missiles and weapons of mass destruction: What is the threat? What should be done. International Security. 1991; 16:5–42.

**135**. Matsumoto G. Bioterrorism. Anthrax powder: state of the art. Science. 2003; 302:1492–7. doi: 10.1126/science.302.5650.1492 PMID: 14645823.

**136**. Zilinskas RA. The Soviet Biological Weapons Program and Its Legacy in Today’s Russia. National Defense University Press. 2014.

**137**. USDOJ. Amerithrax Investigative Summary. 2010. Available from: https://www.justice.gov/archive/amerithrax/docs/amx-investigative-summary.pdf.

**138**. European Union. Regulation (EU) 2021/821 of the European Parliament and of the Council of 20 May 2021 setting up a Union regime for the control of exports, brokering, technical assistance, transit and transfer of dual-use items (recast) PE/54/2020/REV/2. 2021. Available from: https://eur-lex.europa.eu/legal-content/EN/TXT/?uri=celex%3A32021R0821.

**139**. Epstein EJ. Edward Jay Epstein: The Anthrax Attacks Remain Unsolved. The Wall Street Journal. 2010 Jan 25. Available from: https://www.wsj.com/articles/SB10001424052748704541004575011421223515284 [updated 2010 Jan 25; cited 2023 Mar 31].

**140**. Bernstein R. Haste Leaves Anthrax Case Unconcluded. The New York Times. 2010 Feb 24. Available from: https://www.nytimes.com/2010/02/25/us/25iht-letter.html [updated 2010 Feb 24; cited 2023 Mar 31].

**141**. Rosenberg BH. Analysis of the anthrax attacks. Federation of American Scientists. 2002; 9.

**142**. Balkundi SS, Veerabadran NG, Eby DM, Johnson GR, Lvov YM. Encapsulation of bacterial spores in nanoorganized polyelectrolyte shells. Langmuir. 2009; 25:14011–6. doi: 10.1021/la900971h PMID: 19469562.

**143**. U.S. Congress, Office of Technology Assessment. Technologies Underlying Weapons of Mass Destruction. ; 1993.

**144**. Kaufmann AF, Meltzer MI, Schmid GP. The economic impact of a bioterrorist attack: are prevention and postattack intervention programs justifiable. Emerging Infectious Diseases. 1997; 3:83–94. doi: 10.3201/eid0302.970201 PMID: 9204289.

**145**. Aduojo EE, Amina SB, Kemi O, Jabir A. Bioterrorism and Biodefence: Biotechnology and Security Implications for Nigeria. American J Bioterror Biosecur Biodefens. 2022; 5:1–5. Available from: https://www.researchgate.net/profile/Amina-Bature/publication/360121455_Bioterrorism_and_Biodefence_Biotechnology_and_Security_Implications_for_Nigeria/links/62632d6ebca601538b60bbd1/Bioterrorism-and-Biodefence-Biotechnology-and-Security-Implications-for-Nigeria.pdf.

**146**. Haas CN. The role of risk analysis in understanding bioterrorism. Risk Anal. 2002; 22:671–7. doi: 10.1111/0272-4332.00239 PMID: 12224741.

**147**. Durrant GR. Bioterrorism: the current threat. Journal of the Royal Society of Medicine. 2002; 95:609–11.

**148**. Pethő-Kiss K. Addressing the Threat of a bioterrorist Attack by Means of an Unmanned Drone. Journal of Applied Security Research. 2022:1–24. doi: 10.1080/19361610.2021.2018923.

**149**. Blatny JM. DETECTING AND RESPONDING TO BIOTERRORISM. In: Green M, editor. Risk assessment and risk communication strategies in bioterrorism preparedness. Dordrecht: Springer; 2007.

**150**. Honein MA, Hoffmaster AR. Responding to the Threat Posed by Anthrax: Updated Evidence to Improve Preparedness. Clin Infect Dis. 2022; 75:S339-S340. doi: 10.1093/cid/ciac567 PMID: 36251547.

**151**. Rathish B, Pillay R, Wilson A, Pillay VV. Comprehensive Review Of Bioterrorism. 2022. Available from: https://www.ncbi.nlm.nih.gov/books/NBK570614/.

**152**. Beeching NJ, DavidABDance, Miller ARo, Spencer RC. Biological warfare and bioterrorism. BMJ Clinical Review. 2002; 324:336–9.

**153**. Cameron E, Katz R, Konyndyk J, Nalabandian M. A Spreading Plague: Lessons and Recommendations for Responding to a Deliberate Biological Event. ; 2019.

**154**. Rasko DA, Worsham PL, Abshire TG, Stanley ST, Bannan JD, Wilson MR, et al. *Bacillus anthracis* comparative genome analysis in support of the Amerithrax investigation. Proc Natl Acad Sci U S A. 2011; 108:5027–32. Epub 2011/03/07. doi: 10.1073/pnas.1016657108 PMID: 21383169.

**155**. Revill J, Borrie J, Lennane R. Back To The Future For Verification In The Biological Disarmament Regime. UNIDIR; 2022.

**156**. Schmedes S, Budowle B. Microbial Forensics. Schmedes, S., & Budowle, B. (2019). Microbial forensics. Encyclopedia of Microbiology, . Encyclopedia of Microbiology. 2019:134–45. doi: 10.1016/B978-0-12-801238-3.02483-1.

**157**. Hao R-Z, Song H-B, Zuo G-M, Yang R-F, Wei H-P, Wang D-B, et al. DNA probe functionalized QCM biosensor based on gold nanoparticle amplification for *Bacillus anthracis* detection. Biosens Bioelectron. 2011; 26:3398–404. Epub 2011/01/13. doi: 10.1016/j.bios.2011.01.010 PMID: 21315574.

**158**. Pal S, Alocilja EC. Electrically active magnetic nanoparticles as novel concentrator and electrochemical redox transducer in *Bacillus anthracis* DNA detection. Biosens Bioelectron. 2010; 26:1624–30. Epub 2010/08/20. doi: 10.1016/j.bios.2010.08.035 PMID: 20864333.

**159**. Kaittanis C, Santra S, Santiesteban OJ, Henderson TJ, Perez JM. The assembly state between magnetic nanosensors and their targets orchestrates their magnetic relaxation response. J Am Chem Soc. 2011; 133:3668–76. Epub 2011/02/22. doi: 10.1021/ja1109584 PMID: 21341659.

**160**. Boyer AE, Quinn CP, Woolfitt AR, Pirkle JL, McWilliams LG, Stamey KL, et al. Detection and quantification of anthrax lethal factor in serum by mass spectrometry. Anal Chem. 2007; 79:8463–70. Epub 2007/10/12. doi: 10.1021/ac701741s PMID: 17929949.

**161**. Duriez E, Goossens PL, Becher F, Ezan E. Femtomolar detection of the anthrax edema factor in human and animal plasma. Anal Chem. 2009; 81:5935–41. doi: 10.1021/ac900827s PMID: 19522516.

**162**. Kuklenyik Z, Boyer AE, Lins R, Quinn CP, Gallegos-Candela M, Woolfitt A, et al. Comparison of MALDI-TOF-MS and HPLC-ESI-MS/MS for endopeptidase activity-based quantification of Anthrax lethal factor in serum. Anal Chem. 2011; 83:1760–5. Epub 2011/02/08. doi: 10.1021/ac1030144 PMID: 21302970.

**163**. Čapek P, Kirkconnell KS, Dickerson TJ. A bacteriophage-based platform for rapid trace detection of proteases. J Am Chem Soc. 2010; 132:13126–8. doi: 10.1021/ja104572f PMID: 20812737.

**164**. Biagini RE, Sammons DL, Smith JP, MacKenzie BA, Striley CAF, Snawder JE, et al. Rapid, sensitive, and specific lateral-flow immunochromatographic device to measure anti-anthrax protective antigen immunoglobulin g in serum and whole blood. Clin Vaccine Immunol. 2006; 13:541–6. doi: 10.1128/CVI.13.5.541-546.2006 PMID: 16682473.

**165**. Campbell GA, Mutharasan R. Piezoelectric-excited millimeter-sized cantilever (PEMC) sensors detect *Bacillus anthracis* at 300 spores/mL. Biosens Bioelectron. 2006; 21:1684–92. Epub 2005/09/19. doi: 10.1016/j.bios.2005.08.001 PMID: 16169715.

**166**. De BK, Bragg SL, Sanden GN, Wilson KE, Diem LA, Marston CK, et al. A two-component direct fluorescent-antibody assay for rapid identification of *Bacillus anthracis*. Emerging Infectious Diseases. 2002; 8:1060–5. doi: 10.3201/eid0810.020392 PMID: 12396916.

**167**. Hao R, Wang D, Zhang X, Zuo G, Wei H, Yang R, et al. Rapid detection of *Bacillus anthracis* using monoclonal antibody functionalized QCM sensor. Biosens Bioelectron. 2009; 24:1330–5. Epub 2008/08/13. doi: 10.1016/j.bios.2008.07.071 PMID: 18804365.

**168**. McGovern J-P, Shih WY, Shih W-H. In situ detection of *Bacillus anthracis* spores using fully submersible, self-exciting, self-sensing PMN-PT/Sn piezoelectric microcantilevers. Analyst. 2007; 132:777–83. Epub 2007/06/18. doi: 10.1039/B704579D PMID: 17646877.

**169**. Mwilu SK, Aluoch AO, Miller S, Wong P, Sadik OA, Fatah AA, et al. Identification and quantitation of *Bacillus globigii* using metal enhanced electrochemical detection and capillary biosensor. Anal Chem. 2009; 81:7561–70. doi: 10.1021/ac900834e PMID: 19689112.

**170**. Tang S, Moayeri M, Chen Z, Harma H, Zhao J, Hu H, et al. Detection of anthrax toxin by an ultrasensitive immunoassay using europium nanoparticles. Clin Vaccine Immunol. 2009; 16:408–13. Epub 2009/01/07. doi: 10.1128/CVI.00412-08 PMID: 19129473.

**171**. Atabakhshi-Kashi M, Geranpayehvaghei M, Wang Y, Akhbariyoon H, Taleb M, Zhang Y, et al. Recent Advances of Nanocarriers for Effective Delivery of Therapeutic Peptides. Precision Nanomedicine. 2020; 3. doi: 10.33218/001c.13444.

**172**. Wang M. From home range dynamics to population cycles: validation and realism of a common vole population model for pesticide risk assessment. Integrated Environmental Assessment and Management. 2013; 9:294–307. Epub 2013/02/19. doi: 10.1002/ieam.1377 PMID: 23086922.

**173**. Zahavy E, Heleg-Shabtai V, Zafrani Y, Marciano D, Yitzhaki S. Application of fluorescent nanocrystals (q-dots) for the detection of pathogenic bacteria by flow-cytometry. J Fluoresc. 2010; 20:389–99. Epub 2009/10/14. doi: 10.1007/s10895-009-0546-z PMID: 19826932.

**174**. Fujinami Y, Hirai Y, Sakai I, Yoshino M, Yasuda J. Sensitive detection of *Bacillus anthracis* using a binding protein originating from gamma-phage. Microbiol Immunol. 2007; 51:163–9. doi: 10.1111/j.1348-0421.2007.tb03894.x PMID: 17310083.

**175**. Schuch R, Nelson D, Fischetti VA. A bacteriolytic agent that detects and kills *Bacillus anthracis*. Nature. 2002; 418:884–9. doi: 10.1038/nature01026 PMID: 12192412.

**176**. Acharya G, Doorneweerd DD, Chang C-L, Henne WA, Low PS, Savran CA. Label-free optical detection of anthrax-causing spores. J Am Chem Soc. 2007; 129:732–3. doi: 10.1021/ja0656649 PMID: 17243788.

**177**. Park H-Y, Go H-Y, Kalme S, Mane RS, Han S-H, Yoon M-Y. Protective antigen detection using horizontally stacked hexagonal ZnO platelets. Anal Chem. 2009; 81:4280–4. doi: 10.1021/ac900632n PMID: 19400578.

**178**. Cella LN, Sanchez P, Zhong W, Myung NV, Chen W, Mulchandani A. Nano aptasensor for protective antigen toxin of anthrax. Anal Chem. 2010; 82:2042–7. doi: 10.1021/ac902791q PMID: 20136122.

**179**. Huan TN, Ha VTT, Le Hung Q, Yoon M-Y, Han S-H, Chung H. Square wave voltammetric detection of Anthrax utilizing a peptide for selective recognition of a protein biomarker. Biosens Bioelectron. 2009; 25:469–74. Epub 2009/08/07. doi: 10.1016/j.bios.2009.08.002 PMID: 19729294.

**180**. Alibek K, Handelman S. Biohazard: The Chilling True Story of the Largest Covert Biological Weapons Program in the World -- Told from the Inside by the Man Who Ran It; Biological Weapons: Limiting the Threat. New York, New York 10036: Random House; 1999.

**181**. Kim D-J, Park H-C, Sohn IY, Jung J-H, Yoon OJ, Park J-S, et al. Electrical graphene aptasensor for ultra-sensitive detection of anthrax toxin with amplified signal transduction. Small. 2013; 9:3352–60. Epub 2013/04/16. doi: 10.1002/smll.201203245 PMID: 23589198.

**182**. Oh BN, Lee S, Park H-Y, Baeg J-O, Yoon M-Y, Kim J. Sensitive fluorescence assay of anthrax protective antigen with two new DNA aptamers and their binding properties. Analyst. 2011; 136:3384–8. Epub 2011/07/11. doi: 10.1039/c0an00978d PMID: 21743920.

**183**. Zhang N, Appella DH. Colorimetric detection of anthrax DNA with a Peptide nucleic acid sandwich-hybridization assay. J Am Chem Soc. 2007; 129:8424–5. Epub 2007/06/15. doi: 10.1021/ja072744j PMID: 17569540.

**184**. Kim J, Gedi V, Lee S-C, Cho J-H, Moon J-Y, Yoon M-Y. Advances in Anthrax Detection: Overview of Bioprobes and Biosensors. Appl Biochem Biotechnol. 2015; 176:957–77. Epub 2015/05/19. doi: 10.1007/s12010-015-1625-z PMID: 25987133.

**185**. Wang D-B, Cui M-M, Li M, Zhang X-E. Biosensors for the Detection of *Bacillus anthracis*. Acc Chem Res. 2021; 54:4451–61. Epub 2021/11/30. doi: 10.1021/acs.accounts.1c00407 PMID: 34846836.

**186**. Xu J, Bai X, Zhang X, Yuan B, Lin L, Guo Y, et al. Development and application of DETECTR-based rapid detection for pathogenic *Bacillus anthracis*. Anal Chim Acta. 2023; 1247:340891. Epub 2023/01/30. doi: 10.1016/j.aca.2023.340891 PMID: 36781250.

**187**. US Congress. PUBLIC LAW 108–276 118 STAT. 835. 2004. Available from: https://www.govinfo.gov/content/pkg/PLAW-108publ276/pdf/PLAW-108publ276.pdf.

**188**. Scales SE, Horney JA. Federal, state, local, and other public health agency roles in preparedness. 2023:11–34. doi: 10.1016/B978-0-323-98810-0.00002-8.

**189**. ECDC. COMMUNICABLE DISEASE THREATS REPORT. ; 2022.

**190**. Australian Government Department of Health. Anthrax: Public health response plan for Australia. Guidelines for preparedness, response and management following the deliberate release of Bacillus anthracis. Second edition. ; 2012.

**191**. Biselli R, Nisini R, Lista F, Autore A, Lastilla M, Lorenzo G de, et al. A Historical Review of Military Medical Strategies for Fighting Infectious Diseases: From Battlefields to Global Health. Biomedicines. 2022; 10. Epub 2022/08/22. doi: 10.3390/biomedicines10082050 PMID: 36009598.

**192**. Smith H, Keppie J, Stanley J. The chemical basis of the virulence of *Bacillus anthracis*. I. Properties of bacteria grown in vivo and preparation of extracts. Br J Exp Pathol. 1953; 34:477–85.

**193**. Stanley JL, Smith H. Purification of factor I and recognition of a third factor of the anthrax toxin. J Gen Microbiol. 1961; 26:49–63. doi: 10.1099/00221287-26-1-49 PMID: 13916257.

**194**. Thorne CB. Biochemical properties of virulent and avirulent strains of *Bacillus anthracis*. Ann N Y Acad Sci. 1960; 88:1024–33.

**195**. Tournier J-N, Ulrich RG, Quesnel-Hellmann A, Mohamadzadeh M, Stiles BG. Anthrax, toxins and vaccines: a 125-year journey targeting *Bacillus anthracis*. Expert Rev Anti Infect Ther. 2009; 7:219–36. doi: 10.1586/14787210.7.2.219 PMID: 19254170.

**196**. Tournier J-N, Mohamadzadeh M. Key roles of dendritic cells in lung infection and improving anthrax vaccines. Trends Mol Med. 2010; 16:303–12. Epub 2010/06/01. doi: 10.1016/j.molmed.2010.04.006 PMID: 20554248.

**197**. ECDC. Scientific Opinion on the public health hazards to be covered by inspection of meat (bovine animals). EFSA Journal. 2013; 11:3266. doi: 10.2903/j.efsa.2013.3266.

**198**. Wolfe DN, Espeland EM, Gao Y, Di Lu, Blatner G, Amass K, et al. Evaluation of BioThrax® and AV7909 anthrax vaccines in adults 66 years of age or older. Vaccine. 2020; 38:7970–6. Epub 2020/10/28. doi: 10.1016/j.vaccine.2020.10.053 PMID: 33129609.

**199**. Inglesby TV, O'Toole T, Henderson DA, Bartlett JG, Ascher MS, Eitzen E, et al. Anthrax as a Biological Weapon, 2002: Updated Recommendations for Management. JAMA. 2002; 287:2236–52. Available from: https://jamanetwork.com/journals/jama/article-abstract/194886?casa_token=k4nZmKlEUccAAAAA:YYTX_xAapbWdfegBWn4yhkhT1nTHfcROMdHFlH52OSzdq06ra3CbgtWff-BfUeETPH6EX21B-A.

**200**. Pannifer AD, Wong TY, Schwarzenbacher R, Renatus M, Petosa C, Bienkowska J, et al. Crystal structure of the anthrax lethal factor. Nature. 2001; 414:229–33. doi: 10.1038/n35101998 PMID: 11700563.

**201**. Bradley KA, Mogridge J, Mourez M, Collier RJ, Young JA. Identification of the cellular receptor for anthrax toxin. Nature. 2001; 414:225–9. doi: 10.1038/n35101999 PMID: 11700562.

**202**. Moayeri M, Leppla SH, Vrentas C, Pomerantsev AP, Liu S. Anthrax Pathogenesis. Annu Rev Microbiol. 2015; 69:185–208. Epub 2015/07/16. doi: 10.1146/annurev-micro-091014-104523 PMID: 26195305.

**203**. Migone T-S, Bolmer S, Zhong J, Corey A, Vasconcelos D, Buccellato M, et al. Added benefit of raxibacumab to antibiotic treatment of inhalational anthrax. Antimicrob Agents Chemother. 2015; 59:1145–51. Epub 2014/12/08. doi: 10.1128/AAC.04606-14 PMID: 25487792.

**204**. Avril A, Tournier J-N, Paucod J-C, Fournes B, Thullier P, Pelat T. Antibodies against Anthrax Toxins: A Long Way from Benchlab to the Bedside. Toxins (Basel). 2022; 14. Epub 2022/02/25. doi: 10.3390/toxins14030172 PMID: 35324669.

**205**. Huang E, Pillai SK, Bower WA, Hendricks KA, Guarnizo JT, Hoyle JD, et al. Antitoxin Treatment of Inhalation Anthrax: A Systematic Review. Health Secur. 2015; 13:365–77. doi: 10.1089/hs.2015.0032 PMID: 26690378.

**206**. Tournier J-N, Rougeaux C, Biot FV, Goossens PL. Questionable Efficacy of Therapeutic Antibodies in the Treatment of Anthrax. mSphere. 2019; 4. Epub 2019/06/19. doi: 10.1128/mSphere.00282-19 PMID: 31217301.

**207**. Urban-Sorensen K. Biological Warfare and Environmental Decontamination Post-Exposure: Who Is Responsible. Journal of Biosecurity, Biosafety, and Biodefense Law. 2018; 9. doi: 10.1515/jbbbl-2018-0007.

**208**. Canter DA, Gunning D, Rodgers P, O'connor L, Traunero C, Kempter CJ. Remediation of *Bacillus anthracis* contamination in the U.S. Department of Justice mail facility. Biosecurity and Bioterrorism: Biodefense Strategy, Practice, and Science. 2005; 3:119–27. doi: 10.1089/bsp.2005.3.119 PMID: 16000043.

**209**. Ginghina R-E, Toader G, Purica M, Bratu A-E, Lazaroaie C, Tiganescu T-V, et al. Antimicrobial Activity and Degradation Ability Study on Nanoparticle-Enriched Formulations Specially Designed for the Neutralization of Real and Simulated Biological and Chemical Warfare Agents. Pharmaceuticals (Basel). 2022; 15. Epub 2022/01/14. doi: 10.3390/ph15010097 PMID: 35056158.

**210**. Dong X, Katzbaer RR, Chitara B, Han L, Yang L, Schaak RE, et al. Optimizing the synergistic effect of CuWO 4 /CuS hybrid composites for photocatalytic inactivation of pathogenic bacteria. Environ Sci.: Nano. 2022; 9:4283–94. doi: 10.1039/D2EN00361A.

**211**. Nakonieczna A, Rutyna P, Fedorowicz M, Kwiatek M, Mizak L, Łobocka M. Three Novel Bacteriophages, J5a, F16Ba, and z1a, Specific for *Bacillus anthracis*, Define a New Clade of Historical Wbeta Phage Relatives. Viruses. 2022; 14. Epub 2022/01/21. doi: 10.3390/v14020213 PMID: 35215807.

**212**. Makino S, Sasakawa C, Uchida I, Terakado N, Yoshikawa M. Cloning and CO2-dependent expression of the genetic region for encapsulation from *Bacillus anthracis*. Mol Microbiol. 1988; 2:371–6. doi: 10.1111/j.1365-2958.1988.tb00041.x PMID: 2456447.

**213**. Zhang B, Li Y, Zhang Y, Qiao H, He J, Yuan Q, et al. High-cell-density culture enhances the antimicrobial and freshness effects of *Bacillus subtilis* S1702 on table grapes (Vitis vinifera cv. Kyoho). Food Chem. 2019; 286:541–9. Epub 2019/02/19. doi: 10.1016/j.foodchem.2019.02.050 PMID: 30827645.

**214**. Manoharan S, Taylor-Joyce G, Brooker TA, Hernández Rodríguez CS, Hapeshi A, Baldwin V, et al. From cereus to anthrax and back again: Assessment of the temperature-dependent phenotypic switching in the “cross-over” strain *Bacillus cereus* G9241. Front Microbiol. 2023; 14. doi: 10.3389/fmicb.2023.1113562.

**215**. GenBank. *Bacillus anthracis* edema factor gene (cya) gene, complete cds. 1995. Available from: https://www.ncbi.nlm.nih.gov/nuccore/M24074.1.

**216**. Chikerema SM, Pfukenyi DM, Hang'ombe BM, L'Abee-Lund TM, Matope G. Isolation of *Bacillus anthracis* from soil in selected high-risk areas of Zimbabwe. Journal of Applied Microbiology. 2012; 113:1389–95. Epub 2012/10/01. doi: 10.1111/jam.12006 PMID: 22984812.

**217**. Engineering Biology Research Consortium Security Working Group. Security Screening in Synthetic DNA Synthesis Recommendations for updated Federal Guidance. A Policy Paper by the Engineering Biology Research Consortium Security Working Group. 2022. Available from: https://ebrc.org/wp-content/uploads/2022/04/EBRC-2022-Security-Screening-in-Synthetic-DNA-Synthesis.pdf.

**218**. Atkins JF, Baranov PV. The distinction between recoding and codon reassignment. Genetics. 2010; 185:1535–6. doi: 10.1534/genetics.110.119016 PMID: 20713743.

**219**. Tian P, Ye W, Zhang X, Tong Y, Qian P-Y, Tong R. Ten-step asymmetric total syntheses of potent antibiotics anthracimycin and anthracimycin B. Chem Sci. 2022; 13:12776–81. Epub 2022/10/15. doi: 10.1039/d2sc05049h PMID: 36519065.

**220**. Hesse EM, Godfred-Cato S, Bower WA. Antitoxin Use in the Prevention and Treatment of Anthrax Disease: A Systematic Review. Clin Infect Dis. 2022; 75:S432-S440. doi: 10.1093/cid/ciac532 PMID: 36251559.

**221**. Deutsche Forschungsgemeinschaft and Deutsche Akademie der Naturforscher Leopoldina. Scientific Freedom and Scientific Responsibility Recommendations for Handling Security-Relevant Research. ; 2014.

**222**. Wang L, Song J, Zhang W. Tianjin Biosecurity Guidelines for codes of conduct for scientists: Promoting responsible sciences and strengthening biosecurity governance. Journal of Biosafety and Biosecurity. 2021; 3:82–3. doi: 10.1016/j.jobb.2021.08.001.

**223**. WHO. Global guidance framework for the responsible use of the life sciences: Mitigating biorisks and governing dual-use research. ; 2022.

**224**. National Research Council. Biotechnology Research in an Age of Terrorism. The National Academies Press; 2004.

**225**. Chen D-Y, Kenney D, Chin C-V, Tavares AH, Khan N, Conway HL, et al. Role of spike in the pathogenic and antigenic behavior of SARS-CoV-2 BA.1 Omicron. BioRxiv. 2023. Epub 2023/01/10. doi: 10.1101/2022.10.13.512134 PMID: 36263066.

**226**. Kaiser J. Making trouble. The United States is moving to tighten oversight of studies that could make viruses more dangerous. But how far should it go. Science. 2022; 378. doi: 10.1126/science.adf3764.

**227**. Reardon S. Stricter US guidelines for 'gain-of-function' research are on the way - maybe. Nature. 2023; 614:206–7. doi: 10.1038/d41586-023-00257-0 PMID: 36707714.

**228**. Epstein GL. Private-sector research could pose a pandemic risk. Here's what to do about it. Bulletin of the Atomic Scientists. 2023. Available from: https://thebulletin.org/2023/02/private-sector-research-could-pose-a-pandemic-risk-heres-what-to-do-about-it/#post-heading.

**229**. The Nuclear Threat Initiative. Preventing the Misuse of DNA Synthesis Technology [updated 17 Feb 2023; cited 31 Mar 2023]. Available from: https://www.nti.org/about/programs-projects/project/preventing-the-misuse-of-dna-synthesis-technology/.

**230**. LegiScan. California Assembly Bill 1963. CA State Legislature page for AB1963. 2022 [updated 28 Nov 2022; cited 31 Mar 2023]. Available from: https://legiscan.com/CA/text/AB1963/2021.

| 3D | Three dimensional |
| --- | --- |
| AV (46) | Anthrax vaccine |
| AVA | AV adsorbed |
| AVP | AV precipitated |
| BLAST | Basic Local Alignment Search Tool |
| CDC | Centerd for Disease Control and Prevention |
| CRISPR | Clustered regularly interspaced short palindromic repeats |
| CRISPR/Cas | Clustered regularly interspaced short palindromic repeats/CRISPR-associated protein 9 |
| dCas9 | deactivated CRISPR-associated protein 9 |
| DDBJ | DNA Data Bank of Japan |
| DNA | Deoxyribonucleic acid |
| EMBL | European Molecular Biology Laboratory |
| iGEM | International Genetically Engineered Machine |
| LAMP | loop-mediated isothermal amplification |
| NCBI | National Center for Biotechnology Information |
| NGS | Next-Generation Sequencing |
| PCR | Polymerase Chain Reaction |
| SERS | Surface-enhanced Raman spectroscopy |
| SynBio | Synthetic Biology |
| UK | United Kingdom |
| UN | United Nations |
| UNSC | United Nations Security Council |
| US | Unites States of America |
| USSR | Union of Soviet Socialist Republics |
| WHO | World Health Organization |
